# *Staphylococcus aureus* urease is controlled by a complex regulatory network to promote dissemination during CAUTI

**DOI:** 10.1128/mbio.01624-26

**Published:** 2026-08-05

**Authors:** Jana Gomez, Jesus Duran-Ramirez, Maristhela Alvarez, Shane Cristy, Jennifer N. Walker

**Affiliations:** 1 Department of Microbiology and Molecular Genetics, McGovern Medical School 12339 , Houston, Texas, USA; 2 The University of Texas MD Anderson Cancer Center UTHealth Houston Graduate School of Biomedical Sciences 182518 , Houston, Texas, USA; 3 Center for Infectious Diseases, Department of Epidemiology, UTHealth School of Public Health, University of Texas Health Science Center at Houston 12340, Houston, Texas, USA; University of Illinois Chicago, Chicago, Illinois, USA

**Keywords:** *Staphylococcus aureus*, urease, catheter-associated urinary tract infections, gene regulation

## Abstract

Catheter-associated urinary tract infections (CAUTIs) are one of the most common hospital-associated infections in the United States, accounting for >1 million cases annually. One CAUTI pathogen, *Staphylococcus aureus*, is commonly found persisting asymptomatically in the bladder of catheterized individuals, increasing these individuals’ risk of developing infection. Importantly, *S. aureus* is not only associated with severe symptoms during CAUTI, including bacteremia and septic shock, but it also produces a common uropathogen-associated virulence factor, urease. Despite its importance, urease has only been well-studied in another uropathogen, *Proteus mirabilis*. While previous studies identified three *S*. *aureus* urease regulators, including CodY, CcpA, and Agr, the environmental signals required for expression and activity, and the enzyme’s contribution to CAUTI have not been explored. In this study, we demonstrate that post-exponential growth and growth in the urinary tract environment significantly induce *S. aureus* urease expression and activity. Additionally, we identify SigB, SaeR, and SrrA as novel regulators of urease, and further explore the role of CodY, CcpA, and Agr—previously implicated in urease expression—in urease regulation in the urinary tract environment. Impactfully, we found that the urease promoters of clinical isolates contain genomic changes that enhance urease expression. Furthermore, urease contributes to biofilm formation under catheterized urinary tract-like conditions *in vitro* and dissemination from the bladder to the kidneys in a mouse CAUTI model. Together, our data not only provide insight into the regulatory pathway controlling *S. aureus* urease but also emphasize the importance of studying these mechanisms in a model that mimics the urinary environment.

**IMPORTANCE:**

In this study, we investigate how regulatory pathways coordinate the expression and activity of urease in response to environmental signals present within the catheterized urinary tract. We show that growth during the post-exponential phase and in conditions that mimic the urinary tract increases urease expression and activity. This finding challenges the dogma that *Staphylococcus aureus* is a “weak” urease producer. We also identified three novel regulators of *S. aureus* urease—SigB, SrrA, and SaeR—and show that their respective activation signals can modulate urease expression. Additionally, single-nucleotide changes identified in the urease regulatory pathway of clinical urinary catheter-associated isolates enhance urease expression. Finally, urease promotes biofilm formation under conditions that mimic the catheterized urinary tract and dissemination during catheter-associated urinary tract infections (CAUTIs). Our study provides insight into the complex regulatory mechanisms controlling urease in the urinary tract and highlights the role urease plays in *S. aureus* CAUTI.

## INTRODUCTION

Of the more than 30 million urinary catheters placed each year, 10%–30% become infected, translating to over 1 million cases of symptomatic catheter-associated urinary tract infections (CAUTIs) annually (1–7). The high infection rates combined with the frequency of catheter use have led to CAUTIs becoming the most common hospital-associated infection in the United States, with up to $1.83 billion a year in associated preventable costs (8–11). CAUTIs are also caused by a wide array of uropathogens, with no single pathogen accounting for more than 30% of infections, making empiric treatment challenging (7–11). Additionally, asymptomatic bacteriuria (ASB)—colonization of the bladder without symptoms—increases by 3%–8% each day of catheterization, resulting in virtually all long-term (>30 days) urinary catheters becoming colonized. Persistent ASB can increase the risk of developing a CAUTI and act as a reservoir for multidrug-resistant bacteria (8–12). Furthermore, recent studies have indicated that there is a further shift in organisms causing ASB compared to CAUTI, with gram-positive bacteria, including *Enterococcus faecalis* and *Staphylococcus aureus*, being some of the most frequent asymptomatic colonizers (9, 11).

Of the uropathogens that commonly result in catheter-associated ASB, a majority produce the enzyme urease, including *Proteus mirabilis*, *Klebsiella pneumoniae*, and *S. aureus*. Urease is a nickel-dependent metalloenzyme that hydrolyzes urea into ammonia and carbon dioxide, resulting in an alkaline environment that promotes the formation of precipitates (13–16). These precipitates can encrust urinary catheters and can contribute to catheter blockages, which lead to device failure and ultimately decrease the quality of life of individuals who are catheterized long-term, as they require more frequent catheter changes (13, 14, 17). Furthermore, catheter blockages can result in urine reflux to the kidneys, which increases the risk of secondary bacteremia (18, 19). Yet, the role of urease during the lifecycle of these pathogens in the catheterized bladder has only been well studied in the context of *P. mirabilis* CAUTI and ASB, leaving the other urease-producers largely uncharacterized. Notably, *S. aureus* is second only to *P. mirabilis* in causing catheter blockages (13, 20), which suggests that *S. aureus* contributes substantially to device blockages and ultimately, decreased quality of life. *S. aureus* is also associated with more severe disease during CAUTI, including high rates of secondary bacteremia and septic shock compared to other uropathogens (11, 21–23).

While some studies have implicated *S. aureus* urease as important for infection and fitness (24, 25), none have demonstrated a role in CAUTI (24, 25). Notably, despite the severity of *S. aureus* CAUTI, even fewer studies have investigated the pathogen’s lifecycle in the catheterized bladder or the contribution of urease to more severe patient outcomes (9–12, 26, 27). Additionally, previous studies investigating the regulatory pathways dictating *S. aureus* urease production demonstrate that several well-characterized regulators control expression of the enzyme (25, 28–30). Among these regulators are the accessory gene regulator (Agr) system—the quorum sensing system in *S. aureus* (31, 32), the transcriptional regulator CodY—a global regulator that responds to levels of branched-chain amino acids (BCAA) and GTP in the environment (33), and the carbon catabolite protein A (CcpA)—a carbon catabolite regulator that is activated in the presence of high levels of glucose in the environment (28, 34). Specifically, while mutants of *ccpA* and *agr* result in lower urease expression, a mutation in *codY* results in higher urease expression compared to the parent strains—demonstrating that these regulators activate and inhibit urease, respectively (26, 28). Additionally, other studies performing genome-wide analysis of *S. aureus* regulons have implicated other regulators of urease, such as the sigma factor SigB. Yet, the role of SigB in regulating urease has not been directly assessed (31). This suggests that Agr, CcpA, and CodY are not the sole regulators of *S. aureus* urease. Despite these studies, how *S. aureus* responds to the environmental signals in the catheterized bladder to coordinate the expression and activity of urease is unknown.

In this study, we investigated *S. aureus* urease and (i) determined expression and activity are induced under conditions that mimic the urinary tract; (ii) identified three novel regulators—SigB, SrrA, and SaeR; (iii) defined the signals within the urinary tract that *S. aureus* senses to coordinate the expression of the enzyme; (iv) discovered novel genomic changes within the urease promoters of clinical isolates that impact expression and activity; and (v) demonstrated the enzyme contributes to *S. aureus* biofilm and dissemination during CAUTI. Impactfully, these findings untangle the complex regulatory network dictating urease expression, which is required to most efficiently coordinate its expression in response to specific signals within the urinary tract. Additionally, this study demonstrates the importance of urease in *S. aureus* biofilm formation under conditions that mimic the catheterized bladder and spread from the bladder to other organs during CAUTI.

## RESULTS

### Growth phase is important for urease expression

**Fig 1:**
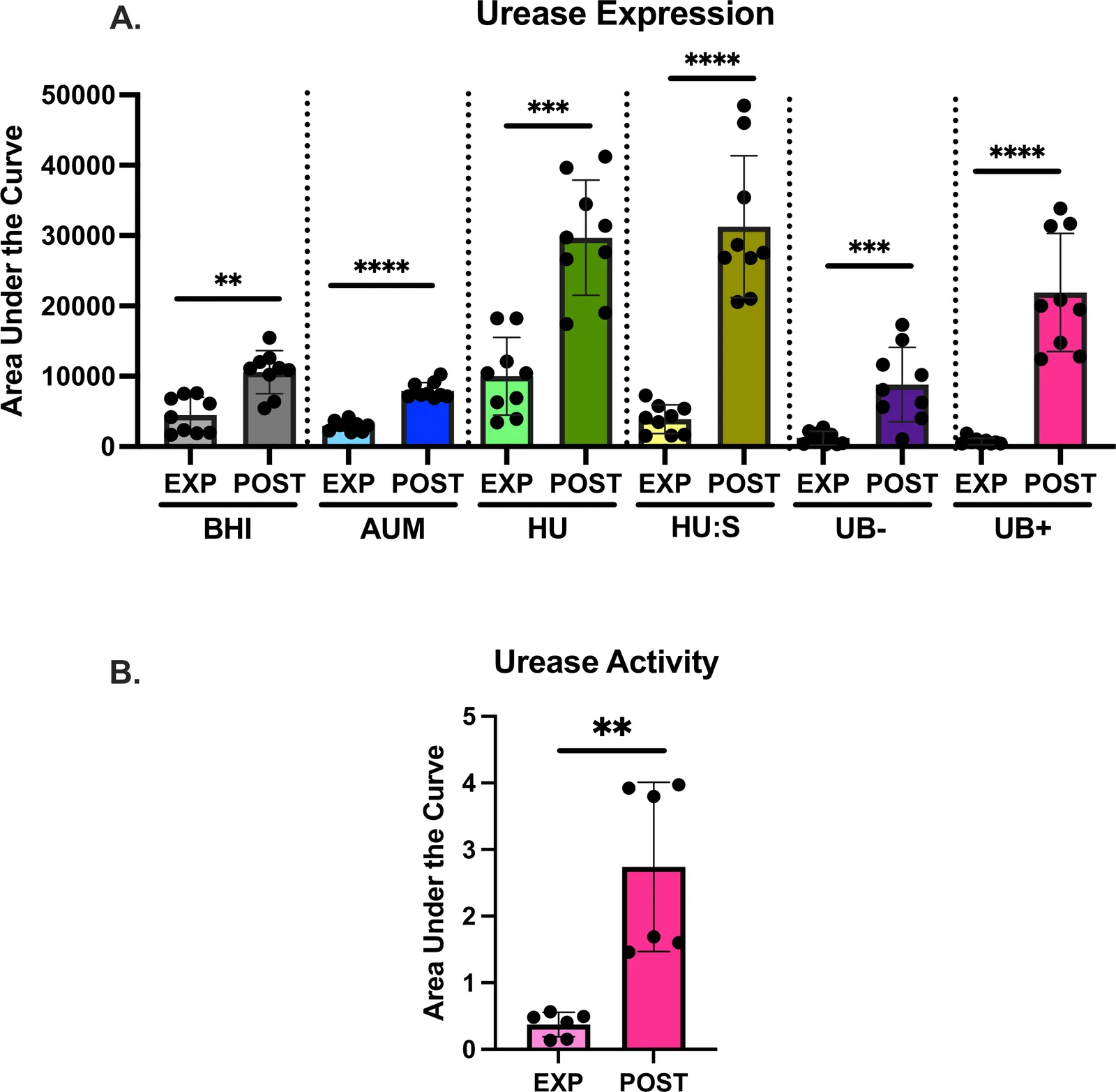
Urease expression and activity in JE2. (**A**) Urease expression was analyzed in exponential (EXP) and post-exponential (POST) phases in BHI, AUM, female HU, a 1:1 ratio of HU and saline (HU:S), urease broth without urea (UB−), and urease broth with urea (UB+). Fluorescence was normalized to the fluorescence reading of the background strain that did not contain the GFP reporter plasmid. Each dot represents a technical replicate. At least two biological replicates were performed. Bars represent the mean of all replicates with the standard deviation. The area under the curve for each strain is quantified, and significance was determined using the Mann-Whitney *U* test. (**B**) Urease activity was assessed in UB, with activity in UB+ normalized to UB− to account for any color change not specifically due to urease activity. Urease expression and activity were measured over a 12 h time course (35). Each dot represents a technical replicate. At least two biological replicates were performed. Bars represent the mean of all replicates with the standard deviation. The area under the curve for each strain is quantified, and significance was determined using the Mann-Whitney *U* test.

To determine whether *S. aureus* senses signals within the urinary tract environment that coordinate the expression of urease, a transcriptional reporter plasmid with the 200-base-pair region upstream of the urease operon (i.e., the urease promoter) fused to GFP (pUre1) was generated in the JE2 background strain (Fig. S1; File S2). The pUre1 plasmid did not alter the growth of these cells compared to the parent strain in rich medium (brain heart infusion [BHI]) or artificial urine medium (AUM)—a minimal, chemically defined, urine-like medium (Fig. S1B and C). Urease expression was then assessed across a 12 h time course in various media, including a nutrient-rich growth medium (BHI) and low nutrient, urinary tract-like medium conditions, such as AUM (Table 1), female human urine (HU), and urease broth (UB) (Fig. 1A; Fig. S2A and B). Strikingly, the post-exponential phase of growth displayed the most consistent impact on urease expression, where regardless of medium, there was significantly higher urease expression compared to the exponential growth phase (Fig. 1A; Fig. S2A and B). Furthermore, urease expression was significantly higher in HU compared to BHI and AUM (Fig. S2C). However, HU displayed high variation across biological and technical replicates. Together, these data suggest urease is most highly expressed during post-exponential growth under conditions that are present in the urinary tract.

**TABLE 1:** Composition of AUM

Chemical component	Amount
Sodium sulfate	11.965 mM
Uric acid	1.487 mM
Sodium citrate dihydrate	2.45 mM
Creatinine	7.791 mM
Urea	249.75 mM
Potassium chloride	30.953 mM
Sodium chloride	30.053 mM
Calcium chloride	1.1663 mM
Ammonium chloride	23.667 mM
Potassium oxalate monohydrate	0.19 mM
Magnesium sulfate heptahydrate	4.389 mM
Sodium phosphate monobasic	18.667 mM
Sodium phosphate dibasic	4.667 mM
Tryptone	0.909 mM
Yeast nitrogen broth without amino acids and without ammonium sulfate	0.17%
Glucose	0.833 mM
Biotin	82 nM

### Growth phase impacts urease activity

In addition to expression, we also assessed whether growth phase impacts urease activity (32). We used the previously published urease activity assay, which measures urease activity via the pH indicator phenol red to detect the pH change that occurs following urea breakdown by urease (36–38). HU has a broad pH range (pH 4–8), which can substantially affect the ability to determine when and how much urease activity is detected. Specifically, at pH 8, phenol red turns pink, preventing the detection of urease activity in high-pH urine pools. Furthermore, the use of a urea-negative medium is crucial for ensuring the color change observed is due to urease breakdown of urea specifically, and not due to other byproducts that are released during *S. aureus* growth that alter the pH (25). As such, urease activity was analyzed in urease broth with urea (UB+) and normalized to urease broth without urea (UB−) (39). Consistent with urease expression, activity was also significantly higher in the post-exponential phase compared to the exponential phase (Fig. 1B; Fig. S2D), emphasizing the importance of growth phase in regulating activity. Together, these data indicate that as *S. aureus* enters post-exponential phase, urease activity is also upregulated.

### Bioinformatic identification of regulator binding sites in the *S. aureus* urease promoter

**Fig 2:**
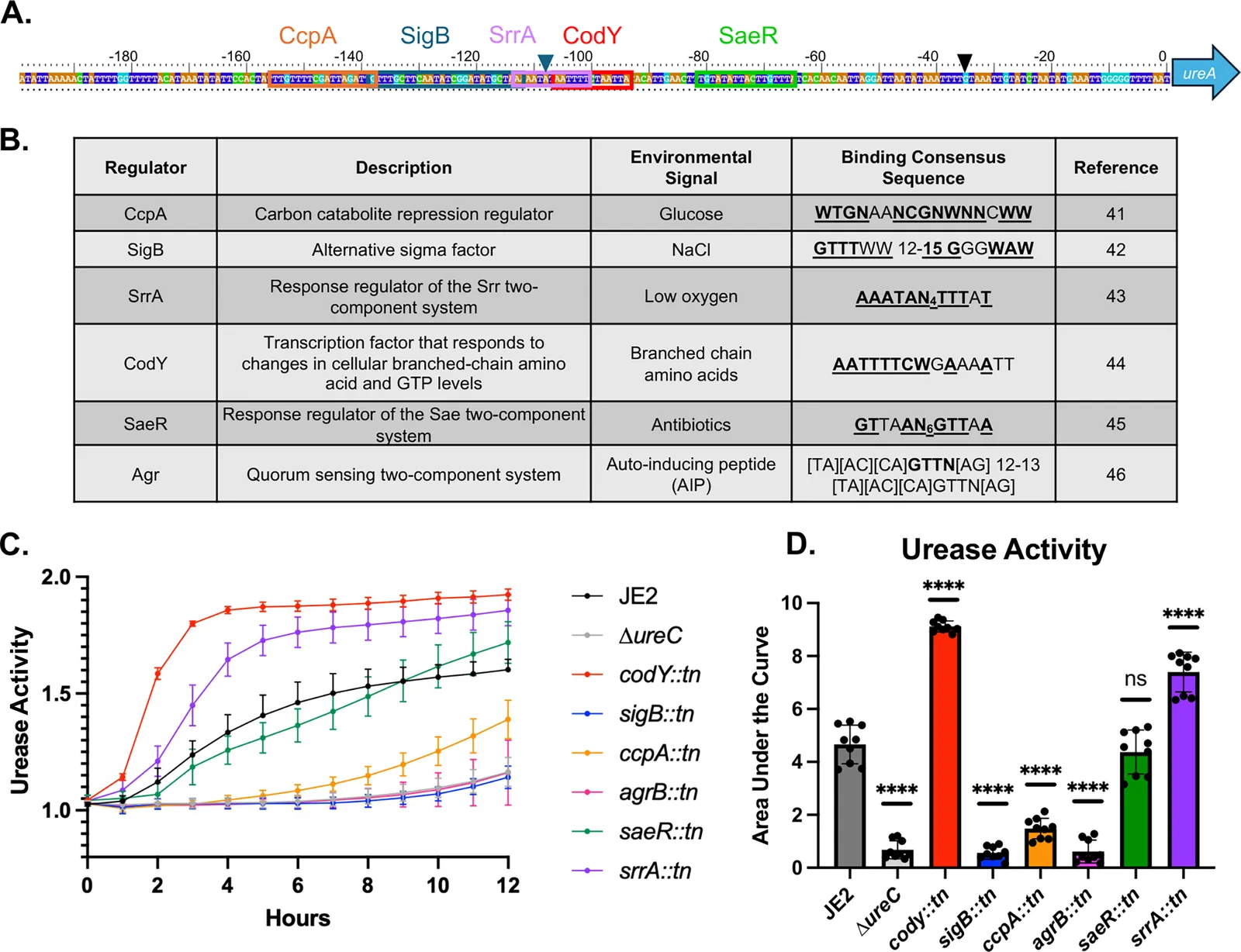
SigB and SrrA are novel regulators of urease activity in JE2. (**A**) The 200 bp region upstream of the urease operon (i.e., the urease promoter) in JE2. Predicted binding sites for transcriptional regulators are denoted in boxes. Transcriptional start sites for the predicted SigA-controlled transcript (black) and the predicted SigB-controlled transcript (blue) are denoted by triangles (45). (**B**) Table detailing features of each predicted regulator of urease. Binding consensus sequences and their references are listed. Bolded and underlined characters indicate an exact match within the JE2 urease promoter. W indicates either an A or a T. N indicates any nucleotide. (**C**) Urease activity in regulator mutants in the JE2 background. Urease activity was measured over a 12 h time course (35). Each dot represents a technical replicate. At least three biological replicates were performed. Bars represent the mean of all replicates with the standard deviation. (**D**) The area under the curve for each strain is quantified, and significance was determined using the Mann-Whitney *U* test, where asterisks indicate *P* < 0.05 and ns indicates *P* > 0.05 (no significance).

To identify potential regulators of urease, the urease promoter was analyzed for the presence of previously published regulator consensus binding sequences (39–44). Consistent with a previous report (45), two potential transcription start sites for the urease operon, one under the control of the primary sigma factor SigA and another under the control of the alternative sigma factor SigB, were identified (Fig. 2A and B). Additionally, five consensus binding sequences were identified, including two for the previously identified urease regulators CodY (25) and CcpA (25, 28), as well as two additional two-component system response regulators, SaeR and SrrA. Despite previous reports showing that Agr regulates urease expression (25), an AgrA consensus binding site was not observed in the urease promoter (Fig. 2A and B). These results suggest that Agr regulates urease through an indirect mechanism, which is not uncommon for the quorum sensing system (31, 46). Overall, the identification of the five consensus binding sequences in the urease promoter suggests that CodY, CcpA, SigB, SaeR, and SrrA all regulate urease expression and activity.

### Agr, CcpA, CodY, SigB, and SrrA regulate urease activity

To determine whether these predicted regulators control urease activity, loss-of-function mutations in each regulator were generated in the JE2 background strain (Fig. S3A). Since our data in Fig. 1 and our previously published work (35) indicate that there is no detectable urease activity during exponential growth, activity assays were performed in the post-exponential growth phase. Compared to JE2, significantly higher urease activity was observed in *codY::tn* and *srrA::tn* mutants, while significantly lower urease activity was observed in the *sigB::tn*, *ccpA::tn*, and *agrB::tn* mutants (Fig. 2C). The *saeR* mutant did not have any effect on urease activity in these conditions (Fig. 2C). Furthermore, these data demonstrate that CodY and SrrA repress activity, while SigB, CcpA, and AgrB activate activity. Importantly, this assay establishes that SigB and SrrA are two previously unrecognized regulators of *S. aureus* urease activity.

### Mutants of *agr*, *ccpA*, *codY*, *sigB*, *saeR*, and *srrA* grow in the urinary tract-like environment

To determine whether the urinary tract-like environment impacted growth of regulator mutants, growth was assessed in AUM and BHI (Fig. S3B through E). AUM was prioritized for urease expression experiments for two reasons: (i) AUM was required for experiments assessing urease expression in response to the addition of regulator signals, and (ii) it displayed a similar, yet more consistent, expression phenotype across biological and technical replicates compared to HU. The *ureC* mutation grew similarly to JE2 in BHI and AUM. However, compared to JE2 grown in BHI, the *codY*, *sigB*, *saeR*, and *srrA* mutants exhibited a small but significant growth increase, while the *ccpA* and *agrB* mutants displayed a small but significant decrease in growth (Fig. S3B and C). Compared to JE2 grown in AUM, the *codY* and *sigB* mutants exhibited a small but significant growth increase, while the *ccpA*, *agrB*, *saeR*, and *srrA* mutants displayed a small but significant growth defect (Fig. S3D and E). Together, these results demonstrate that all mutant strains grow in BHI and AUM.

### CodY, CcpA, Agr, SigB, SrrA, and SaeR control urease expression

**Fig 3:**
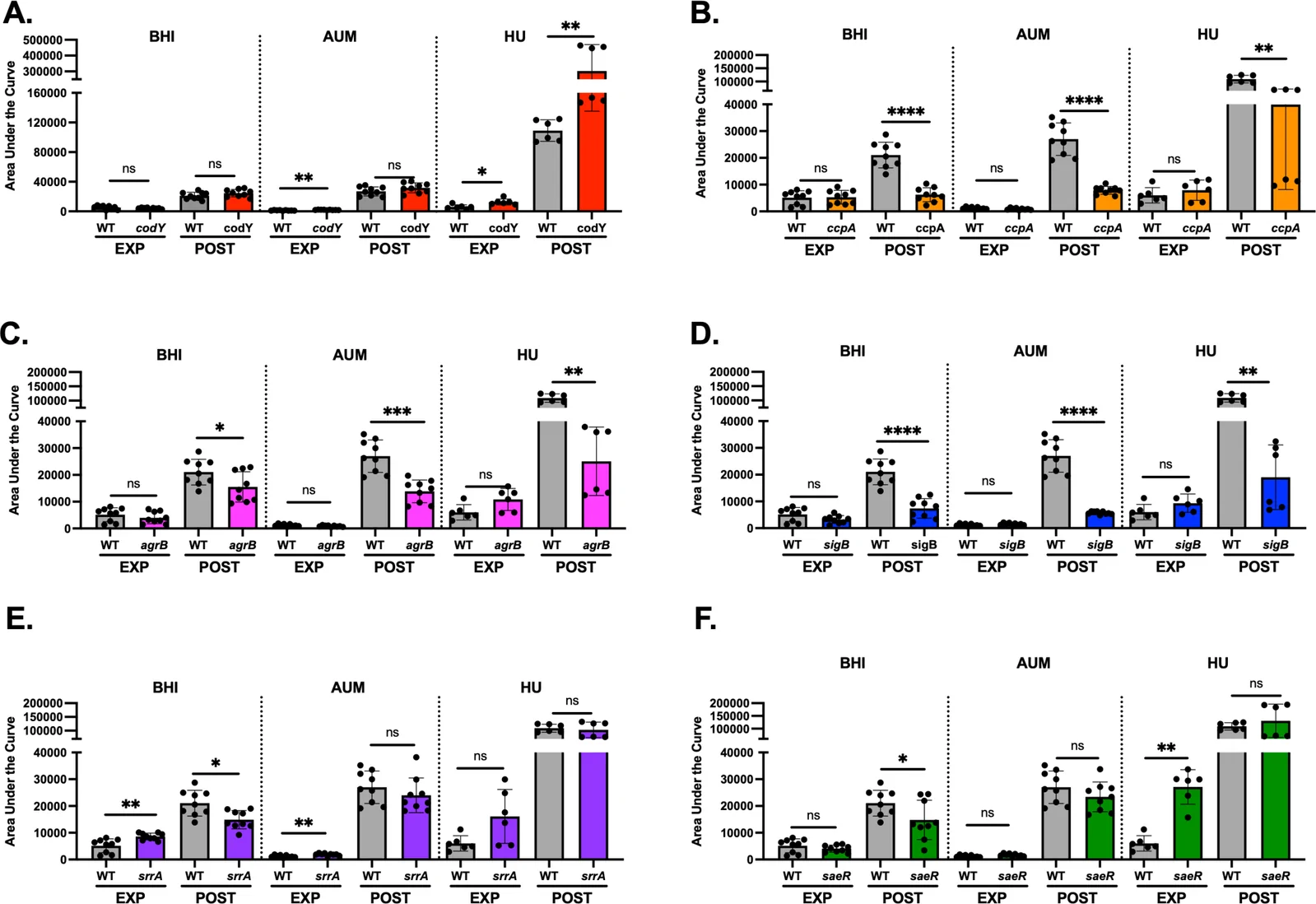
Regulator mutants alter urease expression in JE2. Urease expression in JE2 *codY::tn* (**A**), JE2 *ccpA::tn* (**B**), JE2 *agrB::tn* (**C**), JE2 *sigB::tn* (**D**), JE2 *srrA::tn* (**E**), and JE2 *saeR::tn* (**F**) at both exponential (EXP) and post-exponential (POST) phases, in either BHI, AUM, or HU over a 12 h time course. Fluorescence was normalized to the fluorescence reading of the background strain that did not contain the GFP reporter plasmid. Each dot represents a technical replicate. At least three biological replicates were performed. Bars represent the mean of all replicates with the standard deviation. The area under the curve for each strain was quantified, and significance was determined using the Mann-Whitney *U* test.

To determine whether SigB, SrrA, and SaeR impact urease expression and the role of the previously identified regulators (25, 28) in the urinary tract-like environment, each mutant in the JE2 background was transformed with the pUre1 plasmid and assessed for urease expression in BHI, AUM, and HU (Fig. 3; Fig. S4). The *codY*, *ccpA*, and *agrB* mutants were included to ensure our results with pUre1 in rich media were consistent with previously published work (25, 28) and to determine whether reported phenotypes were consistent across different medium types. Indeed, urease expression in the *codY* mutant was significantly increased compared to JE2 in both AUM and HU, with the highest expression observed in HU (Fig. 3A; Fig. S4). Furthermore, urease expression in the *ccpA* and *agrB* mutants was significantly lower compared to JE2 in all medium types tested (BHI, AUM, and HU), again with the biggest differences observed in HU (Fig. 3B and C; Fig. S4). These results correlate with the observed urease activity detected for each mutant in Fig. 2C. Importantly, this finding correlates with previous analyses of these urease regulators (25, 28).

Next, the novel urease regulators, SigB, SrrA, and SaeR, were assessed for their effects on urease expression (Fig. 3D through F; Fig. S4). The *sigB* mutant displayed significantly lower urease expression compared to JE2 in all medium types (Fig. 3D; Fig. S4). This finding correlates with the observed decrease in urease activity observed in the *sigB* mutant (Fig. 2C). In contrast, the *srrA* mutation displayed significantly higher urease expression compared to JE2 in BHI and AUM in the exponential phase (Fig. 3E; Fig. S4). Importantly, this finding correlates with the *srrA* mutant displaying higher urease activity compared to JE2 (Fig. 2C). Lastly, the *saeR* mutant displayed different phenotypes depending on the medium. In BHI, the *saeR* mutant displayed significantly lower urease expression compared to JE2 in the post-exponential phase (Fig. 3F; Fig. S4). In contrast, the *saeR* mutant displayed significantly higher urease expression compared to JE2 in HU in the exponential phase. Notably, urease expression did not always correlate with growth phenotypes. Specifically, the *sigB* mutant displayed significantly higher growth (Fig. S3B through E) but significantly lower urease expression in both BHI and AUM (Fig. 3D). Overall, these data demonstrate that SigB, SrrA, and SaeR regulate urease, and environmental conditions that mimic the urinary tract environment display the greatest impact on that regulation.

### Environmental signals present within the urinary tract impact urease expression

**Fig 4:**
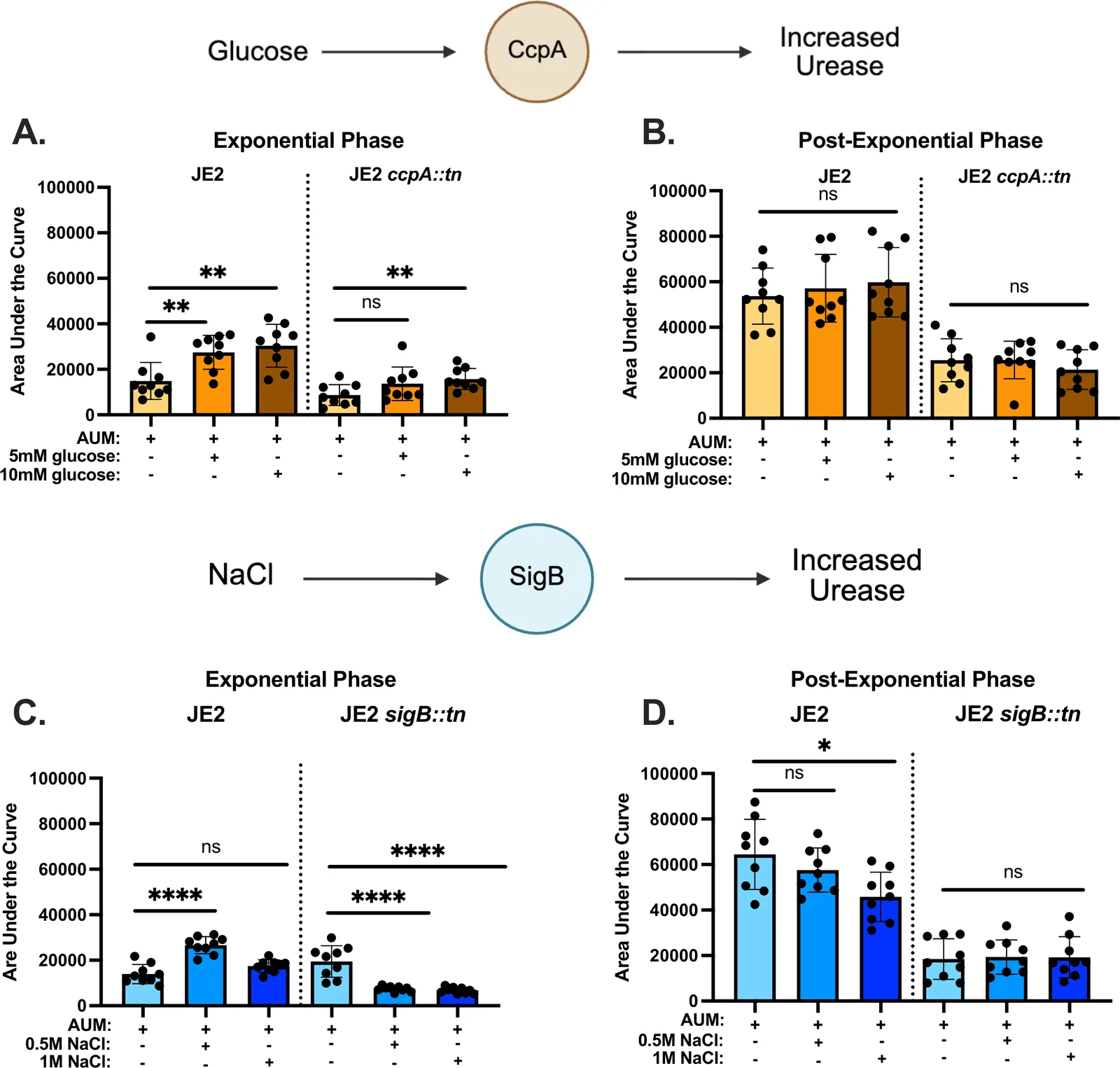
The impact of environmental signals on urease expression. Cells were grown in the presence of the CcpA signal glucose (**A, B**) or the SigB signal NaCl (**C, D**) in cells in the exponential (**A, C**) or post-exponential (**B, D**) phase. Fluorescence was normalized to the fluorescence reading of the background strain that did not contain the GFP reporter plasmid. Each dot represents a technical replicate. At least two total biological replicates were performed. Bars represent the mean of all replicates with the standard deviation. The area under the curve for each strain was quantified and compared for significance using the Mann-Whitney *U* test. Created in BioRender (J. Gomez, 2026, https://BioRender.com/9hvkgl2).

Since the environmental stimuli of each urease regulator, and specifically the newly identified urease regulators, are known, we utilized the pUre1 plasmid to measure urease expression in AUM and AUM supplemented with each of the corresponding stimuli, including glucose (CcpA) (28), NaCl (SigB) (47), BCAAs (CodY) (48), autoinducing peptide (AIP; Agr) (49), low oxygen (SrrA) (50), and antibiotics (SaeR) (51) (Fig. 4; Fig. S5 and S6). AUM was selected over HU for this experiment, since the signals for each of these regulators are already present within HU and they cannot be removed or accurately adjusted to specific concentrations. Similarly to the urease expression assays in the regulator mutant backgrounds, these assays were performed during both exponential and post-exponential growth.

#### The CcpA activator, glucose, modulates urease expression in a CcpA-dependent manner

CcpA has been previously shown to sense and activate genes in response to increasing levels of glucose (28). To determine if glucose induces urease, we analyzed expression in AUM and AUM supplemented with an additional 5 and 10 mM of glucose, which has been previously shown to activate genes in a CcpA-dependent manner (28). Importantly, *S. aureus* growth is significantly inhibited in the absence of glucose, thus these glucose levels were additive to the levels of glucose already present in AUM, which is low (0.83 mM, Table 1) (50). The addition of both 5 and 10 mM glucose in the exponential phase significantly increased JE2 urease expression in a dose-dependent manner compared to JE2 in AUM alone (Fig. 4A; Fig. S5A). This result suggests that glucose induces urease expression during the exponential phase. In contrast, the addition of glucose in the post-exponential phase did not alter JE2 urease expression compared to JE2 in AUM alone (Fig. 4B; Fig. S5B). This phenotype is likely due to the fact that urease is already highly expressed in JE2 during the post-exponential phase and therefore is not further induced. To determine if the increase in urease expression in response to glucose in the exponential phase was dependent on the CcpA regulator, the *ccpA* mutant was assessed. Urease expression in the *ccpA* mutant was similar in AUM alone and following the addition of 5 mM glucose in the exponential phase (Fig. 4A; Fig. S5A). Additionally, the addition of glucose during post-exponential growth did not alter urease expression compared to the *ccpA* mutant in AUM alone (Fig. 4B; Fig. S5B). Together, these results indicate that glucose activates urease expression in a CcpA-dependent manner during the exponential phase.

#### The SigB activator NaCl modulates urease expression in a SigB-dependent way

SigB has previously been shown to sense and activate genes in response to increasing concentrations of salt (47). To determine if NaCl induces urease, we analyzed expression in AUM and AUM supplemented with 0.5 and 1 M NaCl, which has previously been shown to activate genes in a SigB-dependent way (47). The addition of 0.5 M NaCl to the AUM increased urease expression in the exponential phase in JE2 compared to AUM alone (Fig. 4C; Fig. S5C). In contrast, the addition of 1 M NaCl decreased urease expression in the post-exponential phase compared to JE2 in AUM alone (Fig. 4D; Fig. S5D). This result was unexpected, as NaCl activation of SigB was expected to activate urease expression (Fig. 3D). To determine if the increased urease expression in the exponential phase was dependent on SigB, the *sigB* mutant was assessed. Urease expression was significantly decreased in AUM supplemented with 0.5 and 1 M of NaCl in the exponential phase compared to the *sigB* mutant in AUM alone (Fig. 4C; Fig. S5C). In contrast, the addition of NaCl did not alter urease expression in the post-exponential phase compared to the *sigB* mutant in AUM alone (Fig. 4D; Fig. S5D). Impactfully, while urease expression in the post-exponential phase in the *sigB* mutant was unaffected by the addition of NaCl, the increased urease expression observed during the exponential phase in JE2 grown in AUM supplemented with increasing concentrations of salt compared to AUM alone was ablated in the *sigB* mutant. Together, these results suggest that NaCl activates urease expression in the exponential phase in a SigB-dependent manner.

#### Other regulator signals modulate urease expression independent of their expected regulators

To determine if other signals modulate urease expression through their respective regulators, AUM supplemented with BCAAs (CodY), AIP (Agr), low oxygen concentrations (SrrA), or subinhibitory concentrations of vancomycin (SaeR) were assessed (Fig. S5 and S6). Briefly, urease expression was assessed in AUM lacking and AUM supplemented with (i) 10 mM of leucine (Leu), isoleucine (Ile), and valine (Val) (Fig. S5E and F and S6A and B), which have previously been shown to repress CodY-regulated genes (48); (ii) 10% and 20% spent supernatant from JE2 (AIP-containing supernatant) (Fig. S5E and F and S6A and B), which can activate the Agr regulon (49); (iii) 0.5 and 1 mM of the nitrous oxide donor DEA/NO (Fig. S5I and J and S6E and F), which induces a low oxygen environment to upregulate SrrA target genes (50); or (iv) 5, 10, or 15 ug/mL of vancomycin (Fig. S5K and L and S6G and H), which can either induce or inhibit the expression of genes in the SaeR regulon (51). All signals tested modulated urease expression in the exponential phase compared to JE2 in AUM alone (Fig. S5E, G, I, and K and S6A, C, E, and G). In contrast, only spent supernatant modulated urease expression in the post-exponential phase compared to JE2 in AUM alone (Fig. S5F, H, J, and L and S6B, D, F, and H). Notably, the addition of 20% spent supernatant from the *agr* mutant (AIP-negative) also significantly increased urease expression during post-exponential growth compared to JE2 in AUM alone (Fig. S5G and H and S6C and D). These results suggest that some component of the spent supernatant is contributing to the induction of urease expression, but that this effect may be independent of Agr in the post-exponential phase. Additionally, these data suggest that while BCAAs, low oxygen, and subinhibitory vancomycin concentrations modulate urease expression during the exponential phase, these signals have no effect on urease expression during the post-exponential phase.

To determine if these signals modulate urease through their respective regulators, the *codY*, *agr*, *srrA*, and *saeR* mutants were assessed (Fig. S5 and S6). Urease expression of each regulator mutant grown in AUM or AUM supplemented with the respective signal displayed similar phenotypes compared to JE2 in either the exponential or post-exponential phase. Together, these results indicate that the effect of BCAAs, AIP, low oxygen, and subinhibitory vancomycin concentrations on urease expression in JE2 is not mediated by CodY, Agr, SrrA, or SaeR, respectively. Notably, the crosstalk between Agr and the regulators tested here is well established, suggesting that the phenotypes observed here may be due to the wide range of interactions that Agr has with other known regulators of urease (31, 52–55).

### Urinary catheter-associated isolates contain genomic changes in the urease promoter

**Fig 5:**
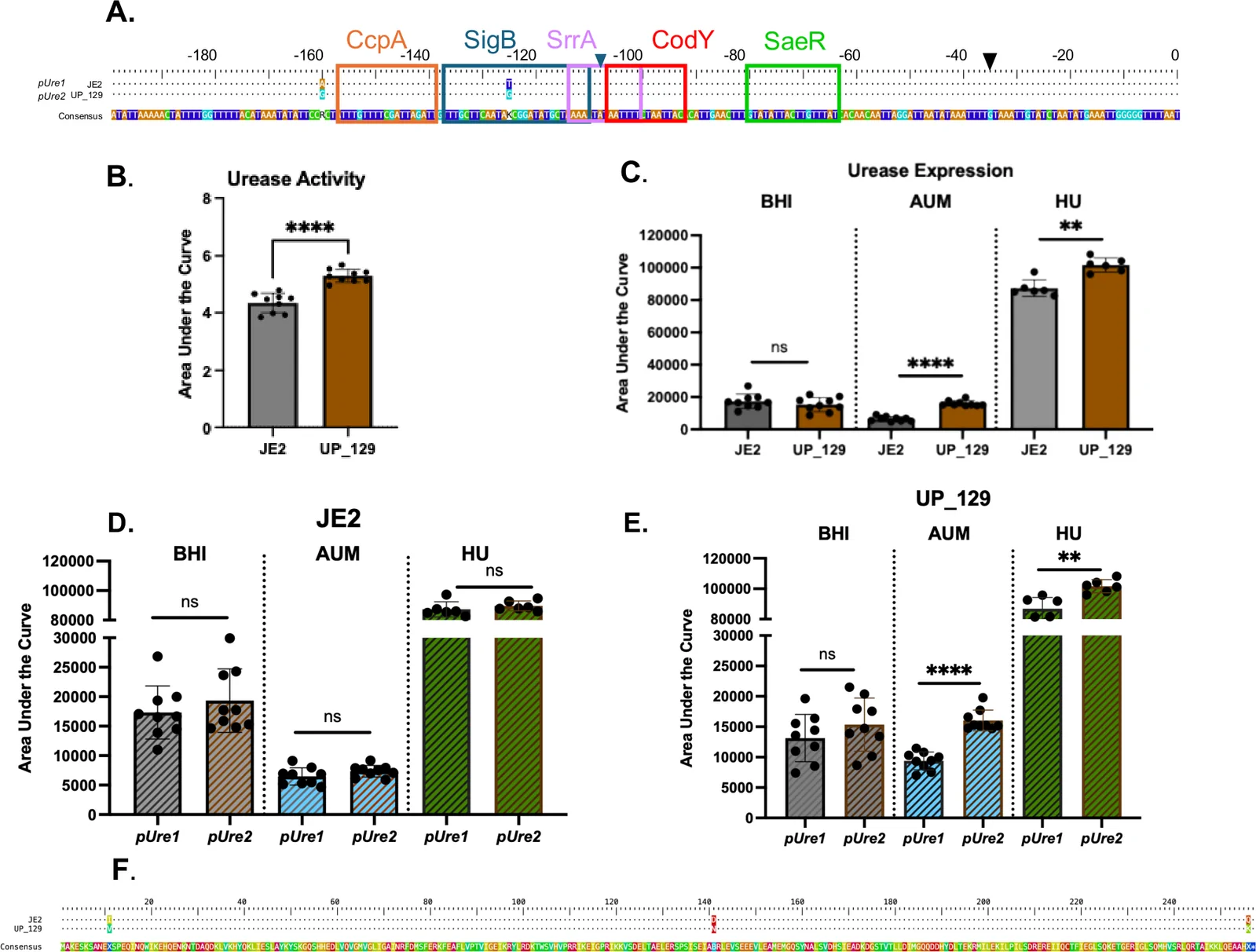
Clinically observed SNPs in the promoter alter urease. (**A**) Comparison of the JE2 and the clinical isolate UP 129 urease promoter. Dots indicate a conserved nucleotide with the consensus. Alignment was created using the DECIPHER R package (57). Predicted binding sites for transcriptional regulators CcpA, SigB, SrrA, CodY, and SaeR are denoted in boxes. Transcriptional start sites for the predicted SigA-controlled transcript (black) and the predicted SigB-controlled transcript (blue) are denoted by triangles (45). (**B**) Urease activity of JE2 and UP 129 in UB. (**C**) Urease expression of JE2 and UP 129 in BHI, AUM, and HU. Each circle represents a single replicate for JE2 (black bars) or UP 129 (brown bars). Expression was analyzed in both BHI and AUM. Fluorescence was normalized to the background strain (JE2 or UP 129) with no fluorescent reporter. Promoter swap GFP reporter assays in the JE2 (**D**) and UP 129 (**E**). Fluorescence was normalized to the background strain (JE2 or UP 129) with no fluorescent reporter in BHI (gray), AUM (blue), and HU (green). Gray bars indicate the JE2 pUre sequence (pUre1), and brown bars indicate the UP 129 pUre sequence (pUre2). (**F**) Alignment of the SigB amino acid sequence from JE2 (top) and UP 129 (bottom). Dots indicate a conserved amino acid with the consensus. Alignment was created with the DECIPHER R package (57). For each experiment, at least three biological replicates in triplicate were performed. Bars represent the mean of all replicates with the standard deviation. Area under the curve analysis was performed, and significance was determined using the Mann-Whitney *U* test.

Our previous study determined that the most common *S. aureus* lineage within the urinary tract was clonal complex (CC) 5 (12), which contrasts with JE2—a CC8 (56). Furthermore, our previous report demonstrated that longitudinal *S. aureus* urinary catheter-associated (UCA) isolates displayed increased urease activity over time (35). To begin to investigate the role of the regulatory pathway in dictating urease expression and activity in clinical isolates, we analyzed the sequence conservation of the urease promoter among >170 *S*. *aureus* UCA isolates (File S3). Of the UCA sequences analyzed, the urease promoter was highly conserved among all the isolates. Clustering at 100% nucleotide identity revealed 11 unique sequence groups (Fig. S7A). Strikingly, most isolates belonged to just two groups, comprising 57 (33%) and 49 (28%) isolates, respectively. However, the JE2 promoter was not represented within either of these two groups and instead grouped with only a single clinical isolate (Fig. S7A). The most common promoter sequence of the UCA isolates (pUre2) differed by only two single nucleotide polymorphisms (SNPs) compared to the JE2 promoter sequence (pUre1) (Fig. 5A; Fig. S7A). Notably, one SNP was in the predicted SigB consensus binding site (Fig. 5A). This finding suggested that SNPs in the urease promoter may impact urease expression and activity in the catheterized bladder.

### SNPs in the promoter affect urease expression

To determine the importance of the SNPs within the most common urease promoter observed in the UCA isolates, the representative isolate UP 129 was selected for further characterization. UP 129 is not only a CC5 strain (30), but it also contains the most common urease promoter sequence (Fig. 5A; Fig. S7A). Impactfully, UP 129 also displays significantly higher urease activity compared to JE2 (Fig. 5B; Fig. S7B). To determine if urease expression differed between the two strains, a promoter fusion using the GFP reporter plasmid (pUre2) was generated in UP 129 (Fig. S7C; File S2), and urease expression was assessed in BHI, AUM, and HU over a 12 h time course (Fig. S7D through F). In BHI, there was no difference in the expression of urease between JE2 pUre1 and UP 129 pUre2 (Fig. 5C; Fig. S7D). However, in AUM and HU, UP 129 pUre2 displayed significantly higher urease expression compared to JE2 pUre1 (Fig. 5C; Fig. S7E and F). Next, to determine the impact of the SNPs within the most common promoter on expression, pUre1 and pUre2 promoter swaps were generated in the opposite strain backgrounds. There was no significant difference between the expression of either pUre1 or pUre2 in the JE2 background in BHI, AUM, or HU (Fig. 5D; Fig. S7G). Similarly, there was no difference in the expression of pUre1 and in the UP 129 background in BHI (Fig. 5E; Fig. S7H). In contrast, in AUM and HU, pUre2 resulted in significantly higher urease expression compared to pUre1 in the UP 129 background. These results suggest that in UP 129, the native promoter (pUre2) is more responsive to the urinary tract-like environment compared to the JE2 promoter (pUre1). The differences in expression of the pUre2 promoter between the JE2 and UP 129 backgrounds led us to compare the genomic conservation of the urease operon and the regulators between these two isolates. The protein sequences for *ureA*, *ureC*, *ureD*, *ureE*, and *ureG* were completely conserved between JE2 and UP 129, while the sequences of *ureB* and *ureF* differed by only one amino acid each, indicating a high level of conservation of the 7 kb urease operon (Fig. S8A). Next, analyses of the amino acid sequences of the urease regulators, CodY, CcpA, AgrA, and SrrA, determined that the protein sequences are completely conserved between JE2 and UP 129 (Fig. S8B). However, the amino acid sequence of SigB differed in three different locations—amino acids 11, 141, and 246 (Fig. 5F). These amino acid changes, in combination with the SNPs in the pUre2 sequence, may be synergistic and result in the observed increase in urease expression and activity in the UP 129 strain. Impactfully, these data demonstrate that as few as two SNPs within the promoter region of urease can have significant impacts on expression.

### Urease contributes to biofilm formation under conditions that mimic the catheterized bladder

**Fig 6:**
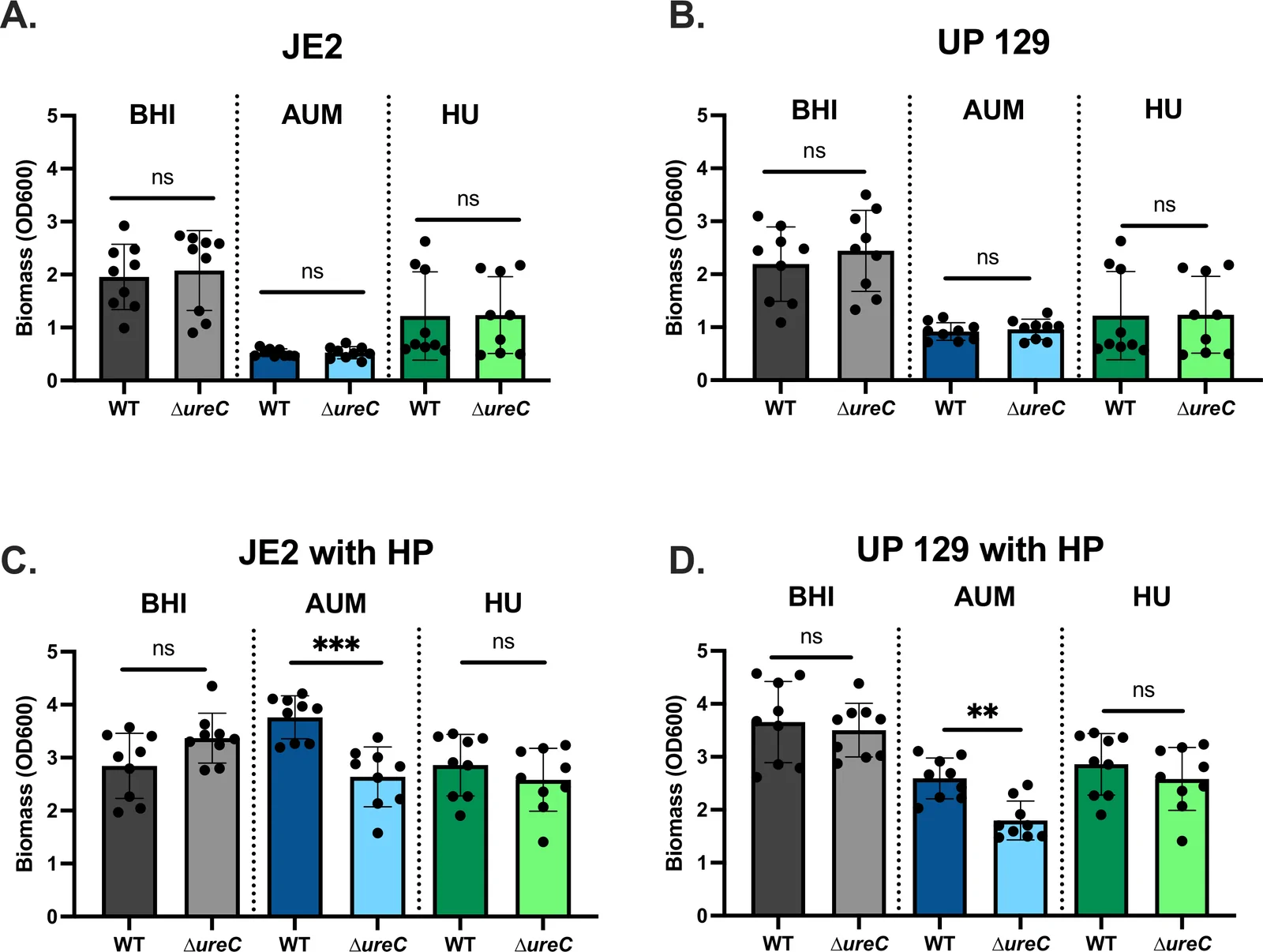
Crystal violet biofilms of WT and ∆*ureC* strains. The biofilm biomass was measured under *in vitro* conditions in the JE2 wild-type strain and the JE2 ∆*ureC* strain (**A**) and the UP 129 wild-type strain and the UP 129 ∆*ureC* strain (**B**). To mimic a host-like environment and enhance biofilm formation, the biofilm assays were repeated in the presence of HP in both the JE2 (**C**) and the UP 129 (**D**) background. All assays were performed in BHI (gray bars), AUM (blue bars), and HU (green bars). At least three biological replicates in technical triplicate were performed. Bars represent the mean of all replicates with the standard deviation. The Mann-Whitney *U* test was used to determine statistical significance, where *** is *P* < 0.0005, ** is *P* < 0.005, * is *P* < 0.05, and *P* > 0.05 is no significance (ns).

Previous studies indicate that the genes in the urease operon are upregulated in biofilms (58). Thus, to determine the role of urease in biofilm formation, isogenic *ureC* mutants were generated in JE2 and UP 129 (Fig. S9A), and biomass in BHI, AUM, and HU was measured. Consistent with previous reports (24), there was no difference in biofilm formation in either BHI, AUM, or HU between either of the parent or *ureC* mutant strains (Fig. 6A and B). However, since it is well established that the healthy urinary tract differs from the catheterized urinary tract environment, as additional host proteins, including fibrinogen and serum albumin, are recruited and accumulate during catheterization, biofilms were also formed in the presence of human plasma (HP) (27, 59), which mimics the presence of these host factors (26). As expected, the presence of HP significantly enhanced biofilm formation in all conditions (*P* value less than or equal to 0.0188). Importantly, both JE2∆*ureC* and UP 129∆*ureC* formed significantly less biomass compared to their respective parent strains in AUM, but not in BHI or HU (Fig. 6C and D). The high variability of biofilm growth in HU was not surprising, as there is high variance in metabolites found in human urine samples, which can affect biofilm formation (60). These results suggest that urease promotes *S. aureus* biofilm under conditions that mimic the catheterized bladder.

### Urease promotes dissemination during CAUTI

**Fig 7:**
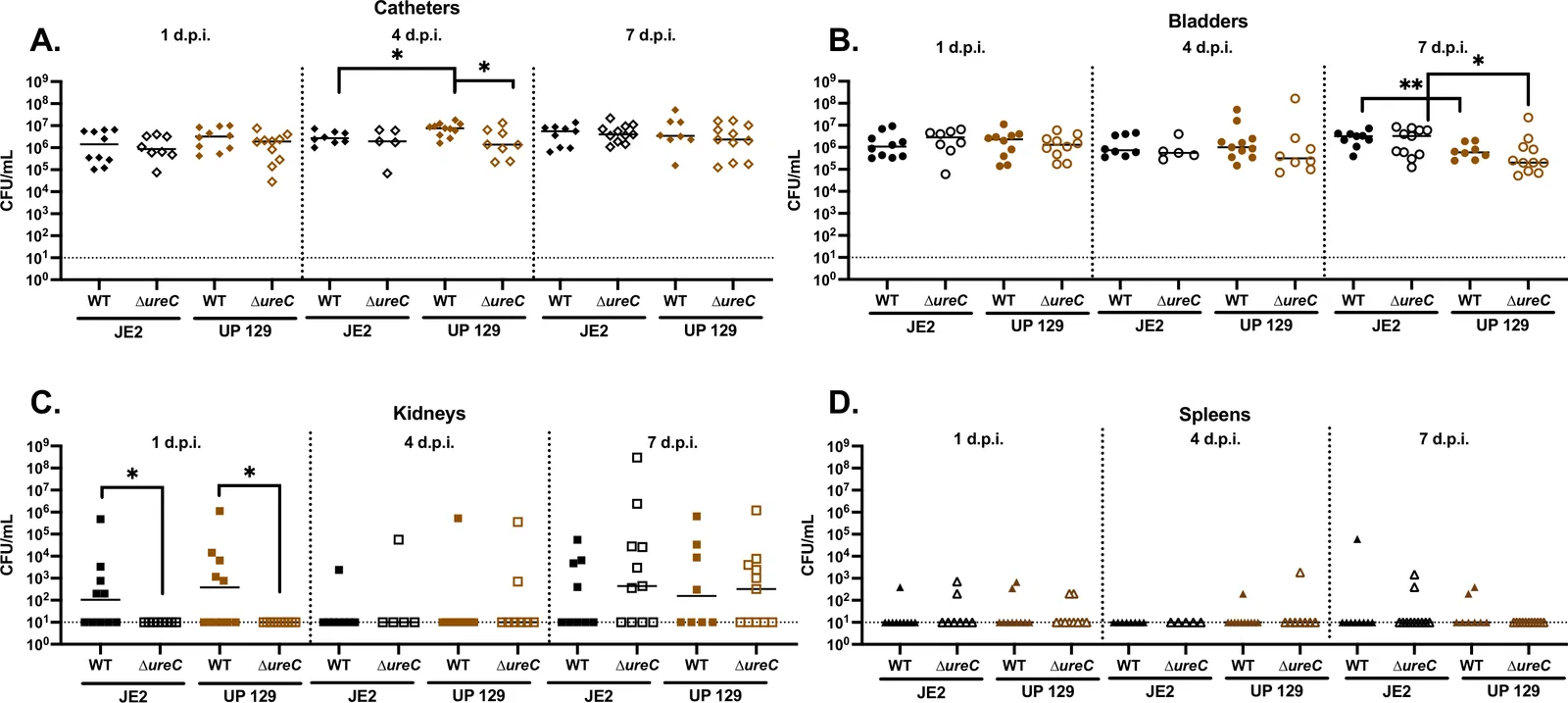
CAUTI mouse model of *S. aureus* wild-type and ∆*ureC* strains. Mice were infected with either JE2, JE2∆*ureC*, UP 129, or UP 129∆*ureC* for 1 and 4 dpi. Colony-forming units (CFUs) were obtained from the catheter (**A**), bladder (**B**), kidneys (**C**), and spleen (**D**). Each symbol represents one catheter, bladder, kidney, and spleen from an individual mouse, respectively. Closed black symbols indicate mice infected with JE2, and open black symbols indicate mice infected with JE2∆*ureC*. Closed brown symbols indicate mice infected with UP 129, and open brown symbols indicate mice infected with UP 129∆*ureC*. The Mann-Whitney *U* test was used to determine statistical significance between groups, where *** is *P* < 0.0005, ** is *P* < 0.005, * is *P* < 0.05, and *P* > 0.05 is no significance (ns). Solid lines indicate the median.

To investigate potential strain differences between JE2 and UP 129, we assessed bladders, catheters, kidneys, and spleens at 1, 4, and 7 days post-infection (dpi) for bacterial load, as previously described (26, 61, 62) (Fig. 7). Notably, at most time points, there were no significant differences in bacterial burden between the two strains. However, at 4 dpi, UP 129 displayed significantly higher catheter colonization compared to JE2 (Fig. 7A). These data suggest that UP 129 may have an advantage in colonizing surfaces, such as the urinary catheter.

Next, to determine the contribution of urease to CAUTI, the *ureC* mutant strains were assessed at 1, 4, and 7 dpi in both JE2 and UP 129 (Fig. 7). At most time points, there was no significant difference in organ colonization between either wild-type strain or their respective *ureC* mutant (Fig. 7 and Fig. S9). However, UP 129∆*ureC* displayed significantly lower catheter colonization compared to UP 129 at 4 dpi, which contrasted with JE2 (Fig. 7A). This difference may be due to colonization differences on the surface of the catheter between the JE2 and UP 129 parent strains (Fig. 7A). Impactfully, at 1 dpi, the ∆*ureC* of both background strains did not disseminate to the kidney, resulting in significantly lower bacterial loads compared to their respective parent strains (Fig. 7C). This phenotype was ablated at 4 and 7 dpi as the mice began to eliminate the disseminated infection with the wild-type strains (Fig. 7C). Impactfully, our mouse model demonstrates that urease is essential for dissemination to the kidney during the acute stage of infection (1 dpi). Together, these results suggest that urease plays a role in promoting initial spread from the bladder to the kidneys in CAUTI.

### Urease modulates the inflammatory response during *S. aureus* CAUTI

**Fig 8:**
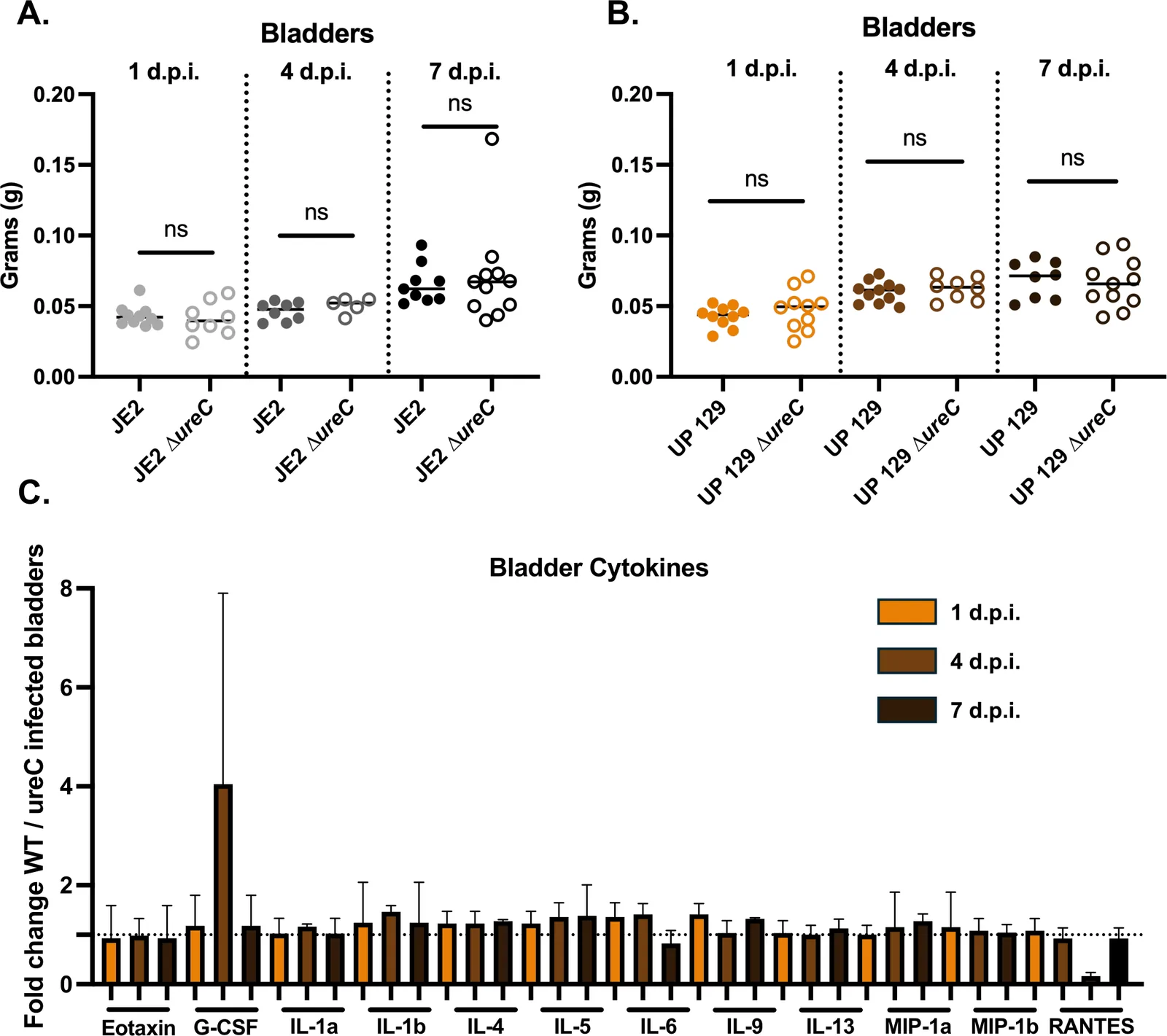
Inflammatory response of mice infected with UP 129 and UP 129∆*ureC*. Bladders from mice infected with JE2 and JE2∆*ureC* (**A**), or UP 129 and UP 129∆*ureC* (**B**) were weighed prior to processing for CFU enumeration. Each dot represents a single bladder. Solid dots indicate mice infected with the wild-type strains, and open dots indicate mice infected with the ∆*ureC* strains. (**C**) Cytokine levels in the UP 129∆*ureC* strains were normalized to cytokines in the UP 129 wild-type strain. Bladders from mice 1, 4, and 7 dpi were split into two groups and pooled. Each pool was assessed in duplicate and analyzed for levels of 23 inflammatory cytokines. The 12 cytokines with the highest fold changes are graphed. The Mann-Whitney *U* test was used to determine statistical significance between groups, where *** is *P* < 0.0005, ** is *P* < 0.005, * is *P* < 0.05, and *P* > 0.05 is no significance. Solid lines indicate the median.

To determine the impact of urease on inflammation during CAUTI, we measured the weights of bladders, as a proxy for edema, as previously described (26, 62). At 1, 4, and 7 dpi, there was no significant difference between the weights of bladders in mice infected with wild-type strains compared to their *ureC* knockout strains (Fig. 8A and B). This phenotype was observed in both the JE2 and UP 129 strain backgrounds, suggesting that this is not a strain-specific phenotype. Next, we analyzed the expression of various cytokines in UP 129 and UP 129∆*ureC* infected bladders (Fig. 8C). Most cytokines were expressed at similar levels in UP 129 compared to UP 129∆*ureC* infected bladders. However, at 4 dpi, a fourfold increase in granulocyte colony-stimulating factor (G-CSF) and a near sevenfold decrease in RANTES expression were observed in bladders infected with UP 129 compared to UP 129∆*ureC* (Fig. 8C). G-CSF (63) is generally recognized for its role in inflammation, which is required for granulocyte and macrophage differentiation and neutrophil recruitment (63). However, G-CSF can also act as an anti-inflammatory regulator. RANTES is a pro-inflammatory cytokine that commonly acts as a chemoattractant for multiple types of immune cells (64). Notably, these results correspond with the higher catheter burdens at 4 dpi in the UP 129 compared to the UP 129∆*ureC* infected mice. These inflammatory phenotypes may be due to the significantly higher catheter burdens in UP 129 infected mice. Alternatively, urease may modulate the inflammatory response to promote *S. aureus* CAUTI.

## DISCUSSION

CAUTIs are the most common hospital-associated infection in the United States, and incidences of infection are increasing (8, 9, 11). As the global population ages, many individuals will enter nursing homes, where the urinary catheter prevalence is higher than the general population (up to 7.3%) (65, 66). Despite *S. aureus*’s prevalence in the catheterized bladder and the severity following symptomatic infection, the uropathogen has been largely understudied. While *S. aureus* encodes numerous well-studied virulence determinants, factors specifically important for CAUTI remain largely understudied (31). Our previous data show that among UCA isolates, temporal increases in urease activity are detected, implicating this virulence factor as a contributor to CAUTI (35). While some mechanistic studies have investigated *S. aureus* urease in the healthy urinary tract (24, 67, 68), there have been no studies of how urease contributes to *S. aureus* CAUTI.

**Fig 9:**
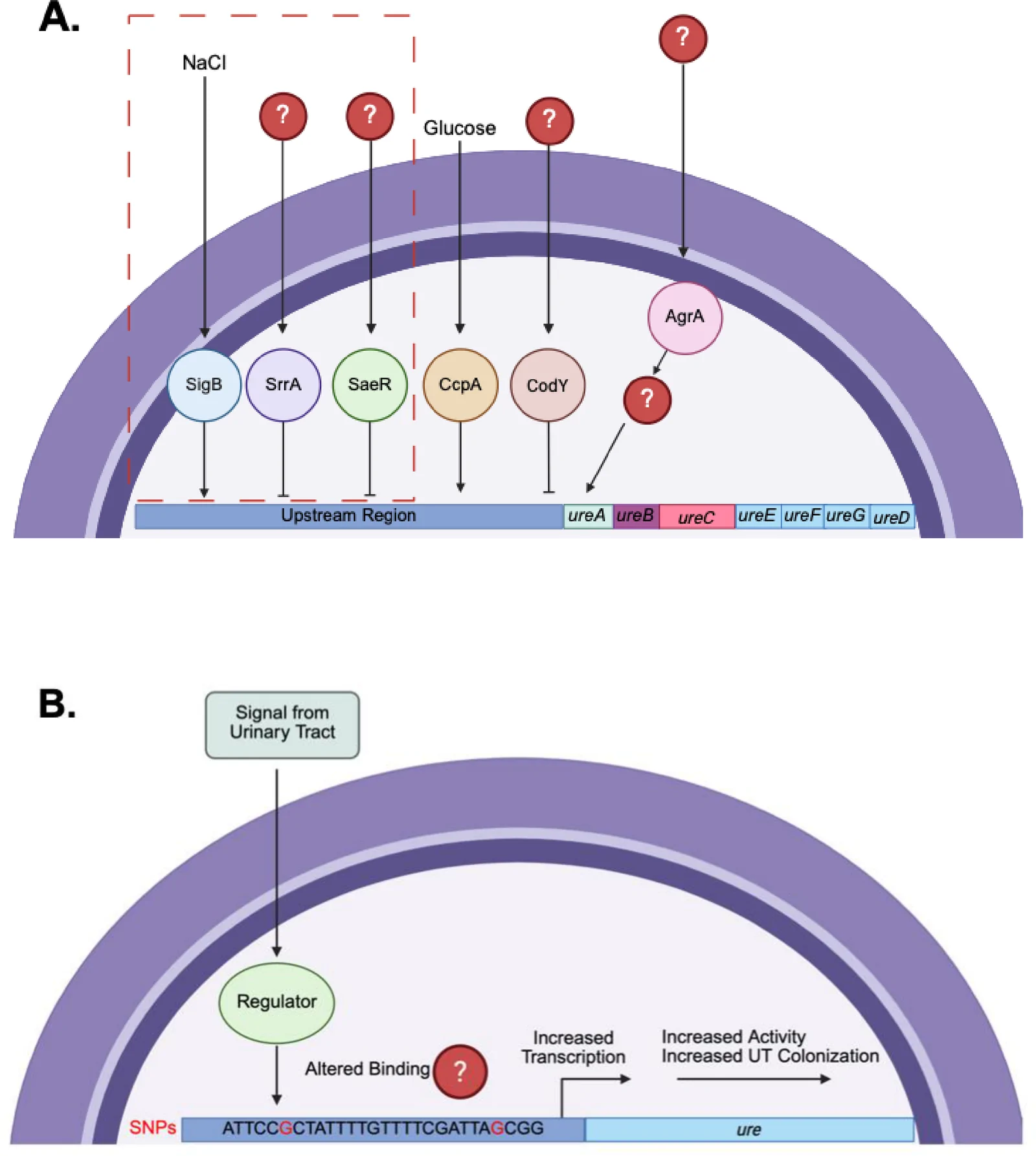
Model of urease regulation in *S. aureus*. (**A**) Previous work has shown that CodY is a repressor of urease, while CcpA and Agr are activators of urease (25, 28). Here, we show that SigB, SrrA, and SaeR are novel activators and repressors of urease expression and activity, respectively (red box). CcpA, SigB, SrrA, SaeR, and CodY are predicted to either promote or inhibit urease expression by binding to the upstream region of the urease operon to either recruit or block RNA polymerase II from transcribing the operon. AgrA is predicted to act through an unknown regulator to promote urease expression. (**B**) SNPs observed in the most common promoter region among clinically collected UCA isolates. SNPs in the upstream region are sufficient to increase urease transcription, which in turn is expected to increase urease activity, and ultimately, support colonization of *S. aureus* in the urinary tract. SNPs are predicted to influence urease expression by altering the binding of urease regulators to the promoter. Created in BioRender (J. Gomez, 2026, https://BioRender.com/7r28v0m).

In this study, we show that a complex regulatory network controls urease expression and activity in *S. aureus* (Fig. 9A). This complex network of regulation may explain why *S. aureus* urease is difficult to detect in a clinical setting, as the standard Christensen urea agar test only detects activity in 25%–50% of clinical *S. aureus* isolates, despite reports that >90% of *S. aureus* strains encode the urease operon (36–38). Here, we show that growth phase and environmental conditions that mimic the urinary tract impact urease expression. Specifically, we have shown that post-exponential growth is a potent and consistent inducer of *S. aureus* urease. This phenotype may explain why urease is hard to detect in *S. aureus*, as the Christensen urea agar tests are performed with exponential phase cultures. Additionally, compared to standard lab media (BHI), media that mimic the urinary tract (AUM, HU, and UB) significantly increase urease expression and activity. Notably, the phenotypes displayed following growth in AUM often mimicked the phenotypes observed in HU yet were more consistent. The inconsistency observed for the phenotypes in HU may be due to the high variability of metabolites both within and between individuals (60, 69). The urinary tract is a nutrient-limited environment, and urine composition vastly differs between individuals (69). Over 3,000 metabolites have been identified in HU (60, 69), indicating the diversity of signals and nutrients that microbes living in the urinary tract respond to and use for survival. Together, these findings highlight why *S. aureus* is likely labeled as a weak urease producer in the clinic yet is second only to *P. mirabilis* in causing urease-induced catheter encrustations (13, 20).

Previous studies investigating the regulation of *S. aureus* urease show that CodY, CcpA, and Agr control urease expression (25, 28). In this study, we confirmed these findings and show that three additional regulators—SigB, SrrA, and SaeR—also control urease (Fig. 9A). Additionally, we show a lack of correlation between the ability of these mutants to grow and their effects on urease expression and activity. For example, despite its ability to grow significantly better than JE2 in both BHI and AUM, the *sigB* mutant displayed little to no urease expression and activity. Impactfully, the findings presented here may also explain why urease expression and activity are highest during the post-exponential phase. CodY plays an essential role during stationary phase to relieve repression of metabolism-related genes (33, 42, 55, 70). Similarly, CcpA coordinates catabolism to allow the preferential metabolization of carbon sources (28, 71, 72). Thus, as the limited nutrients within the urinary tract, such as glucose or BCAAs, are metabolized, CodY and CcpA may allow the depression or induce the expression of specific genes within their respective regulons, including urease.

Many of the signals that are sensed by these regulators are present in HU (60, 69) and are known to influence *S. aureus* regulation (28, 47–51). Here, we show that signals present in HU, including glucose and NaCl, influence urease expression and do so via their corresponding regulator (Fig. 9A). Furthermore, we show that while other regulator signals, such as BCAAs or low oxygen conditions, affect urease expression, these phenotypes were independent of their cognate regulator, suggesting alternative regulation pathways are contributing to urease expression in response to these signals. There is substantial crosstalk between the CodY, CcpA, SigB, Agr, and SrrA regulators (31, 52, 55, 71–74), which complicates the regulation of urease (23, 61–66). Specifically, CodY, CcpA, SigB, and SrrA have all been shown to regulate the expression of the Agr quorum sensing system (31, 52, 71, 72, 74). As such, any perturbation of these regulons may also impact Agr and subsequently urease. Furthermore, recent evidence indicates that SrrAB is not the only regulator that is essential for nitrous oxide resistance, as CodY is also essential for this response specifically during stationary phase (73). Thus, crosstalk may contribute to the inverse effects of SrrA on urease expression during the exponential and post-exponential phases of growth. These complexities necessitate further mechanistic studies to determine the exact regulatory pathways that are directly and indirectly impacting urease expression and activity (73).

In previous work, we found that *S. aureus* strains that persist asymptomatically in the urinary tract display temporal increases in urease activity, suggesting some strains may encode genomic changes in the urease pathway that promote expression of the enzyme (12, 35). Here, we show that the most common promoter among the clinical isolates differed by two SNPs compared to the JE2 promoter. Furthermore, these two SNPs are sufficient for altering urease expression in the representative clinical isolate UP 129 (Fig. 9B). Interestingly, there was no difference between expression of the two promoter sequences in JE2. This suggests that despite conservation across most of the urease operon and regulon, additional genomic changes, such as the missense mutations in SigB, also likely contribute to urease regulation among clinical isolates. However, CC5 and CC8 are different lineages and encode diverse accessory genomes. Therefore, multiple other factors, such as other proteins in the urease regulator pathways or changes in nickel acquisition, may alter urease expression or activity. Future studies using SigB allelic swaps in the JE2 and UP 129 strains will provide additional support for this hypothesis.

One important facet of *S. aureus* CAUTI is biofilm formation, and a previous study suggested that the urease operon is upregulated in biofilms (58). Here, we show that our initial findings aligned with previous work demonstrating that urease is not essential for biofilm formation in rich media (24). However, many studies analyzing the surface of human catheters have shown that *S. aureus* forms complex biofilm structures with host proteins on the catheter surface (10, 13, 15, 18, 27, 75). By using growth conditions that more accurately reflect the catheterized bladder (10, 26, 27, 76–78), we demonstrate that urease promotes biofilm, regardless of strain background. Furthermore, these results emphasize the necessity of studying *S. aureus* urease under conditions that accurately represent host environments, as urease promotes *S. aureus* biofilm only under conditions that mimic the catheterized urinary tract.

In contrast to previous work in a non-catheterized UTI model, which showed that urease does not play a role in *S. aureus* UTI (24), this study demonstrates that urease is important for *S. aureus* CAUTI (26). Specifically, urease is critical for dissemination to the kidney at 1 dpi across strain backgrounds. Furthermore, UP 129, a high urease-producing strain, displayed significantly higher catheter colonization at 4 dpi compared to JE2, a lower urease-producing strain. This phenotype corresponded with a significantly lower catheter burden in UP 129∆*ureC* compared to UP 129-infected mice at 4 dpi. Impactfully, these findings correlate with our biofilm data in the catheterized-like urinary tract environment. Our results contrast with previous work in *S. aureus* UTI, likely because of the substantial differences within the urinary tract following catheterization (24, 26, 27, 59, 76). Specifically, it is well established that catheterization promotes persistent, robust *S. aureus* colonization in the urinary tract by initiating the release of host factors that are exploited by *S. aureus* adhesins to promote colonization (26). These host-pathogen interactions are not present in the healthy, non-catheterized bladder, resulting in clearance of *S. aureus* from the urinary tract within a few days (26, 27). Thus, our results highlight the importance of urease in forming biofilms in the catheterized bladder environment and emphasize the importance of using models that mimic disease. Impactfully, the inability of the urease mutant to spread from the bladder at 1 dpi indicates that interventions that target the enzyme may be effective at reducing the risk of severe secondary infections developing in individuals who are colonized with *S. aureus* long term, as bacteria that reach the kidneys can cause pyelonephritis, leading to kidney damage, or can allow direct access to the bloodstream. This finding may explain why *S. aureus* is associated with an increased risk of secondary bacteremia during CAUTI (18, 19).

Overall, our findings not only demonstrate that environmental conditions play a crucial role in regulating urease, but also that strain differences can contribute to differences in expression and activity. Specifically, among clinical UCA isolates that are adapted to the catheterized bladder environment, urease expression and activity are higher when compared to a historical strain. This study emphasizes the importance of using clinical isolates in understanding pathogenesis, as the phenotypes can differ between these isolates and historical strains. Future work will aim to further elucidate the mechanism of *S. aureus* urease regulators and will aim to determine which genomic changes have the greatest impact on urease expression and activity phenotypes. Deeper insight into the mechanisms that promote adaptation to the bladder may support therapeutic discoveries that target *S. aureus* CAUTI, which will ultimately improve the quality of life of individuals who are catheterized long-term.

## MATERIALS AND METHODS

### Bacterial strains and growth conditions

All strains used in this study are listed in Table 2. JE2 and all transposon mutants used in this study were obtained from BEI Resources, and the *S. aureus* clinical isolate UP 129 was collected previously from an individual with long-term indwelling urinary catheters (11, 12). Brain heart infusion (BHI) broth (Becton Dickinson and Company, REF #237500) and agar (Fisher, CAS #9002-18-0) were used to grow and maintain all *S. aureus* cultures for experiments. Transposon mutants were selected for and maintained with BHI or tryptic soy broth (TSB) (Sigma-Aldrich, REF #22092-500G) supplemented with 50 ug/mL of erythromycin (Acros Organics, CAS #114-07-8). The pJB38∆*ureC* plasmid was maintained in *Escherichia coli* DH5α in LB broth (Fisher, REF #BP1426-500) supplemented with 50 ug/mL of ampicillin (Fisher, CAS #69-52-3) at 37°C and maintained in *S. aureus* in BHI supplemented with 50 ug/mL of chloramphenicol (Sigma-Aldrich, REF #C0378-25G) at 30°C. Once homologous recombination was complete, the ∆*ureC* strains were maintained in BHI with no antibiotic. pUre plasmids were generated in *E. coli* DH5α in LB supplemented with 50 ug/mL of ampicillin and maintained in *S. aureus* in BHI supplemented with 50 µg/mL of erythromycin and/or chloramphenicol. Urease broth was made as previously described (35). Briefly, 1 g of peptone (BD Biosciences, REF #211921), 1 g glucose (Sigma-Aldrich, REF #G8270-1KG), 2 g potassium phosphate monobasic (Sigma-Aldrich, REF #P9791-100G), 5 g sodium chloride (Fisher, CAS #7647-14-5), and 0.012 g phenol red (Sigma-Aldrich, REF #114529-5G) were resuspended in 1 L of water. Media were sterilized and supplemented with filter-sterilized 40% urea solution (Fisher, REF #BP169-500). AUM was adapted based on previously published artificial urine recipes. Briefly, AUM was prepared as in Table 1 and previously described (69), with the exception that an additional 0.909 mM tryptone (Becton Dickinson and Company, REF #211705), 0.833 mM glucose (Fisher, CAS #50-99-7), 82 nM biotin (Thermo, Cat #230090010), and 0.17% yeast nitrogen base (YNB) without amino acids and ammonium sulfate (Becton Dickinson and Company, REF #233520) was added. To adjust the buffering capacity changes associated with the addition of YNB, the amount of sodium phosphate monobasic (Mallinckrodt Pharmaceuticals, CAS #10049-21-5) was adjusted to a total of 18.667 mM. The final pH of the AUM is around 6, which is in the range of human urine for healthy adults (79).

**TABLE 2:** Strains used in this study

Denotation in this paper	Isolation site	Reference	Denotation in reference
JE2	Skin	(80)	JE2
JE2 ∆*ureC*	Skin	This study	JE2 ∆*ureC*
JE2 *codY::tn*	Skin	(81)	JE2 *codY::tn*
JE2 *ccpA::tn*	Skin	(81)	JE2 *ccpA::tn*
JE2 *sigB::tn*	Skin	(81)	JE2 *sigB::tn*
JE2 *agrB::tn*	Skin	(81)	JE2 *agrB::tn*
JE2 *saeR::tn*	Skin	(81)	JE2 *saeR::tn*
JE2 *srrA::tn*	Skin	(81)	JE2 *srrA::tn*
RN4220	Cloning strain	(82)	RN4220
UP 129	Urinary catheter	(11)	Urology outpatient 129
UP 129 ∆*ureC*	Urinary catheter	This study	UP 129 ∆*ureC*

### Generation of chloramphenicol-resistant GFP transcriptional fusion plasmid

GFP transcriptional fusion plasmids compatible with the transposon mutant strains (erythromycin cassette) were generated by inserting a chloramphenicol resistance cassette into the pCM11 plasmid, as previously described (83). Briefly, the pGFP chloro F and pGFP chloro R primers flanked with XbaI and PstI restriction sites (Table 3) were used to amplify the chloramphenicol cassette from the pJB38 plasmid (84). The PCR product was cloned directly into the pCR4-Blunt TOPO plasmid using the Zero Blunt TOPO PCR Cloning Kit (Thermo Fisher, Cat #450031) according to the manufacturer’s protocol. The plasmid was then transformed into *E. coli* DH5α. The pCR4-Blunt TOPO-chloramphenicol cassette plasmid and the pCM11 plasmid were then digested using the XbaI and PstI restriction enzymes. Both fragments were gel purified and ligated with T4 DNA ligase. The resulting plasmid (pGFPchloro/erm) was then transformed into DH5α and was confirmed via sequencing (File S2). The pGFPchloro/erm plasmid was then used as a backbone for GFP transcriptional fusions of the upstream region of the urease operon.

**TABLE 3:** Primers used in this study^*a*^

Primer name	Sequence	Citation
pGFP chloro F	tctagaCTTTATTCTTCAACTAAAGCAC	This study
pGFP chloro R	ctgcagCATATTATAAAAGCCAGTC	This study
Upstream urease F	aagcttTCTATGAAAAGTTATATTAAAAAC	This study
Upstream urease R	ggtaccATTAAAACCCCCAATTTCA	This study
pGFP check F	GTCACGACGTTGTAAAACGA	This study
pGFP check R	CGTTTCATGTGATCCGGA	This study
ureC KO upstream F	ccgaattcGATTACAGATATCGAAATCGAGGCTA	(35)
ureC KO downstream R	ccgtcgacCCTGTTGGAAACTGTGAATCACAG	(35)
CcpA check F	GTTATATTAAAAACTATTTTGG	This study
CcpA check R	CCCAATTTCATATTAGATAC	This study
SigB check F	GAATCTTTAATGGATGAAGTCACAG	This study
SigB check R	CTCGATTTCAATTTAATAATCAAGG	This study
SrrA check F	GATGAGGATAGAATCAGAAG	This study
SrrA check R	TAATGCCAAACTTCTTTTAAT	This study
CodY check F	GTATTTATTGTATCGCGTCGAGG	This study
CodY check R	GATAACATCGTCACGACTAGGAC	This study
AgrB check F	GATAATAAAATTGACCAGTTTGCCAC	This study
AgrB check R	GAATGAATTGGGCAAATGG	This study
SaeR check F	ATGACCCACTTACTGATCG	This study
SaeR check R	TTATCGGCTCCTTTCAAA	This study
Upstream	CTCGATTCTATTAACAAGGG	(81)
Buster	GCTTTTTCTAAATGTTTTTTAAGTAAATCAAGTAC	(81)

^*a*^ Uppercase letters indicate bases that are homologous to the corresponding plasmid or gene. Lowercase letters indicate restriction enzyme sites.

### Generation of urease transcriptional GFP fusion constructs

GFP transcriptional fusions to the upstream region of the urease operon were generated using the pGFPchloro/erm plasmid, as previously described (85). Briefly, the upstream region of urease (200 bp) was amplified using the forward (upstream urease F) and reverse (upstream urease R) primers flanked with HindIII and KpnI restriction sites (Table 3). The PCR product was cloned directly into the pCR4-Blunt TOPO plasmid, using the Zero Blunt TOPO PCR Cloning Kit and transformed into DH5α. The plasmid was then isolated and digested using HindIII and KpnI restriction enzymes. Following gel purification, the upstream region fragment was ligated into pGFPchloro/erm, which was similarly digested with HindIII and KpnI. The resulting plasmids (pUre) were confirmed via PCR and sequencing (File S2). pUre plasmids were then transformed into the *S. aureus* lab strain RN4220 and subsequently transduced via phage 11 into the JE2, as previously described. The pUre plasmids were introduced into the UP 129 strain via electroporation, as previously described (35). Final plasmids were confirmed via PCR (Fig. S8A).

### GFP transcriptional fusion expression assays

Transcriptional fusion expression assays were performed based on previous work (35, 85). Overnight cultures were grown at 37°C in BHI. For analysis of exponential phase cultures, strains were diluted 1:100 from overnight culture in various growth media, including BHI, AUM, pooled female HU (see “Human urine collection” below), a 1:1 ratio of pooled female HU and saline, and urease broth (35) without phenol red. For analysis of post-exponential phase cultures, 1 mL of culture was washed in 1× phosphate-buffered saline (PBS) and then resuspended in various growth media. Cultures were transferred to a clear-bottom, black-walled 96-well plate, and OD_600_ and fluorescence were measured every hour for a total of 18 h in the plate reader. GFP excitation wavelength was 488 nm, and the emission was collected at 515 nm, as previously described (86). Fluorescence of each strain was normalized by subtracting the fluorescence from the background strain (no GFP reporter plasmid) from each strain that contained the GFP reporter plasmid.

### Human urine collection

Human urine was sourced from the Urine BioBank for Urinary Tract Infection Studies. Briefly, donors were consented and enrolled prior to donation. Urine from at least two healthy female donors, aged 18+ were collected and pooled. Donors were not taking antibiotics nor currently menstruating, pregnant, or experiencing symptoms of a UTI. Urine was sterilized with a 0.22 um filter (Thermo, Cat #595-3320) and stored at 4°C until use. A one-to-one ratio of HU and saline (HU:S) solution was created by combining one volume of HU with one volume of 0.9% saline solution. The average sample of human urine collected ranged from pH 6.5 to 7.0.

### Identification of consensus regulator binding sites

Consensus regulator binding sites within the regulatory region of the urease operon were identified using the program Artemis (Sanger, version 18.1.0) to analyze the 200-base pair sequence upstream of the urease operon. A total of 172 *S. aureus* isolates were previously isolated from long-term catheterized individuals, and their genomes were sequenced (File S3) (9, 11, 12). The sequence of the *S. aureus* historic strain JE2 was obtained from NCBI (File S3). The urease operon and upstream region were identified in each genome, and consensus binding sequences of predicted regulators were identified based on the reported binding consensus sequences in the literature (39–44). Alignment of the 200-base-pair region upstream of the urease operon for the 172 UCA isolates (11, 12) and JE2 was created using the DECIPHER software package (57) in RStudio (Posit Software, version 2023.06.0+421).

### Transposon mutant generation

Mutations in predicted urease regulators were reconstructed in the JE2 wild-type strain background using the Nebraska Transposon Mutant Library (NTML), as previously described (35). Briefly, phage transduction using phage 11 was used to generate lysates of each transposon mutant regulator. The resulting phage lysate was used to backcross each transposon mutant into the JE2 strain background and was selected for using TSA supplemented with 20 mM sodium citrate (CAS #6132-04-3) and 50 ug/mL erythromycin. Transposon mutants were confirmed via PCR (Fig. S3A) using the forward and reverse regulator primers and the upstream or Buster primers listed in Table 3.

### Generation of *ureC* clean deletion strains

The ∆*ureC* strains were generated as previously described (35). Briefly, the previously generated pJB38∆*ureC* plasmid (35) was isolated using a plasmid miniprep kit (Promega, REF #A1330) following the manufacturer’s protocol, with the exception that 10 ug/mL of lysostaphin was used to lyse *S. aureus* cells (Sigma-Aldrich, REF #L7386-5MG). Electrocompetent cells of JE2 and UP 129 were generated as previously described (35). Briefly, overnight cultures were subcultured into 50 mL of B2 medium (1% casein hydrolysate [Oxoid, REF #LP0041], 2.5% yeast extract [Fisher, REF #BP1422-500], 0.5% glucose [Sigma-Aldrich, REF #G8270-1KG], 2.5% NaCl [Fisher, REF #S671-500], 0.1% K_2_HPO_4_ [Sigma-Aldrich, REF #P8281-100G]) at an OD_600_ of 0.25. Cells were grown at 37°C for ~2 h until OD_600_ 0.4. Cells were then washed in 50 and 12.5 mL of cold, sterile water, followed by 7 mL of cold 10% glycerol. Finally, cells were resuspended in 200 uL of cold 10% glycerol and stored at −80°C until use. Electrocompetent cells were transformed with purified pJB38∆*ureC* plasmid, and transformants were selected for at 30°C on chloramphenicol plates. Co-integrates were selected for at 42°C overnight on chloramphenicol plates, and resulting colonies were grown in TSB without antibiotic at 30°C. Recombinants were counter-selected with 100 ng/mL of anhydrotetracycline hydrochloride (Fisher, Cat #AAJ66688MA). Colonies were screened for both urease activity and chloramphenicol sensitivity. The deletion of the *ureC* gene was further confirmed via PCR (Fig. S9).

### Urease activity assays

Urease activity was measured as previously described (35). Briefly, bacterial strains were grown in BHI for 18 h. Cultures were washed in 1× PBS and resuspended in urease broth, either with or without urea. This broth contains phenol red, a colorimetric indicator that changes color based on the pH of the medium (UB+). Strains were then transferred to a U-bottom 96-well plate (Greiner, Cat #650185), in triplicate, with a minimum of two biological replicates. The OD was measured at 415 and 560 nm to measure pH-based color change, and at 600 nm to measure growth. Readings were taken every hour for 12 h while shaking at 37°C in the BioTek Synergy H1 microtiter plate reader. The ratio of ODs at 560 and 415 nm was used to calculate the color change in the media, which corresponds to the change in pH. Color change detected in the urease broth with urea (UB+) was then normalized to any color change detected in the urea-negative controls (UB−) to account for any pH changes that were not due to urease activity. This normalized ratio was visualized over the 12 h time course, and area under the curve values were calculated and graphed, as previously described (87).

### Human blood collection and plasma extraction

For human blood collection, donors were consented and enrolled prior to donation. Blood was drawn by a trained medical professional using a 22-gauge blood collection needle (BD, REF 368652) holder, and a tube (Henry Schein, ref #9870790) containing heparin. Human plasma was extracted from blood samples as previously described (88). Briefly, the tube containing heparin and blood was mixed with equal volumes of 3% dextran in saline and allowed to incubate statically at room temperature for 20 min to allow red blood cells to settle. The top plasma layer was removed, spun down for 10 min at 2,000 rpm at 4°C, and the supernatant was aliquoted in 1.7 mL tubes for storage at −20°C until use.

### Biofilm assays

Biofilm biomass was measured as previously described (89). Briefly, flat-bottom 96-well plates (Greiner, Cat #655185) were coated with 50 mM sodium bicarbonate (*in vitro* biofilm assay) or 20% HP diluted in 50 mM sodium bicarbonate overnight at 4°C (host protein-supplemented biofilm assay). The next day, excess HP (or sodium bicarbonate alone) was removed, and overnight cultures were diluted to an OD_600_ of 0.2 using a Synergy H1 Biotek microtiter plate reader (Biotek, Vermont) in various media, placed in the pre-coated 96-well plate, and incubated at 37°C overnight with gentle shaking. The next day, non-adherent bacteria were removed, wells were air-dried for 30 min, washed with water, and stained with 0.1% crystal violet for 10 min. Wells were washed again to remove excess crystal violet, and remaining crystal violet was solubilized in 33% acetic acid overnight at 4°C. The next day, 50 uL of each well was mixed with 50 uL of 33% acetic acid, and the OD_600_ was measured. A dilution factor of 1:2 was accounted for in the OD_600_ to determine the biomass of each biofilm.

### CAUTI mouse model

The mouse CAUTI model was performed as previously described (26, 61). Briefly, C57BL/6 female mice (Charles River Labs) were transurethrally implanted with a 3 mm piece of polyethylene tubing (Braintree Scientific; Cat #PE-10) encased in a 4 mm piece of silicone tubing (Braintree Scientific; Cat #SIL-205) and inoculated with approximately 2 × 10^7^ CFUs of bacteria. Bacterial strains were prepared for inoculation following growth in BHI for 18 h at 37°C, centrifugation at 10,000 rpm for 10 min, washing with 1× PBS, and finally resuspending in 1× PBS. After 1, 4, or 7 dpi, bladders, kidneys, spleens, and catheters were harvested, organs were weighed, and CFUs were enumerated to determine bacterial load.

### Cytokine profiling

Inflammatory cytokine profiling was performed as previously described (26). Briefly, bladder homogenates from UP 129 or UP 129∆*ureC* infected mice for 1, 4, and 7 dpi were frozen at −80°C until the assay was performed. Homogenates were thawed on ice and centrifuged for 10 min at 5,000 rpm to isolate the supernatant. Two to three homogenates for each strain at each time point were pooled, and protein concentrations were quantified and normalized to the lowest concentration. BSA was used to calculate standard curves. At least two pools per strain and per time point were assessed in duplicate. The BioPlex 23-Plex Assay Kit (BioRad, REF #M60009RDPD) was then used to assess cytokine levels within each pool according to the manufacturer’s instructions.

### Statistical analysis

Figures were graphed and statistics were performed using GraphPad Prism 10.4.2. All experiments included at least two biological replicates each, with three technical replicates in each biological replicate. For mouse experiments, each mouse was individually graphed in addition to the mean of each group. To reach a power of 0.8 using a significance cutoff of 0.05 for the mouse model, the Mann-Whitney *U* test power calculations determined that a minimum of eight mice per experimental group were required. Statistical analysis was performed with the Mann-Whitney *U* test with GraphPad Prism (version 10.5.0) software to determine significant differences between the wild-type and urease mutant strains. Transcriptional analysis graphs include the mean of each group and the standard deviation from the mean. The Mann-Whitney *U* test was used to compare transcription between various environmental conditions. Urease activity was graphed over time for 12 h. The area under each curve was then determined and compared between strains using the Mann-Whitney *U* test.

## SUPPLEMENTAL MATERIAL

File S1 mbio.01624-26-s0001.pdf Supplemental figures and legends for Files S2 and S3.

File S2 mbio.01624-26-s0002.pdf Sequenced pUre plasmids.

File S3 mbio.01624-26-s0003.xlsx Genomes used in this study.

ASM does not own the copyrights to Supplemental Material that may be linked to, or accessed through, an article. The authors have granted ASM a non-exclusive, world-wide license to publish the Supplemental Material files. Please contact the corresponding author directly for reuse.

## References

[1] Ronald A. 2003. The etiology of urinary tract infection: traditional and emerging pathogens. Dis Mon 49:71–82. doi:10.1067/mda.2003.8 12601338

[2] Trautner BW, Darouiche RO. 2004. Catheter-associated infections: pathogenesis affects prevention. Arch Intern Med 164:842–850. doi:10.1001/archinte.164.8.842 15111369 PMC2963580

[3] Cope M, Cevallos ME, Cadle RM, Darouiche RO, Musher DM, Trautner BW. 2009. Inappropriate treatment of catheter-associated asymptomatic bacteriuria in a tertiary care hospital. Clin Infect Dis 48:1182–1188. doi:10.1086/597403 19292664

[4] Trautner BW. 2010. Management of catheter-associated urinary tract infection. Curr Opin Infect Dis 23:76–82. doi:10.1097/QCO.0b013e328334dda8 19926986 PMC2865895

[5] Nicolle LE. 2012. Urinary catheter-associated infections. Infect Dis Clin North Am 26:13–27. doi:10.1016/j.idc.2011.09.009 22284373

[6] Chenoweth CE, Saint S. 2016. Urinary tract infections. Infect Dis Clin North Am 30:869–885. doi:10.1016/j.idc.2016.07.007 27816141

[7] Flores-Mireles AL, Walker JN, Caparon M, Hultgren SJ. 2015. Urinary tract infections: epidemiology, mechanisms of infection and treatment options. Nat Rev Microbiol 13:269–284. doi:10.1038/nrmicro3432 25853778 PMC4457377

[8] Gaston JR, Johnson AO, Bair KL, White AN, Armbruster CE. 2021. Polymicrobial interactions in the urinary tract: is the enemy of my enemy my friend? Infect Immun 89:e00652-20. doi:10.1128/IAI.00652-20 33431702

[9] Armbruster CE, Brauer AL, Humby MS, Shao J, Chakraborty S. 2021. Prospective assessment of catheter-associated bacteriuria clinical presentation, epidemiology, and colonization dynamics in nursing home residents. JCI Insight 6. doi:10.1172/jci.insight.144775 PMC8525589 34473649

[10] Walker JN, Flores-Mireles AL, Lynch AJL, Pinkner C, Caparon MG, Hultgren SJ, Desai A. 2020. High-resolution imaging reveals microbial biofilms on patient urinary catheters despite antibiotic administration. World J Urol 38:2237–2245. doi:10.1007/s00345-019-03027-8 31792577 PMC7778452

[11] Nye TM, Zou Z, Obernuefemann CLP, Pinkner JS, Lowry E, Kleinschmidt K, Bergeron K, Klim A, Dodson KW, Flores-Mireles AL, Walker JN, Wong DG, Desai A, Caparon MG, Hultgren SJ. 2024. Microbial co-occurrences on catheters from long-term catheterized patients. Nat Commun 15:61. doi:10.1038/s41467-023-44095-0 38168042 PMC10762172

[12] Walker J, Ramirez JD, Armbruster C, Hanson B. 2025. Genomics reveal *Staphylococcus aureus* persists during long-term urinary catheterization despite antimicrobial therapy and catheter exchanges. Research Square. doi:10.21203/rs.3.rs-6702271/v1 PMC12873216 41530149

[13] Broomfield RJ, Morgan SD, Khan A, Stickler DJ. 2009. Crystalline bacterial biofilm formation on urinary catheters by urease-producing urinary tract pathogens: a simple method of control. J Med Microbiol 58:1367–1375. doi:10.1099/jmm.0.012419-0 19556373

[14] Stickler DJ, Feneley RCL. 2010. The encrustation and blockage of long-term indwelling bladder catheters: a way forward in prevention and control. Spinal Cord 48:784–790. doi:10.1038/sc.2010.32 20368711

[15] Morris NS, Stickler DJ, McLean RJC. 1999. The development of bacterial biofilms on indwelling urethral catheters. World J Urol 17:345–350. doi:10.1007/s003450050159 10654364

[16] Marien T, Miller NL. 2015. Treatment of the infected stone. Urol Clin North Am 42:459–472. doi:10.1016/j.ucl.2015.05.009 26475943

[17] Karplus PA, Pearson MA, Hausinger RP. 1997. 70 years of crystalline urease: what have we learned? Acc Chem Res 30:330–337. doi:10.1021/ar960022j

[18] Stickler DJ. 2014. Clinical complications of urinary catheters caused by crystalline biofilms: something needs to be done. J Intern Med 276:120–129. doi:10.1111/joim.12220 24635559

[19] Stickler DJ, Zimakoff J. 1994. Complications of urinary tract infections associated with devices used for long-term bladder management. J Hosp Infect 28:177–194. doi:10.1016/0195-6701(94)90101-5 7852732

[20] Hedelin H. 2002. Uropathogens and urinary tract concretion formation and catheter encrustations. Int J Antimicrob Agents 19:484–487. doi:10.1016/s0924-8579(02)00095-x 12135838

[21] Al Mohajer M, Musher DM, Minard CG, Darouiche RO. 2013. Clinical significance of *Staphylococcus aureus* bacteriuria at a tertiary care hospital. Scand J Infect Dis 45:688–695. doi:10.3109/00365548.2013.803291 23808717

[22] Muder RR, Brennen C, Rihs JD. 2015. Isolation of *Staphylococcus aureus* from the urinary tract: association of isolation with symptomatic urinary tract infection and subsequent staphylococcal bacteremia. Clin Infect Dis 42:46–50. doi:10.1086/498518 16323090

[23] Nicolle LE. 2015. Asymptomatic bacteriuria and bacterial interference. Microbiol Spectr 3. doi:10.1128/microbiolspec.UTI-0001-2012 26542046

[24] Xu K, Wang Y, Jian Y, Chen T, Liu Q, Wang H, Li M, He L. 2023. *Staphylococcus aureus* ST1 promotes persistent urinary tract infection by highly expressing the urease. Front Microbiol 14:1101754. doi:10.3389/fmicb.2023.1101754 36910215 PMC9992547

[25] Zhou C, Bhinderwala F, Lehman MK, Thomas VC, Chaudhari SS, Yamada KJ, Foster KW, Powers R, Kielian T, Fey PD. 2019. Urease is an essential component of the acid response network of *Staphylococcus aureus* and is required for a persistent murine kidney infection. PLoS Pathog 15:e1007538. doi:10.1371/journal.ppat.1007538 30608981 PMC6343930

[26] Walker JN, Flores-Mireles AL, Pinkner CL, Schreiber HL 4th, Joens MS, Park AM, Potretzke AM, Bauman TM, Pinkner JS, Fitzpatrick JAJ, Desai A, Caparon MG, Hultgren SJ. 2017. Catheterization alters bladder ecology to potentiate *Staphylococcus aureus* infection of the urinary tract. Proc Natl Acad Sci USA 114:E8721–E8730. doi:10.1073/pnas.1707572114 28973850 PMC5642702

[27] Flores-Mireles AL, Walker JN, Bauman TM, Potretzke AM, Schreiber HL 4th, Park AM, Pinkner JS, Caparon MG, Hultgren SJ, Desai A. 2016. Fibrinogen release and deposition on urinary catheters placed during urological procedures. J Urol 196:416–421. doi:10.1016/j.juro.2016.01.100 26827873 PMC4965327

[28] Seidl K, Müller S, François P, Kriebitzsch C, Schrenzel J, Engelmann S, Bischoff M, Berger-Bächi B. 2009. Effect of a glucose impulse on the CcpA regulon in *Staphylococcus aureus*. BMC Microbiol 9:95. doi:10.1186/1471-2180-9-95 19450265 PMC2697999

[29] Crosby HA, Tiwari N, Kwiecinski JM, Xu Z, Dykstra A, Jenul C, Fuentes EJ, Horswill AR. 2020. The *Staphylococcus aureus* ArlRS two-component system regulates virulence factor expression through MgrA. Mol Microbiol 113:103–122. doi:10.1111/mmi.14404 31618469 PMC7175635

[30] Subramaniyan Y, Mujeeburahiman M, Khan A, Rekha PD. 2025. Overexpression of Agr quorum sensing system in uropathogenic *Staphylococcus aureus* in response to simulated urinary metabolic conditions enhancing growth and virulence. Med Microbiol Immunol 214:35. doi:10.1007/s00430-025-00830-6 40762857

[31] Jenul C, Horswill AR. 2019. Regulation of *Staphylococcus aureus* virulence. Microbiol Spectr 7. doi:10.1128/microbiolspec.gpp3-0031-2018 PMC6452892 30953424

[32] Novick RP. 2003. Autoinduction and signal transduction in the regulation of staphylococcal virulence. Mol Microbiol 48:1429–1449. doi:10.1046/j.1365-2958.2003.03526.x 12791129

[33] Brinsmade SR. 2017. CodY, a master integrator of metabolism and virulence in gram-positive bacteria. Curr Genet 63:417–425. doi:10.1007/s00294-016-0656-5 27744611

[34] Seidl K, Stucki M, Ruegg M, Goerke C, Wolz C, Harris L, Berger-Bächi B, Bischoff M. 2006. *Staphylococcus aureus* CcpA affects virulence determinant production and antibiotic resistance. Antimicrob Agents Chemother 50:1183–1194. doi:10.1128/AAC.50.4.1183-1194.2006 16569828 PMC1426959

[35] Duran Ramirez JM, Gomez J, Obernuefemann CLP, Gualberto NC, Walker JN. 2022. Semi-quantitative assay to measure urease activity by urinary catheter-associated uropathogens. Front Cell Infect Microbiol 12:859093. doi:10.3389/fcimb.2022.859093 35392611 PMC8980526

[36] Konieczna I, Arnowiec P, Kwinkowski M. 2012. Bacterial urease and its role in long-lasting human diseases. Available from: http://mgv2.cmbi.ru.nl 10.2174/138920312804871094 PMC3816311 23305365

[37] Flannigan R, Choy WH, Chew B, Lange D. 2014. Renal struvite stones—pathogenesis, microbiology, and management strategies. Nat Rev Urol 11:333–341. doi:10.1038/nrurol.2014.99 24818849

[38] Bichler KH, Eipper E, Naber K, Braun V, Zimmermann R, Lahme S. 2002. Urinary infection stones. Int J Antimicrob Agents 19:488–498. doi:10.1016/S0924-8579(02)00088-2 12135839

[39] Miwa Y, Nakata A, Ogiwara A, Yamamoto M, Fujita Y. 2000. Evaluation and characterization of catabolite-responsive elements (cre) of *Bacillus subtilis*. Nucleic Acids Res 28:1206–1210. doi:10.1093/nar/28.5.1206 10666464 PMC102602

[40] Petersohn A, Jo¨ J, Bernhardt J. 1999. Identification of B-dependent genes in Bacillus subtilis using a promoter consensus-directed search and oligonucleotide hybridization. Vol. 181. https://journals.asm.org/journal/jb. 10.1128/jb.181.18.5718-5724.1999 PMC94092 10482513

[41] Mashruwala AA, Boyd JM. 2017. The *Staphylococcus aureus* SrrAB regulatory system modulates hydrogen peroxide resistance factors, which imparts protection to aconitase during aerobic growth. PLoS One 12:e0170283. doi:10.1371/journal.pone.0170283 28099473 PMC5242492

[42] Gao Y, Poudel S, Seif Y, Shen Z, Palsson BO. 2023. Elucidating the CodY regulon in *Staphylococcus aureus* USA300 substrains TCH1516 and LAC. mSystems 8:e0027923. doi:10.1128/msystems.00279-23 37310465 PMC10470025

[43] Sun F, Li C, Jeong D, Sohn C, He C, Bae T. 2010. In the *Staphylococcus aureus* two-component system sae, the response regulator SaeR binds to a direct repeat sequence and DNA binding requires phosphorylation by the sensor kinase SaeS. J Bacteriol 192:2111–2127. doi:10.1128/JB.01524-09 20172998 PMC2849438

[44] Rajasree K, Fasim A, Gopal B. 2016. Conformational features of the *Staphylococcus aureus* AgrA-promoter interactions rationalize quorum-sensing triggered gene expression. Biochem Biophys Rep 6:124–134. doi:10.1016/j.bbrep.2016.03.012 28955870 PMC5600425

[45] Prados J, Linder P, Redder P. 2016. TSS-EMOTE, a refined protocol for a more complete and less biased global mapping of transcription start sites in bacterial pathogens. BMC Genomics 17:849. doi:10.1186/s12864-016-3211-3 27806702 PMC5094136

[46] McNamara PJ, Milligan-Monroe KC, Khalili S, Proctor RA. 2000. Identification, cloning, and initial characterization of rot, a locus encoding a regulator of virulence factor expression in *Staphylococcus aureus*. J Bacteriol 182:3197–3203. doi:10.1128/JB.182.11.3197-3203.2000 10809700 PMC94507

[47] Chan PF, Foster SJ, Ingham E, Clements MO. 1998. The *Staphylococcus aureus* alternative sigma factor sigmaB controls the environmental stress response but not starvation survival or pathogenicity in a mouse abscess model. J Bacteriol 180:6082–6089. doi:10.1128/JB.180.23.6082-6089.1998 9829915 PMC107691

[48] Kaiser JC, King AN, Grigg JC, Sheldon JR, Edgell DR, Murphy MEP, Brinsmade SR, Heinrichs DE. 2018. Repression of branched-chain amino acid synthesis in *Staphylococcus aureus* is mediated by isoleucine via CodY, and by a leucine-rich attenuator peptide. PLoS Genet 14:e1007159. doi:10.1371/journal.pgen.1007159 29357354 PMC5794164

[49] Karathanasi G, Bojer MS, Baldry M, Johannessen BA, Wolff S, Greco I, Kilstrup M, Hansen PR, Ingmer H. 2018. Linear peptidomimetics as potent antagonists of *Staphylococcus aureus* agr quorum sensing. Sci Rep 8:3562. doi:10.1038/s41598-018-21951-4 29476092 PMC5824847

[50] Kinkel TL, Roux CM, Dunman PM, Fang FC. 2013. The *Staphylococcus aureus* SrrAB two-component system promotes resistance to nitrosative stress and hypoxia. mBio 4:e00696-13. doi:10.1128/mBio.00696-13 24222487 PMC3892780

[51] Geiger T, Goerke C, Mainiero M, Kraus D, Wolz C. 2008. The virulence regulator Sae of *Staphylococcus aureus*: promoter activities and response to phagocytosis-related signals. J Bacteriol 190:3419–3428. doi:10.1128/JB.01927-07 18344360 PMC2395011

[52] Bischoff M, Entenza JM, Giachino P. 2001. Influence of a functional *sigB* operon on the global regulators *sar* and *agr* in *Staphylococcus aureus*. J Bacteriol 183:5171–5179. doi:10.1128/JB.183.17.5171-5179.2001 11489871 PMC95394

[53] Novick RP, Jiang D. 2003. The staphylococcal *saeRS* system coordinates environmental signals with *agr* quorum sensing. Microbiology (Reading) 149:2709–2717. doi:10.1099/mic.0.26575-0 14523104

[54] Recsei P, Kreiswirth B, O’Reilly M, Schlievert P, Gruss A, Novick RP. 1986. Regulation of exoprotein gene expression in *Staphylococcus aureus* by agar. Mol Gen Genet 202:58–61. doi:10.1007/BF00330517 3007938

[55] Roux A, Todd DA, Velázquez JV, Cech NB, Sonenshein AL. 2014. CodY-mediated regulation of the *Staphylococcus aureus* Agr system integrates nutritional and population density signals. J Bacteriol 196:1184–1196. doi:10.1128/JB.00128-13 24391052 PMC3957711

[56] Roberts GA, Houston PJ, White JH, Chen K, Stephanou AS, Cooper LP, Dryden DTF, Lindsay JA. 2013. Impact of target site distribution for Type I restriction enzymes on the evolution of methicillin-resistant *Staphylococcus aureus* (MRSA) populations. Nucleic Acids Res 41:7472–7484. doi:10.1093/nar/gkt535 23771140 PMC3753647

[57] Wright ES. 2020. RNAconTest: comparing tools for noncoding RNA multiple sequence alignment based on structural consistency. RNA 26:531–540. doi:10.1261/rna.073015.119 32005745 PMC7161358

[58] Resch A, Rosenstein R, Nerz C, Götz F. 2005. Differential gene expression profiling of *Staphylococcus aureus* cultivated under biofilm and planktonic conditions. Appl Environ Microbiol 71:2663–2676. doi:10.1128/AEM.71.5.2663-2676.2005 15870358 PMC1087559

[59] Xu W, Chen J, McLellan LK, Flores-Mireles AL, Hunstad DA, Caparon MG. 2025. Host cysteine proteases promote the severity of catheter-associated urinary tract infection and kidney fibrosis. mBio 16:e02161-25. doi:10.1128/mbio.02161-25 40980878 PMC12607876

[60] Bouatra S, Aziat F, Mandal R, Guo AC, Wilson MR, Knox C, Bjorndahl TC, Krishnamurthy R, Saleem F, Liu P, Dame ZT, Poelzer J, Huynh J, Yallou FS, Psychogios N, Dong E, Bogumil R, Roehring C, Wishart DS. 2013. The human urine metabolome. PLoS One 8:e73076. doi:10.1371/journal.pone.0073076 24023812 PMC3762851

[61] Conover MS, Flores-Mireles AL, Hibbing ME, Dodson K, Hultgren SJ. 2015. Establishment and characterization of UTI and CAUTI in a mouse model. J Vis Exp:e52892. doi:10.3791/52892 26132341 PMC4544389

[62] Guiton PS, Hannan TJ, Ford B, Caparon MG, Hultgren SJ. 2013. *Enterococcus faecalis* overcomes foreign body-mediated inflammation to establish urinary tract infections. Infect Immun 81:329–339. doi:10.1128/IAI.00856-12 23132492 PMC3536162

[63] Bhattacharya P, Budnick I, Singh M, Thiruppathi M, Alharshawi K, Elshabrawy H, Holterman MJ, Prabhakar BS. 2015. Dual Role of GM-CSF as a pro-inflammatory and a regulatory cytokine: implications for immune therapy. J Inter Cytokine Res 35:585–599. doi:10.1089/jir.2014.0149 PMC4529096 25803788

[64] Conti P, Pang X, Boucher W, Letourneau R, Reale M, Barbacane RC, Thibault J, Theoharides TC. 1997. RANTES is a pro-inflammatory chemokine and chemoattracts basophil cells to extravascular sites. J Pathol 183:352–358. doi:10.1002/(SICI)1096-9896(199711)183:33.0.CO;2-L 9422993

[65] Lodeta B, Lovrinic D, Lodeta M, Zavidic T, Baric H. 2018. Use of urinary collection devices in community and nursing homes in Istria County. Urol Int 100:333–338. doi:10.1159/000486900 29502119

[66] Czwikla J, Schmiemann G, Hoffmann F. 2024. Use of indwelling urinary catheters in nursing home residents: results from a cross-sectional study in 21 German nursing homes. BMC Urol 24:125. doi:10.1186/s12894-024-01512-w 38877475 PMC11177429

[67] Remy L, Carrière M, Derré-Bobillot A, Martini C, Sanguinetti M, Borezée-Durant E. 2013. The *Staphylococcus aureus* Opp1 ABC transporter imports nickel and cobalt in zinc-depleted conditions and contributes to virulence. Mol Microbiol 87:730–743. doi:10.1111/mmi.12126 23279021

[68] Hiron A, Posteraro B, Carrière M, Remy L, Delporte C, La Sorda M, Sanguinetti M, Juillard V, Borezée-Durant E. 2010. A nickel ABC-transporter of *Staphylococcus aureus* is involved in urinary tract infection. Mol Microbiol 77:1246–1260. doi:10.1111/j.1365-2958.2010.07287.x 20662775

[69] Sarigul N, Korkmaz F, Kurultak İ. 2019. A new artificial urine protocol to better imitate human urine. Sci Rep 9. doi:10.1038/s41598-019-56693-4 PMC6934465 31882896

[70] Waters NR, Samuels DJ, Behera RK, Livny J, Rhee KY, Sadykov MR, Brinsmade SR. 2016. A spectrum of CodY activities drives metabolic reorganization and virulence gene expression in *Staphylococcus aureus*. Mol Microbiol 101:495–514. doi:10.1111/mmi.13404 27116338 PMC5007081

[71] Poudel S, Hefner Y, Szubin R, Sastry A, Gao Y, Nizet V, Palsson BO. 2022. Coordination of CcpA and CodY regulators in *Staphylococcus aureus* USA300 strains. mSystems 7:e00480-22. doi:10.1128/msystems.00480-22 36321827 PMC9765215

[72] Bulock LL, Ahn J, Shinde D, Pandey S, Sarmiento C, Thomas VC, Guda C, Bayles KW, Sadykov MR. 2022. Interplay of CodY and CcpA in regulating central metabolism and biofilm formation in *Staphylococcus aureus*. J Bacteriol 204:e00617-21. doi:10.1128/jb.00617-21 35735992 PMC9295537

[73] Grosser MR, Weiss A, Shaw LN, Richardson AR. 2016. Regulatory requirements for *Staphylococcus aureus* nitric oxide resistance. J Bacteriol 198:2043–2055. doi:10.1128/JB.00229-16 27185828 PMC4944221

[74] Somerville GA, Proctor RA. 2009. At the crossroads of bacterial metabolism and virulence factor synthesis in *Staphylococci*. Microbiol Mol Biol Rev 73:233–248. doi:10.1128/MMBR.00005-09 19487727 PMC2698418

[75] Jacobsen SM, Shirtliff ME. 2011. Proteus mirabilis biofilms and catheter-associated urinary tract infections. Virulence 2:460–465. doi:10.4161/viru.2.5.17783 21921687

[76] Andersen MJ, Fong C, La Bella AA, Molina JJ, Molesan A, Champion MM, Howell C, Flores-Mireles AL. 2022. Inhibiting host-protein deposition on urinary catheters reduces associated urinary tract infections. eLife 11:e75798. doi:10.7554/eLife.75798 35348114 PMC8986317

[77] Flores-Mireles AL, Walker JN, Potretzke A, Schreiber HL 4th, Pinkner JS, Bauman TM, Park AM, Desai A, Hultgren SJ, Caparon MG. 2016. Antibody-based therapy for enterococcal catheter-associated urinary tract infections. mBio 7:e01653-16. doi:10.1128/mBio.01653-16 27795399 PMC5080383

[78] Raffi HS, Bates JM, Flournoy DJ, Kumar S. 2012. Tamm-Horsfall protein facilitates catheter associated urinary tract infection. BMC Res Notes 5:532. doi:10.1186/1756-0500-5-532 23009031 PMC3517338

[79] Yıldırım İ, Koçan H. 2023. The pH of drinking water and its effect on the pH of urine. Cureus 15:e47437. doi:10.7759/cureus.47437 38022142 PMC10659234

[80] Bae T, Glass EM, Schneewind O, Missiakas D. 2008. Generating a collection of insertion mutations in the *Staphylococcus aureus* genome using *bursa aurealis*. Methods Mol Microbiol 416:103–116. doi:10.1007/978-1-59745-321-9_7 18392963

[81] Fey PD, Endres JL, Yajjala VK, Widhelm TJ, Boissy RJ, Bose JL, Bayles KW. 2013. A genetic resource for rapid and comprehensive phenotype screening of nonessential *Staphylococcus aureus* genes. mBio 4:e00537-12. doi:10.1128/mBio.00537-12 23404398 PMC3573662

[82] Nair D, Memmi G, Hernandez D, Bard J, Beaume M, Gill S, Francois P, Cheung AL. 2011. Whole-genome sequencing of *Staphylococcus aureus* strain RN4220, a key laboratory strain used in virulence research, identifies mutations that affect not only virulence factors but also the fitness of the strain. J Bacteriol 193:2332–2335. doi:10.1128/JB.00027-11 21378186 PMC3133102

[83] Lauderdale KJ, Malone CL, Boles BR, Morcuende J, Horswill AR. 2010. Biofilm dispersal of community-associated methicillin-resistant *Staphylococcus aureus* on orthopedic implant material. J Orthop Res 28:55–61. doi:10.1002/jor.20943 19610092

[84] Wörmann ME, Reichmann NT, Malone CL, Horswill AR, Gründling A. 2011. Proteolytic cleavage inactivates the *Staphylococcus aureus* lipoteichoic acid synthase. J Bacteriol 193:5279–5291. doi:10.1128/JB.00369-11 21784926 PMC3187375

[85] Walker JN, Crosby HA, Spaulding AR, Salgado-Pabón W, Malone CL, Rosenthal CB, Schlievert PM, Boyd JM, Horswill AR. 2013. The *Staphylococcus aureus* ArlRS two-component system is a novel regulator of agglutination and pathogenesis. PLoS Pathog 9:e1003819. doi:10.1371/journal.ppat.1003819 24367264 PMC3868527

[86] Malone CL, Boles BR, Lauderdale KJ, Thoendel M, Kavanaugh JS, Horswill AR. 2009. Fluorescent reporters for *Staphylococcus aureus*. J Microbiol Methods 77:251–260. doi:10.1016/j.mimet.2009.02.011 19264102 PMC2693297

[87] Guterman LB, Motsay M, Hunt BC, Brauer AL, Learman BS, Wijayahena MK, Hoepker AC, Aga DS, Francis B, Fontoura BM, Donati GL, Bush PJ, Deka N, Armbruster CE. 2025. Harnessing microbial-derived metabolites in the urinary tract to prevent infection induced catheter encrustation. Nat Commun 16:9678. doi:10.1038/s41467-025-64661-y 41184242 PMC12583827

[88] Quinn MT, Deleo FR. 2014. Neutrophil methods and protocols second edition methods in molecular biology 1124. Available from: http://www.springer.com/series/7651

[89] Duran Ramirez JM, Gomez J, Hanson BM, Isa T, Myckatyn TM, Walker JN. 2023. *Staphylococcus aureus* breast implant infection isolates display recalcitrance to antibiotic pocket irrigants. Microbiol Spectr 11:e02884-22. doi:10.1128/spectrum.02884-22 36507629 PMC9927092

