# Supplementary material for: Staphylococcus aureus urease is controlled by a complex regulatory network to promote dissemination during CAUTI
Source: mBio. 2026 Aug 5;17(9):e01624-26. doi: 10.1128/mbio.01624-26 (PMC13556217; doi:10.1128/mbio.01624-26)
Supplement: Supplementary file 1 [file mbio.01624-26-s0001.pdf]

SUPPLEMENTAL FIGURES

A.

B.

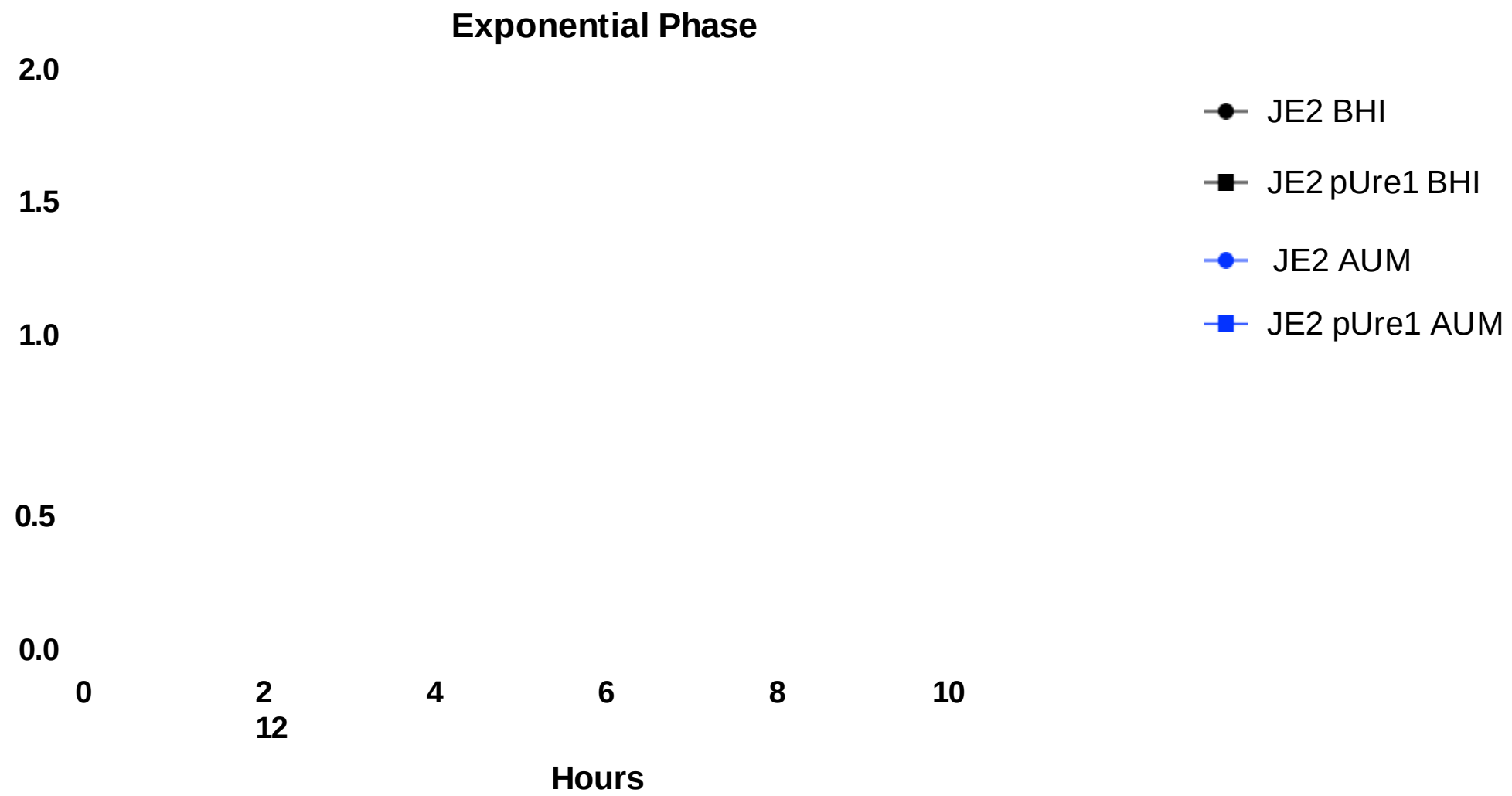

C.

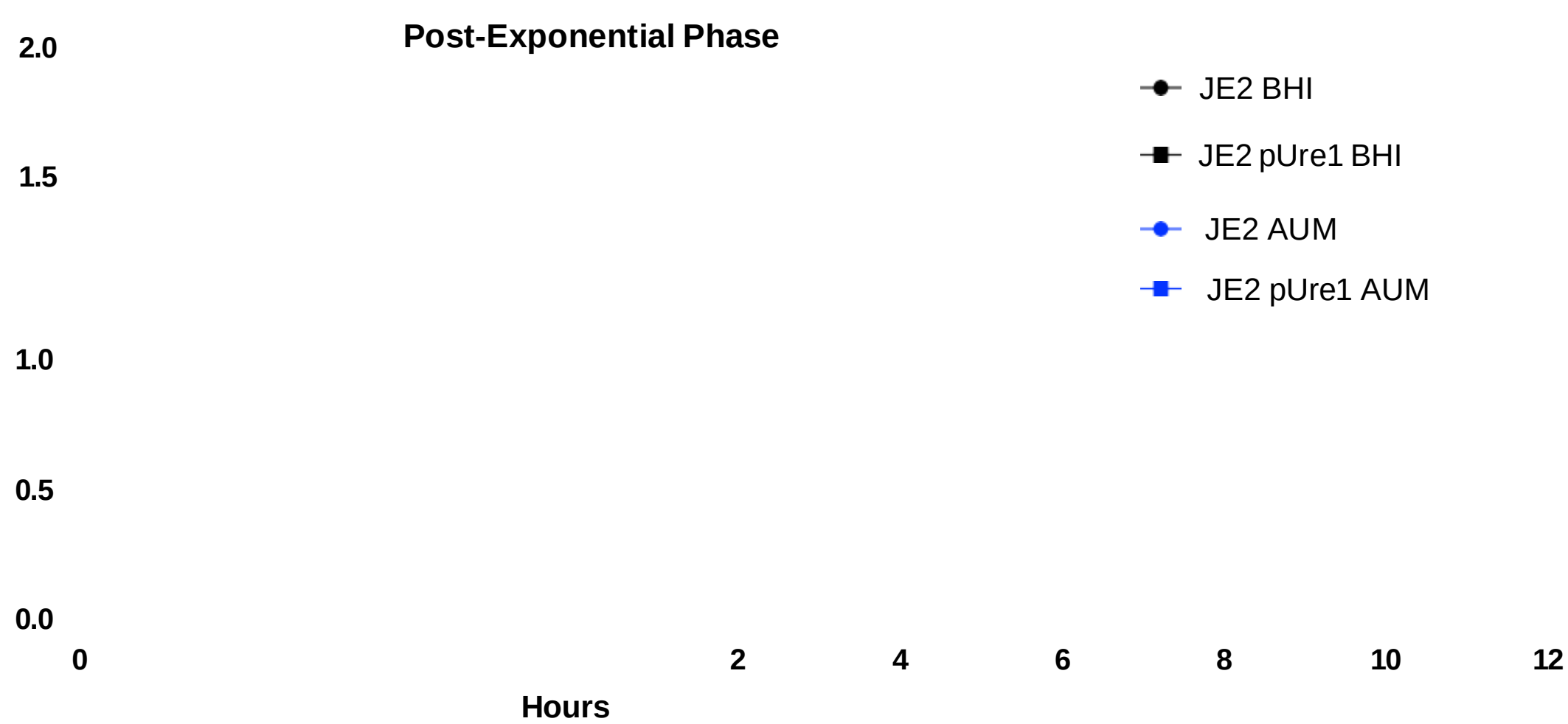

Figure S1: Creation and growth of JE2 pUre1 strain. **A)** Confirmation of pUre1 plasmid in the JE2 background. Presence of the pUre1 plasmid was confirmed with the pGFP check F and R primers

found in Table 2. (-) indicates DNA from wild type JE2 and (+) indicates DNA from JE2 pUre1. **B and C)** Growth of the JE2 and JE2 pUre1 strains in BHI and AUM. Cultures were started at either exponential phase (**B**) or post-exponential phase (**C**). Growth was measured via OD_{600} for a 12-hour period in either BHI (black lines) or AUM (blue lines). Dots indicate the mean of all replicates and lines indicate the standard deviation.

A.

Exponential Phase

5000
4000
3000
2000
1000
0
-1000
-2000

BHI
AUM
HU
HU:S
UB-
UB+

BHI
AUM
HU
HU:S
UB-
UB+

C.

Urease Expression

*

50000
40000
30000
20000
10000
0

BHI AUM HU BHI AUM
HU

EXP

POST

D.

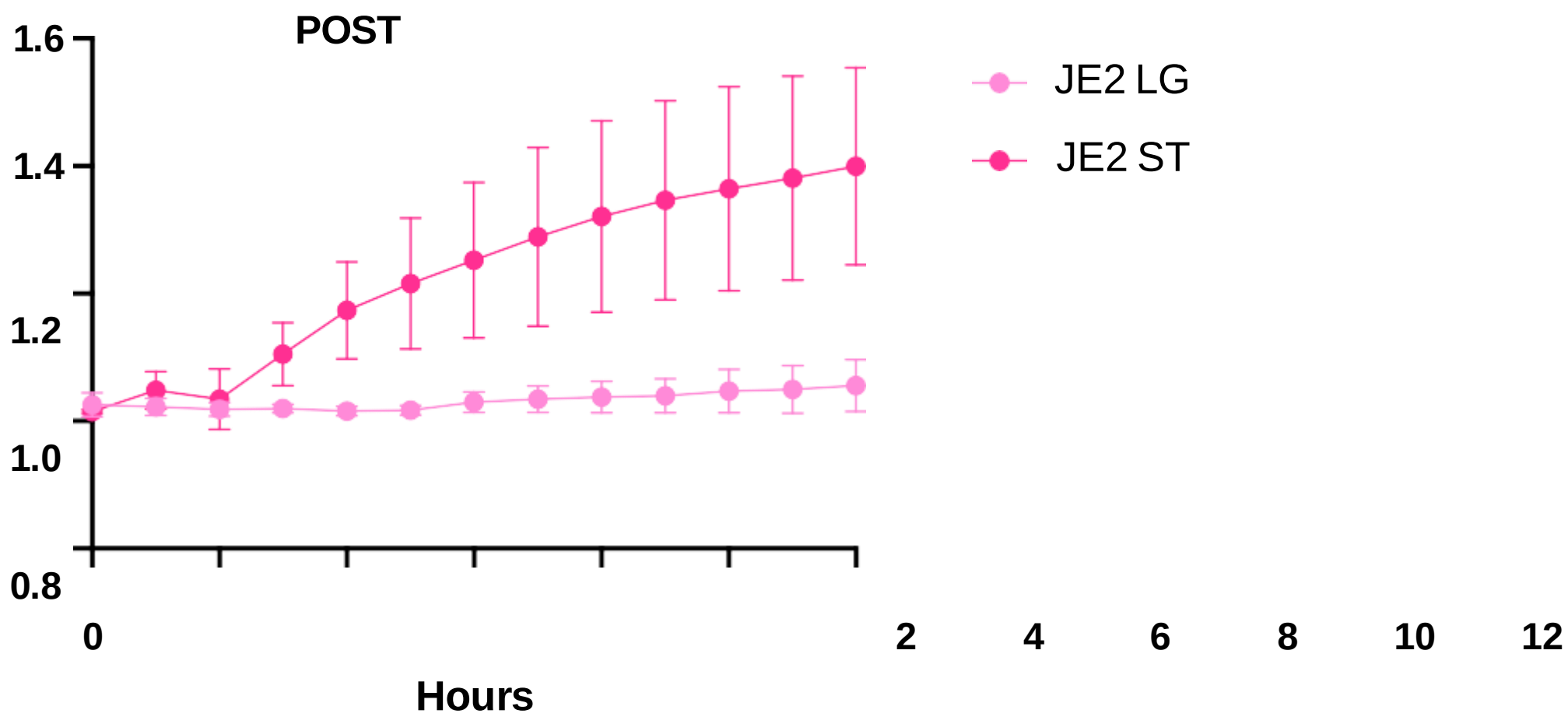

Figure S2: Urease expression and activity curves. Urease expression over a 12 hr time course with cells beginning in either exponential phase (EXP) (**A**) or post-exponential phase (POST) (**B**) in various media conditions. Fluorescence was normalized to the fluorescence reading of the background strain that did not contain the GFP reporter plasmid. Fluorescence was measured in BHI (black lines), AUM (blue lines), female HU (green lines), HU:S (yellow lines), UB with no urea (purple lines), or UB with urea (pink lines). **C**) Area under the curve analysis comparing BHI, AUM, and HU. Significance was determined using the Mann-Whitney U test. **D**) Relative urease activity over a 12 hr time course with cells beginning in either exponential phase (dark pink) or post-exponential phase (light pink). Dots indicate the mean of all replicates and lines indicate the standard deviation.

A.

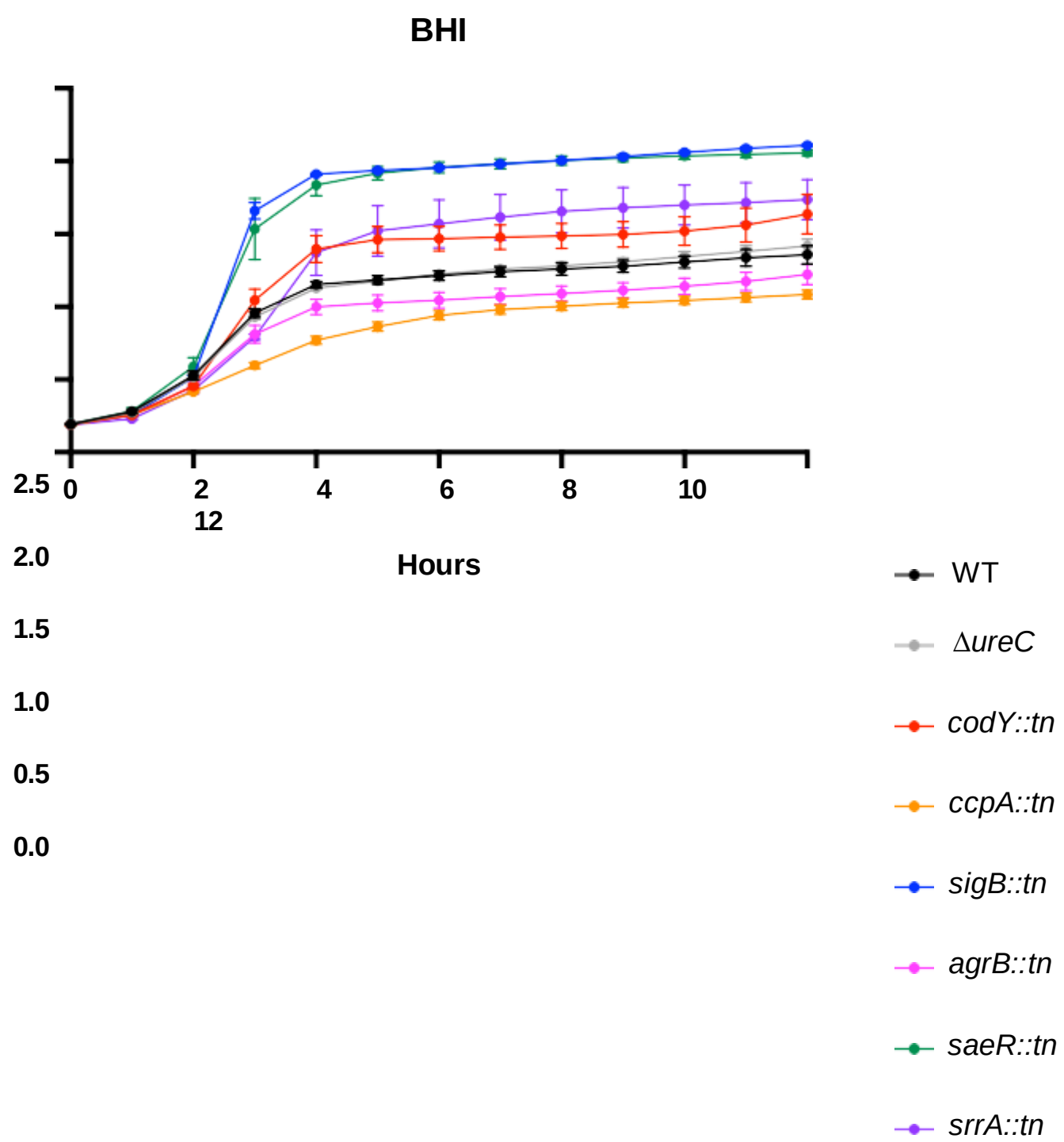

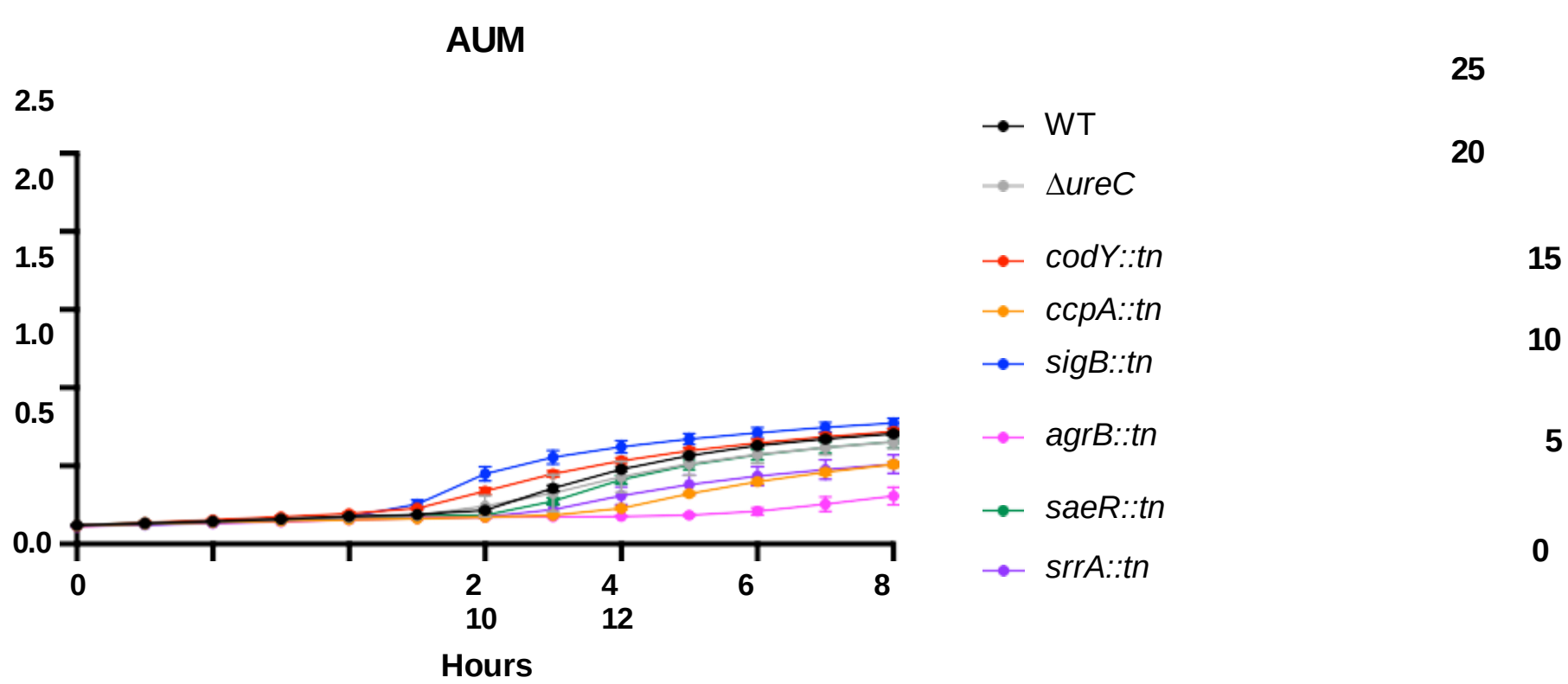

Figure S3: Generation and growth of transposon mutants of urease regulators in JE2. **A)** PCR confirmation of transposon mutants of *codY, ccpA, sigB, agrB, saeR,* and *srrA* in the JE2 background strain. The forward and reverse primers for each gene (**Table 2**) were used to amplify DNA from wild type JE2 (1) and the JE2 transposon mutant (2). Due to the presence of the transposon, the DNA from wild type (1) had a smaller band size compared to the corresponding transposon mutant DNA (2). The forward primer of each gene and the buster primer (**Table 2**) were used to amplify DNA from

wild type JE2 (3) and the JE2 transposon mutant (4). Due to the lack of a transposable element in the

wild type (3), no band was amplified. The presence of the transposable element in the mutant strains allowed for binding and amplification of the buster primer (4). Growth and area under the curve analyses for transposon mutant strains in BHI (**B and C**) and AUM (**D and E**). Growth was measured via OD_{600} over 12-hours. Each dot represents a technical replicate. At least two biological replicates were performed. Bars represent the mean of all replicates with the standard deviation. Area under the curve was calculated for each strain (**C and E**). Significance was determined using the Mann-Whitney U test.

Exponential Phase

Post-Exponential Phase

A.

BHI

1000

0

-1000

-2000

WT
codY
ccpA
sigB
agrB
saeR
srrA

BHI

WT
codY
ccpA
sigB
agrB
saeR
srrA

C.

AUM

WT
codY
ccpA
sigB
agrB
saeR
srrA

1000

0

-1000

-2000

D.

AUM

WT
codY
ccpA
sigB
agrB
saeR
srrA

E.

HU

6000

4000

2000

0

-2000

WT
codY
ccpA
sigB
agrB
saeR
srrA

F.

HU

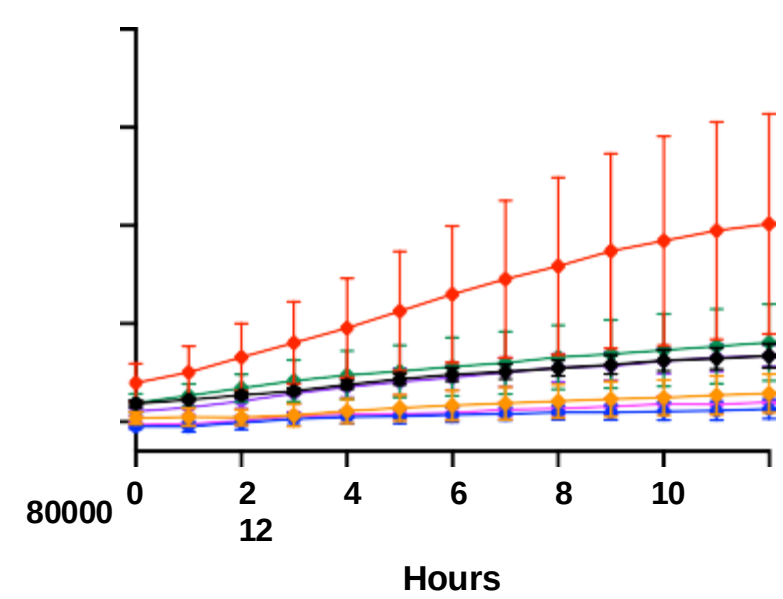

80000

60000

40000

20000

0

WT
codY
ccpA
sigB
agrB
saeR
srrA

Figure S4: Role of predicted regulators in urease expression. Urease expression over a 12 hr time course for the regulator mutant strains in either exponential (**A, C, and E**) or post-exponential (**B, D, and F**) phase growth in BHI (**A and B**), AUM (**C and D**), or HU (**E and F**). Dots indicate the mean of all replicates and lines indicate the standard deviation.

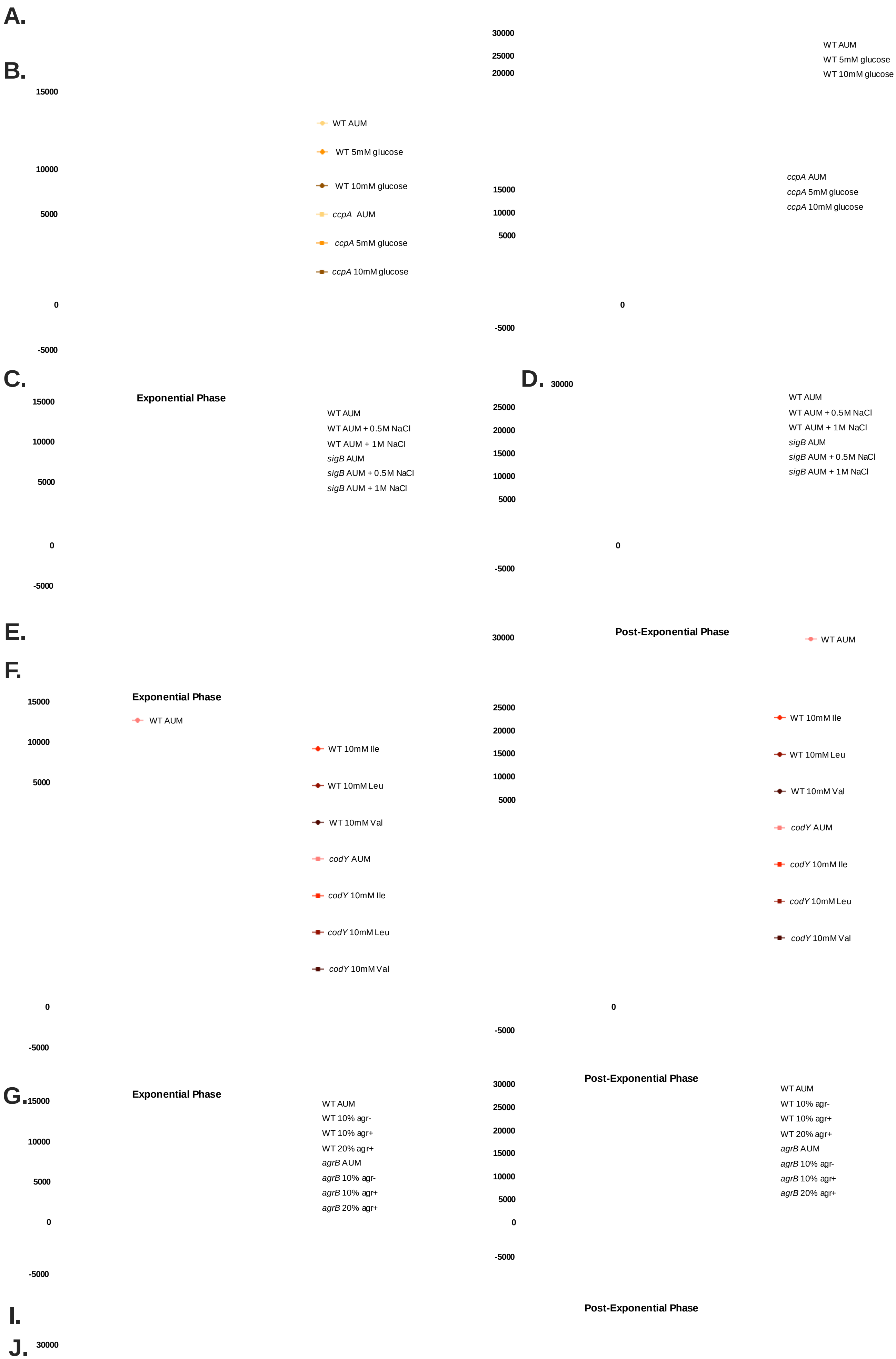
A.
30000
25000
20000
15000
10000
5000
0
-5000
WT AUM
WT 5mM glucose
WT 10mM glucose
ccpA AUM
ccpA 5mM glucose
ccpA 10mM glucose
B.
15000
10000
5000
0
-5000
WT AUM
WT 5mM glucose
WT 10mM glucose
ccpA AUM
ccpA 5mM glucose
ccpA 10mM glucose
C.
Exponential Phase
15000
10000
5000
0
-5000
WT AUM
WT AUM + 0.5M NaCl
WT AUM + 1M NaCl
sigB AUM
sigB AUM + 0.5M NaCl
sigB AUM + 1M NaCl
D.
30000
25000
20000
15000
10000
5000
0
-5000
WT AUM
WT AUM + 0.5M NaCl
WT AUM + 1M NaCl
sigB AUM
sigB AUM + 0.5M NaCl
sigB AUM + 1M NaCl
E.
Post-Exponential Phase
30000
25000
20000
15000
10000
5000
0
-5000
WT AUM
WT 10mM Ile
WT 10mM Leu
WT 10mM Val
codY AUM
codY 10mM Ile
codY 10mM Leu
codY 10mM Val
F.
Exponential Phase
15000
10000
5000
0
-5000
WT AUM
WT 10mM Ile
WT 10mM Leu
WT 10mM Val
codY AUM
codY 10mM Ile
codY 10mM Leu
codY 10mM Val
Post-Exponential Phase
30000
25000
20000
15000
10000
5000
0
-5000
WT AUM
WT 10% agr-
WT 10% agr+
WT 20% agr+
agrB AUM
agrB 10% agr-
agrB 10% agr+
agrB 20% agr+
G.
Exponential Phase
15000
10000
5000
0
-5000
WT AUM
WT 10% agr-
WT 10% agr+
WT 20% agr+
agrB AUM
agrB 10% agr-
agrB 10% agr+
agrB 20% agr+
I.
Post-Exponential Phase
J.
30000

Exponential Phase

15000
10000
5000
0
-5000

WT AUM
WT 0.5mM DEA/NO
WT 1mM DEA/NO
srrA AUM
srrA 0.5mM DEA/NO
srrA 1mM DEA/NO

25000
20000
15000
10000
5000
0
-5000

WT AUM
WT 0.5mM DEA/NO
WT 1mM DEA/NO
srrA AUM
srrA 0.5mM DEA/NO
srrA 1mM DEA/NO

K.

L.

15000
10000
5000
0
-5000

WT AUM
WT 5ug/mL vanc
WT 15ug/mL vanc
WT 25ug/mL vanc
saeR AUM
saeR 5ug/mL vanc
saeR 15ug/mL vanc
saeR 25ug/mL vanc

Post-Exponential Phase

30000
25000
20000
15000
10000
5000
0
-5000

WT AUM
WT 5ug/mL vanc
WT 15ug/mL vanc
WT 25ug/mL vanc
saeR AUM
saeR 5ug/mL vanc
saeR 15ug/mL vanc
saeR 25ug/mL vanc

Figure S5: Expression curves of urease in response to various environmental stimuli. Urease expression over a 12 hr time course in the presence of environmental signals of CcpA (**A and B**), SigB (**C and D**), CodY (**E and F**), Agr (**G and H**), SrrA (**I and J**), and SaeR (**K and L**) . Expression was analyzed in cells in exponential phase (**A,C,E,G,I,K**) and post-exponential phase (**B,D,F,H,J,L**). Dots indicate the mean of all replicates and lines indicate the standard deviation.

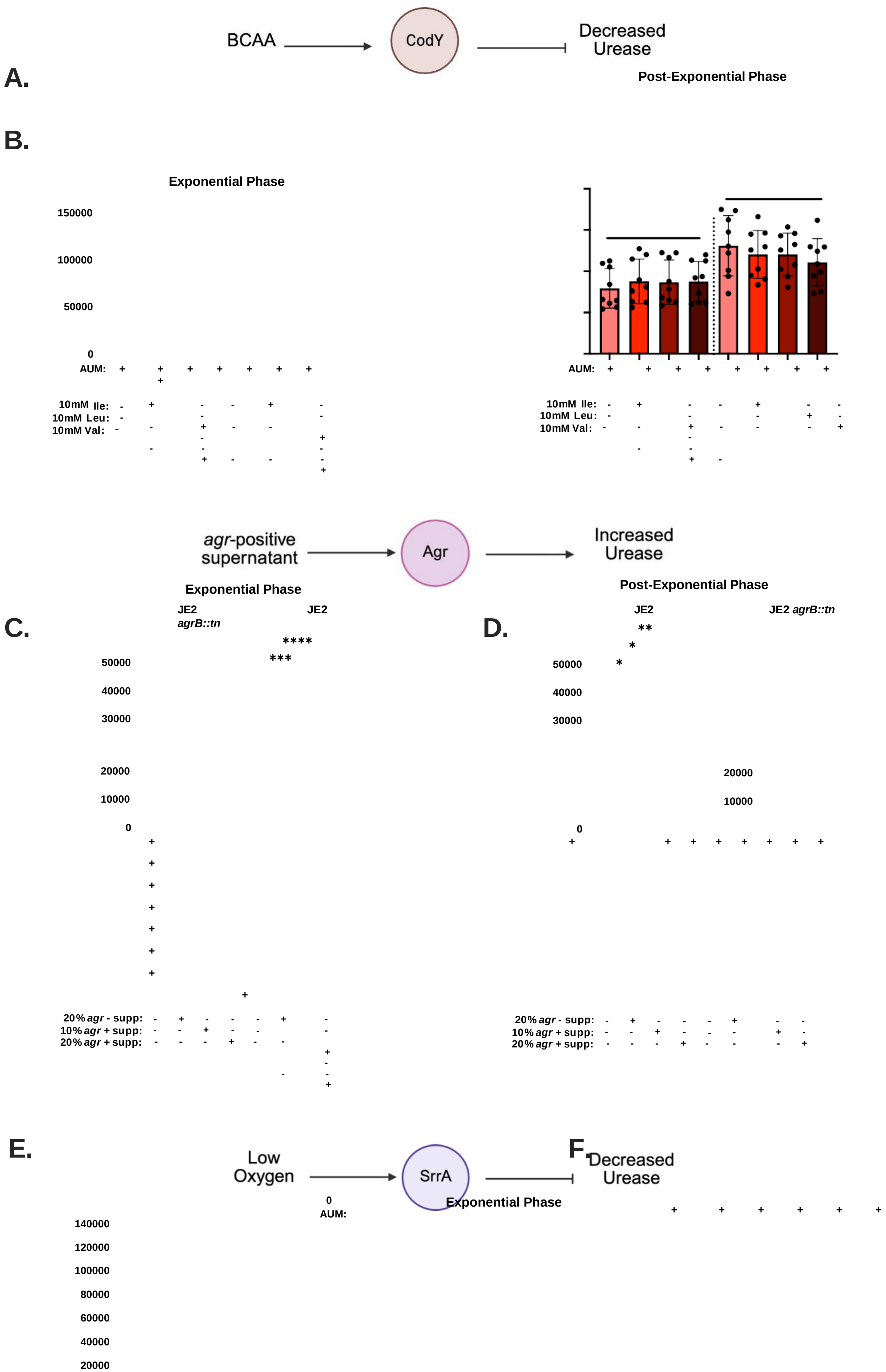

A.
BCAA
CodY
Decreased Urease
B.
Exponential Phase
Post-Exponential Phase
150000
100000
50000
0
AUM:
10mM Ile:
10mM Leu:
10mM Val:
C.
agr-positive supernatant
Agr
Increased Urease
Exponential Phase
JE2
agrB::tn
****
***
50000
40000
30000
20000
10000
0
20% agr - supp:
10% agr + supp:
20% agr + supp:
D.
Post-Exponential Phase
JE2
JE2 agrB::tn
**
*
*
50000
40000
30000
20000
10000
0
E.
Low Oxygen
SrrA
Decreased Urease
F.
Exponential Phase
140000
120000
100000
80000
60000
40000
20000
0
AUM:

Post-Exponential Phase

JE2 *srrA::tn* JE2

140000
120000
100000
80000
60000
40000
20000
0

AUM: + ‡ + + +

0.5mM DEA/NO: - + - -
1mM DEA/NO: - - + + - - -

0.5mM DEA/NO: - + - - + -
1mM DEA/NO: - - + - - +

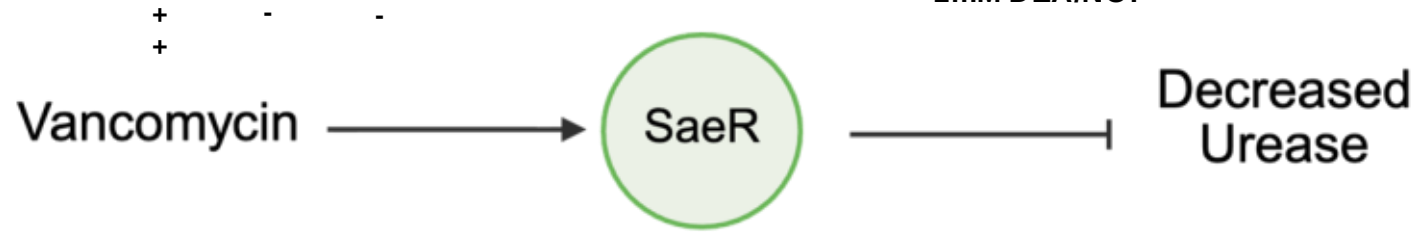

G.

Exponential Phase

150000
100000
50000
0

AUM: + + + + + + + +

5ug/mL vancomycin: - + - - - + -
10ug/mL vancomycin: - - + - - - -
15ug/mL vancomycin: - - - + - - +

H.

Post-Exponential Phase

JE2 JE2 *saeR::tn*

350000
300000
250000
200000
150000
100000
50000
0

AUM: + + + + + + + +

5ug/mL vancomycin: - + - - - + - -
10ug/mL vancomycin: - - + - - - + -
15ug/mL vancomycin: - - - + - - - +

Figure S6: Urease expression in the presence of known regulator signals of CodY, Agr, SrrA, and SaeR. Expression was analyzed in cells in exponential phase (**A,C,E,G**) and post-exponential phase (**B,D,F,H**) after supplementation of increasing concentrations of supernatant with or without branched chain amino acids (CodY activator), auto inducing peptide (Agr activator), DEA/NO (SrrA activator), or vancomycin (SaeR activator). Each dot represents a technical replicate. At least two total biological replicates were performed. Bars represent the mean of all replicates with the standard deviation. Area under the curve analysis was analyzed and significance was determined using the Mann-Whitney U test. Created in BioRender. Gomez, J. (2026) https://BioRender.com/ibh68f3.

B.

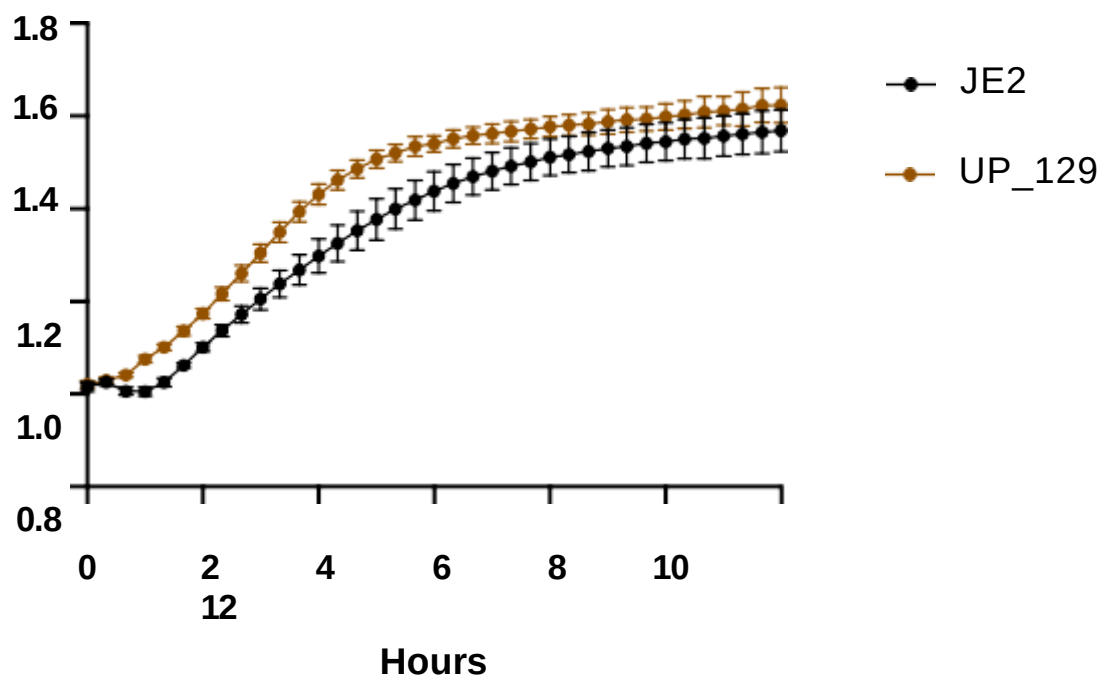

D.

BHI

4000
3000
2000
1000
0
-1000
-2000

JE2
UP_129

E.

AUM

4000
3000
2000
1000
0
-1000
-2000

JE2
UP_129

F.

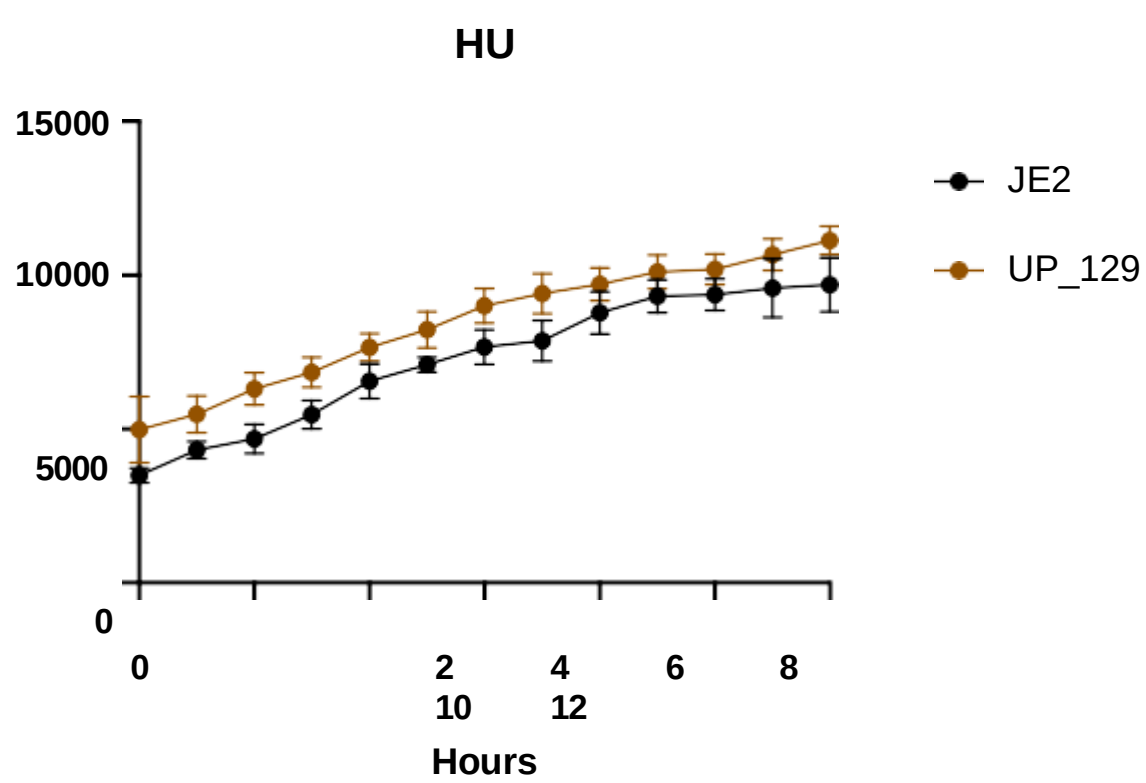

G.

JE2

15000
10000
5000
0
-5000

pUre1 BHI
pUre2 BHI
pUre1 AUM
pUre2 AUM
pUre1 HU
pUre2 HU

H.

UP_129

15000
10000
5000
0
-5000

pUre1 BHI
pUre2 BHI
pUre1 AUM
pUre2 AUM
pUre1 HU
pUre2 HU

Figure S7: Promoter swaps of the urease promoter from JE2 and UP 129 **A)** Alignment of the 200 base pairs upstream of the urease operon (i.e. the urease promoter) for JE2 and ~170 urinary catheter-associated isolates. Dots indicate conservation of a nucleotide to the consensus and letters indicate a variation of nucleotides compared to the consensus. The JE2 sequence (pUre1) was one of the least common sequences found in these isolates, while the UP 129 sequence (pUre2) was the most commonly observed sequence. **B)** Relative urease activity of JE2 and UP 129. Black lines indicate JE2 urease activity and brown lines indicate UP 129 urease activity. **C)** PCR confirmation of the presence of the pUre1 and pUre2 plasmids in JE2 and UP 129. DNA from wild type strains (1), pUre1 strains (2), and pUre2 strains (3) were amplified with the pGFP check primers listed in Table 2. Urease expression over a 12 hr time course of JE2 and UP 129 strains in BHI (**D**), AUM (**E**), and HU (**F**) during the post-exponential phase. Black lines indicate JE2 fluorescence and brown lines indicate UP 129 fluorescence. Promoter swap expression curves for JE2 (**G**) and UP 129 (**H**). Fluorescence was measured in post-exponential phase cultures for 12 hours in either BHI (black lines) and AUM (blue lines). Dots indicate the mean of all replicates and lines indicate the standard deviation.

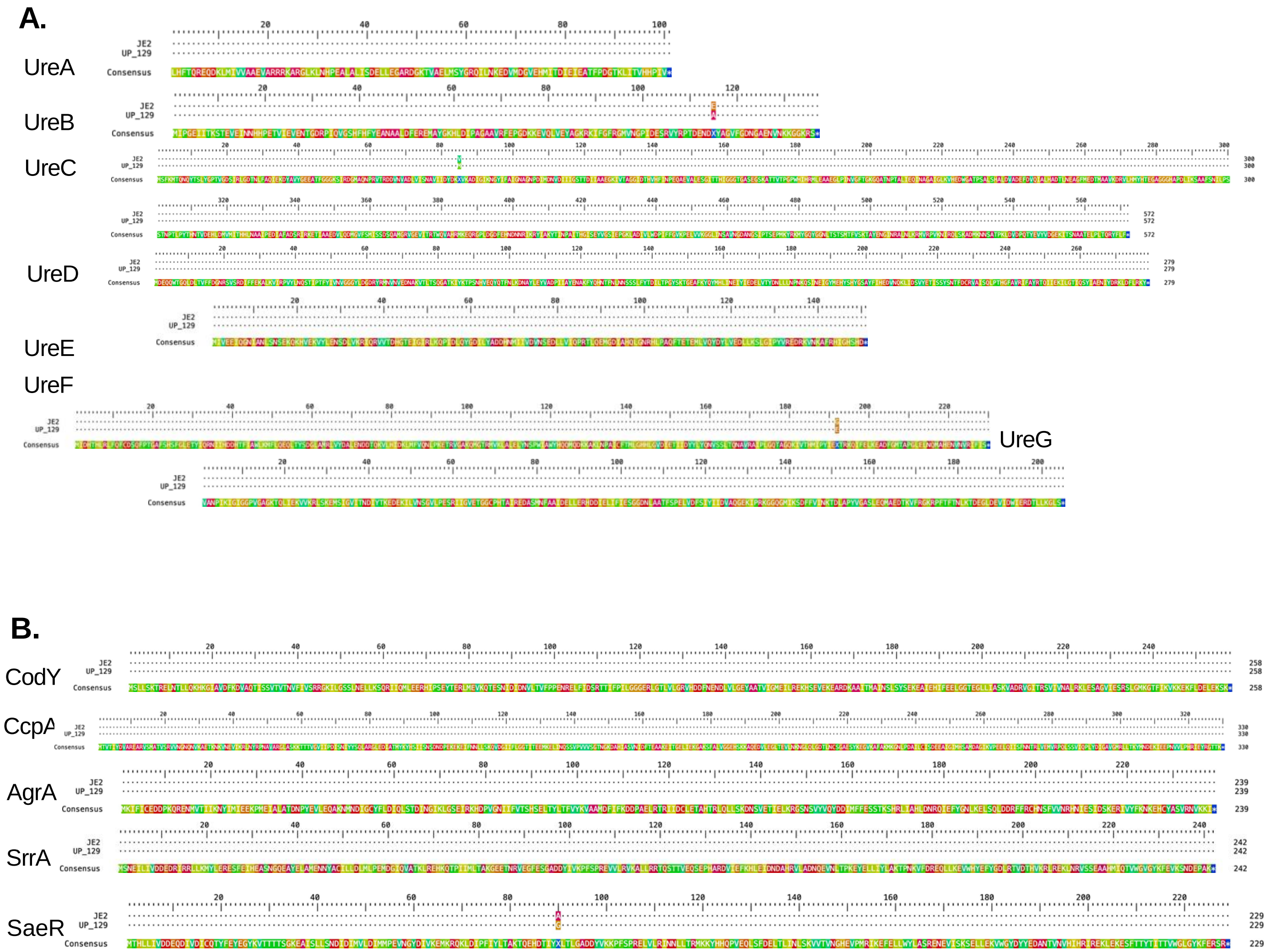

Figure S8: Genomic comparisons of the urease regulation pathway between JE2 and UP 129. **A)** Comparison of the gene sequences of the urease operon in JE2 and UP 129. Dots indicate conserved amino acids between JE2 and UP 129, and letters indicate SNPs resulting in missense mutations of JE2 and UP 129. **B)** Comparison of the protein sequences of CodY, CcpA, AgrA, SrrA, and SaeR in JE2 and UP 129. Dots indeicate conserved amino acids between JE2 and UP 129, and letters indicate SNPs resulting in missense mutations of JE2 and UP 129.

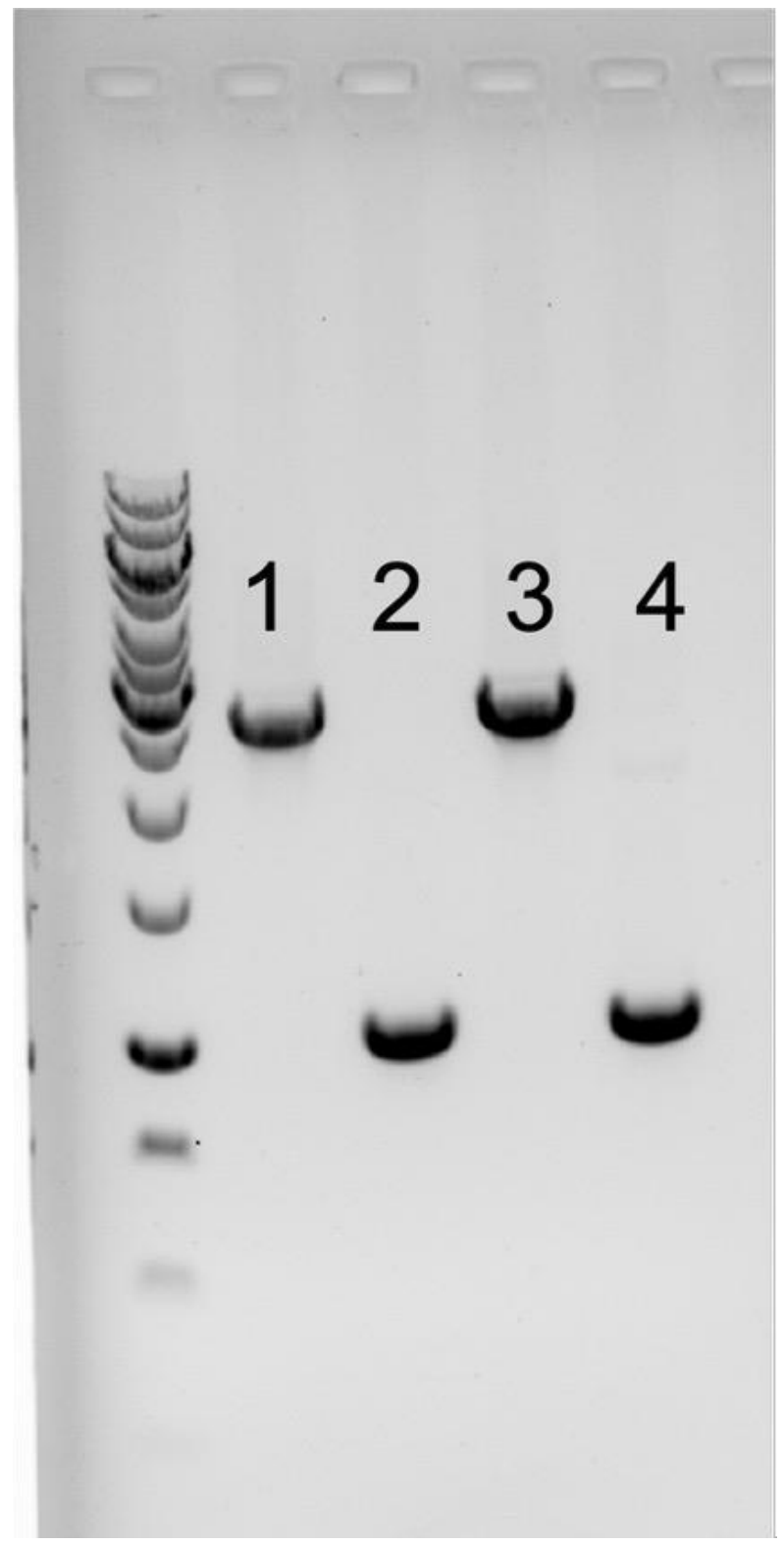

Figure S9: Generation of isogenic *ureC* mutants in the JE2 and UP-129 backgrounds. PCR was performed using the ureC KO upstream F and ureC KO downstream R primers listed in **Table 2.** JE2 wild type (1) and UP 129 wild type (3) had large bands (~1.7kb), indicating a fully intact *ureC* gene, while JE2 Δ*ureC* (2) and UP 129 Δ*ureC* (4) had smaller bands (~0.5kb), indicating a deletion of *ureC.*

S2 File: Sequenced pUre plasmids.

S3 File: List of genomes used in this study.
