# Supplementary material for: Staphylococcus aureus urease is controlled by a complex regulatory network to promote dissemination during CAUTI
Source: mBio. 2026 Aug 5;17(9):e01624-26. doi: 10.1128/mbio.01624-26 (PMC13556217; doi:10.1128/mbio.01624-26)
Supplement: Supplementary file 2 [file mbio.01624-26-s0002.pdf]

Supplemental File 2

>pGFPchloro/erm

Chloramphenicol resistance cassette

Erythromycin resistance cassette

ttgagatcctttttttctgcgcgtaatctgctgcttgcaaacaaaaaaaccaccgctaccagcggtggtttgtttgccggatcaa
gagctaccaactctttttccgaaggtaactggcttcagcagagcgcagataccaaatactgttcttctagtgtagccgtagtta
ggccaccacttcaagaactctgtagcaccgcctacatacctcgctctgctaatcctgttaccagtggctgctgccagtggcg
ataagtcgtgtcttaccgggttggactcaagacgatagttaccggataaggcgcagcggtcgggctgaacggggggttcg
tgcacacagcccagcttggagcgaacgacctacaccgaactgagatacctacagcgtgagctatgagaaagcgccac
gcttcccgaagggagaaaggcggacaggtatccggtaagcggcagggtcggaacaggagagcgcacgagggagct
tccagggggaaacgcctggtatctttatagtcctgtcgggtttcgccacctctgacttgagcgtcgatttttgtgatgctcgtcag
gggggcggagcctatggaaaaacgccagcaacgcggcctttttacggttcctggccttttgctggccttttgctcacatgttct
ttcctgcgttatcccctgattctgtggataaccgtattaccgcctttgagtgagctgataccgctcgccgcagccgaacgacc
gagcgcagcgagtcagtgagcgaggaagcggaagagcgcccaatacgcaaaccgcctctccccgcgcgttggccga
ttcattaatgcagctggcacgacaggtttcccgactggaaagcgggcagtgagcgcaacgcaattaatgtgagttagctc
actcattaggcaccccaggctttacactttatgcttccggctcgtatgttgtgtggaattgtgagcggataacaatttcacacag
gaaacagctatgacatgattacgaattcttagtggtggtggtggtggtgggatcctttgtagagctcatccatgccatgtgtaa
tcccagcagcagttacaaactcaagaaggaccatgtggtcacgcttttcgttaggatctttcgaaaggacagattgtgtcga
caggtaatggttgtctggtaaaaggacagggccatcgccaattggagtattttgttgataatggtctgctagttgaacggaac
catcttcaacgttgtggcgaattttgaagttagctttgattccattcttttgtttgtctgccgtgatgtatacattgtgtgagttaaagtt
gtactcgagtttgtgtccaagaatgtttccatcttctttaaaatcaataccctttaactcgatacgattaacaagggtatcaccttc
aaacttgacttcagcacgcgtcttgtaggtcccgtcatctttgaaagatatagtgcgttcctgtacataaccttcaggcatggc
actcttgaaaaagtcatgacgtttcatgtgatccggataacgggaaaagcattgaacaccataggtcagagtagtgacaa
gtgttggccacggaacaggtagttttccagtagtgcaaataaatttaagggtgagttttccgtttgtagcatcaccttcaccctc
tccacggacagaaaatttgtgcccattaacatcaccatctaattcaacaagaattgggacaactccagtgaaaagttcttct
cctttgctcataaataatcatcctcctaaggtacccggggatccgatgcatcttgctcgatacatttgcccgataatatattgat
atctaatctttatttattatagatatgttagtcataattttgcattaaataagttttattaaatatatttaatgctctattatttagttaattat
aactaattaaaaatgagaagtaaacaaaaaagtgtttataaaacaaattattcattagacacagtgattgtatttctgggtta
gcatttggtttagtcaaaaatatcagaagcttggcactggccgtcgttttacaacgtcgtgactgggaaaaccctggcgttac
ccaacttaatcgccttgcagcacatccccctttcgccagctggcgtaatagcgaagaggcccgcaccgatcgcccttccc
aacagttgcgcagcctgaatggcgaatggcgattatgtcttttgcgcagtcggcttaaaccagttttcgctggtgcgaaaaa
agagtgtcttgtgacactcttaaattcaaaatctatcggtcagatttataccgatttgattttatatattcttgaataacatacgccg
agttatcacataaaagcgggaaccaatcatcaaatttaaacttcattgcataatccattaaactcttaaattctacgattccttg
ttcatcaataaactcaatcatttctttaattaatttatatctatctgttgttgttttctttaataattcatcaacatctacaccgccataa
actatcatatcttctttttgatatttaaatttattaggatcgtccatgtgaagcatatatctcacaagacctttcacacttcctgcaat
ctgcggaatagtcgcattcaattcttctgtaattatttttatctgttcataagatttattaccctcatacatcactagaatatgataat
gctctttttcatcctaccttctgtatcagtatccctatcatgtaatggagacactacaaattgaatgtgtaactcttttaaatactct
aaccactcggcttttgctgattctggatataaaacaaatgtccaattacgtcctcttgaatttttcttgttttcagtttcttttattacatt

ttcgctcatgatataataacggtgctaatacacttaacaaaatttagtcatagataggcagcatgccagtgctgtctatcttttttt
gtttaaaatgcaccgtattcctcctttgcatattttttattagaataccggttgcatctgatttgctaatattatatttttctttgattctatt
taatatctcattttcttctgttgtaagtcttaaagtaacagcaactttttctcttcttttctatctacaaccatcactgtacctcccaac
atctgtttttttcactttaacataaaaaacaaccttttaacattaaaaacccaatatttatttatttgtttggacaatggacaatgga
cacctagggggaggtcgtagtacccccctatgttttctcccctaaataaccccaaaaatctaagaaaaaaagacctcaa
aaaggtctttaattaacatctcaaatttcgcatttattccaatttccttttgcgtgtgatgcgctgcgtccattaaaaatcctagag
ctttgcaaccgaaagttaatagctgtcgctactactttcgcttacgctctaagtatattttaaggactgtcacacgcaaaaagttt
tctcggcataaaagtacctctacatctctaaatcgtctgtacgctgtttctcacgctttctatcgaccttctggacattatcctgtac
aacatccataaactgtcccacacgctcgaatttggaatcattaaagaatttctctttaagcctattaaaccctttctcaaaccca
gggaaattcgccctcgcagcacgatataaagtcactgtactatcttgaaatttctctgatacattcaactgctcattcaaactat
cattctctcgctttaatttattaacctctttactttttcgtgatacccctctttccatgtattcactacttctttcaaactctctctacgttttt
ttaattcttgattttctgtgtaatagtctgtgctcttaatattttcgtaatcatcaacaatccgttctgcagcatattataaaagccagt
cattaggcctatctgacaattcctgaatagagttcataaacaatcctgcatgataaccatcacaaacagaatgatgtacctgt
aaagatagcggtaaatatattgaattacctttattaatgaattttcctgctgtaataatgggtagaaggtaattactattattattg
atatttaagttaaacccagtaaatgaagtccatggaataatagaaagagaaaaagcattttcaggtataggtgttttgggaa
acaatttccccgaaccattatatttctctacatcagaaaggtataaatcataaaactctttgaagtcattctttacaggagtcca
aataccagagaatgttttagatacaccatcaaaaattgtataaagtggctctaacttatcccaataacctaactctccgtcgct
attgtaaccagttctaaaagctgtatttgagtttatcacccttgtcactaagaaaataaatgcagggtaaaatttatatccttcttg
ttttatgtttcggtataaaacactaatatcaatttctgtggttatactaaaagtcgtttgttggttcaaataatgattaaatatctctttt
ctcttccaattgtctaaatcaattttattaaagttcatttgatatgcctcctaaatttttatctaaagtgaatttaggaggcttacttgtc
tgctttcttcattagaatcaatcctttttaaaagtcaatattactgtaacataaatatatattttaaaaatatcccactttatccaattt
tcgtttgttgaactaatgggtgctttagttgaagaataaagtctagaaactccaagctatctttaaaaaatcgttctatttcttctcc
gcttaaatcatcaaagaaatctttatcacttgtaaccagtccgtccacatgtcgaattgcatctgaccgaattttacgtttccctg
aataattctcatcaatcgtttcatcaattttatctttatactttatattttgtgcgttaatcaaatcataatttttatatgtttcctcatgattt
atgtctttattattatagtttttattctctctttgattatgtctttgtatcccgtttgtattacttgatcctttaactctggcaaccctcaaaat
tgaatgagacatgctacacctccggataataaatatatataaacgtatatagatttcataaagtctaacacactagacttattt
acttcgtaattaagtcgttaaaccgtgtgctctacgaccaaaactataaaacctttaagaactttcttttttacaagaaaaaag
aaattagataaatctctcatatcttttattcaataatcgcatccgattgcagtataaatttaacgatcactcatcatgttcatatttat
cagagctcgtgctataattatactaattttataaggaggaaaaaatatgggcatttttagtatttttgtaatcagcacagttcatta
tcaaccaaacaaaaaataagtggttataatgaatcgttaataagcaaaattcatataaccaaattaaagagggttataatg
aacgagaaaaatataaaacacagtcaaaactttattacttcaaaacataatatagataaaataatgacaaatataagatta
aatgaacatgataatatctttgaaatcggctcaggaaaaggccattttacccttgaattagtaaagaggtgtaatttcgtaact
gccattgaaatagaccataaattatgcaaaactacagaaaataaacttgttgatcacgataatttccaagttttaaacaagg
atatattgcagtttaaatttcctaaaaaccaatcctataaaatatatggtaatataccttataacataagtacggatataatacg
caaaattgtttttgatagtatagctaatgagatttatttaatcgtggaatacgggtttgctaaaagattattaaatacaaaacgct
cattggcattacttttaatggcagaagttgatatttctatattaagtatggttccaagagaatattttcatcctaaacctaaagtga
atagctcacttatcagattaagtagaaaaaaatcaagaatatcacacaaagataaacaaaagtataattatttcgttatgaa
atgggttaacaaagaatacaagaaaatatttacaaaaaatcaatttaacaattccttaaaacatgcaggaattgacgattta
aacaatattagctttgaacaattcttatctcttttcaatagctataaattatttaataagtaagttaagggatgcataaactgcatc
ccttaacttgtttttcgtgtgcctatttttgtgaatcgcctgatgcggtattttctccttacgcatctgtgcggtatttcacaccgcata

tggtgcactctcagtacaatctgctctgatgccgcatagttaagccagccccgacacccgccaacacccgctgacgcgcc
ctgacgggcttgtctgctcccggcatccgcttacagacaagctgtgaccgtctccgggagctgcatgtgtcagaggttttcac
cgtcatcaccgaaacgcgcgagacgaaagggcctcgtgatacgcctatttttataggttaatgtcatgataataatggtttctt
agacgtcaggtggcacttttcggggaaatgtgcgcggaacccctatttgtttatttttctaaatacattcaaatatgtatccgctc
atgagacaataaccctgataaatgcttcaataatattgaaaaaggaagagtatgagtattcaacatttccgtgtcgcccttatt
cccttttttgcggcattttgccttcctgtttttgctcacccagaaacgctggtgaaagtaaaagatgctgaagatcagttgggtgc
acgagtgggttacatcgaactggatctcaacagcggtaagatccttgagagttttcgccccgaagaacgttttccaatgatg
agcacttttaaagttctgctatgtggcgcggtattatcccgtattgacgccgggcaagagcaactcggtcgccgcatacact
attctcagaatgacttggttgagtactcaccagtcacagaaaagcatcttacggatggcatgacagtaagagaattatgca
gtgctgccataaccatgagtgataacactgcggccaacttacttctgacaacgatcggaggaccgaaggagctaaccgc
ttttttgcacaacatgggggatcatgtaactcgccttgatcgttgggaaccggagctgaatgaagccataccaaacgacga
gcgtgacaccacgatgcctgtagcaatggcaacaacgttgcgcaaactattaactggcgaactacttactctagcttcccg
gcaacaattaatagactggatggaggcggataaagttgcaggaccacttctgcgctcggcccttccggctggctggtttatt
gctgataaatctggagccggtgagcgtgggtctcgcggtatcattgcagcactggggccagatggtaagccctcccgtatc
gtagttatctacacgacggggagtcaggcaactatggatgaacgaaatagacagatcgctgagataggtgcctcactgat
taagcattggtaactgtcagaccaagtttactcatatatactttagattgatttaaaacttcatttttaatttaaaaggatctaggtg
aagatcctttttgataatctcatgaccaaaatcccttaacgtgagttttcgttccactgagcgtcagaccccgtagaaaagatc
aaaggatcttc

>pUre1ch

Upstream region

gaagatcctttgatcttttctacggggtctgacgctcagtggaacgaaaactcacgttaagggattttggtcatgagattatca
aaaaggatcttcacctagatccttttaaattaaaaatgaagttttaaatcaatctaaagtatatatgagtaaacttggtctgaca
gttaccaatgcttaatcagtgaggcacctatctcagcgatctgtctatttcgttcatccatagttgcctgactccccgtcgtgtag
ataactacgatacgggagggcttaccatctggccccagtgctgcaatgataccgcgagacccacgctcaccggctccag
atttatcagcaataaaccagccagccggaagggccgagcgcagaagtggtcctgcaactttatccgcctccatccagtct
attaattgttgccgggaagctagagtaagtagttcgccagttaatagtttgcgcaacgttgttgccattgctacaggcatcgtg
gtgtcacgctcgtcgtttggtatggcttcattcagctccggttcccaacgatcaaggcgagttacatgatcccccatgttgtgca
aaaaagcggttagctccttcggtcctccgatcgttgtcagaagtaagttggccgcagtgttatcactcatggttatggcagca
ctgcataattctcttactgtcatgccatccgtaagatgcttttctgtgactggtgagtactcaaccaagtcattctgagaatagtgt
atgcggcgaccgagttgctcttgcccggcgtcaatacgggataataccgcgccacatagcagaactttaaaagtgctcat
cattggaaaacgttcttcggggcgaaaactctcaaggatcttaccgctgttgagatccagttcgatgtaacccactcgtgca
cccaactgatcttcagcatcttttactttcaccagcgtttctgggtgagcaaaaacaggaaggcaaaatgccgcaaaaaag
ggaataagggcgacacggaaatgttgaatactcatactcttcctttttcaatattattgaagcatttatcagggttattgtctcatg
agcggatacatatttgaatgtatttagaaaaataaacaaataggggttccgcgcacatttccccgaaaagtgccacctgac
gtctaagaaaccattattatcatgacattaacctataaaaataggcgtatcacgaggccctttcgtctcgcgcgtttcggtgat
gacggtgaaaacctctgacacatgcagctcccggagacggtcacagcttgtctgtaagcggatgccgggagcagacaa
gcccgtcagggcgcgtcagcgggtgttggcgggtgtcggggctggcttaactatgcggcatcagagcagattgtactgag

agtgcaccatatgcggtgtgaaataccgcacagatgcgtaaggagaaaataccgcatcaggcgattcacaaaaaatag
gcacacgaaaaacaagttaagggatgcagtttatgcatcccttaacttacttattaaataatttatagctattgaaaagagat
aagaattgttcaaagctaatattgtttaaatcgtcaattcctgcatgttttaaggaattgttaaattgatttttgtaaatattttcttgt
attctttgttaacccatttcataacgaaataattatacttttgtttatctttgtgtgatattcttgattttttctacttaatctgataagtga
gctattcactttaggtttaggatgaaaatattctcttggaaccatacttaatatagaaatatcaacttctgccattaaaagtaatg
ccaatgagcgttttgtatttaataatcttttagcaaacccgtattccacgattaaataaatctcattagctatactatcaaaaaca
attttgcgtattatatccgtacttatgttataaggtatattaccatatattttataggattggtttttaggaaatttaaactgcaatatat
ccttgtttaaaacttggaaattatcgtgatcaacaagtttattttctgtagttttgcataatttatggtctatttcaatggcagttacga
aattacacctctttactaattcaagggtaaaatggccttttcctgagccgatttcaaagatattatcatgttcatttaatcttatattt
gtcattattttatctatattatgttttgaagtaataaagttttgactgtgttttatatttttctcgttcattataaccctctttaatttggttatat
gaattttgcttattaacgattcattataaccacttatttttgtttggttgataatgaactgtgctgattacaaaaatactaaaaatgc
ccatattttttcctccttataaaattagtataattatagcacgagctctgataaatatgaacatgatgagtgatcgttaaatttata
ctgcaatcggatgcgattattgaataaaagatatgagagatttatctaatttctttttcttgtaaaaaaagaaagttcttaaagg
ttttatagttttggtcgtagagcacacggtttaacgacttaattacgaagtaaataagtctagtgtgttagactttatgaaatctat
atacgtttatatatatttattatccggaggtgtagcatgtctcattcaattttgagggttgccagagttaaaggatcaagtaatac
aaacgggatacaaagacataatcaaagagagaataaaaactataataataaagacataaatcatgaggaaacatata
aaaattatgatttgattaacgcacaaaatataaagtataaagataaaattgatgaaacgattgatgagaattattcagggaa
acgtaaaattcggtcagatgcaattcgacatgtggacggactggttacaagtgataaagatttctttgatgatttaagcggag
aagaaatagaacgatttttaaagatagcttggagtttctagactttattcttcaactaaagcacccattagttcaacaaacga
aaattggataaagtgggatatttttaaaatatatatttatgttacagtaatattgacttttaaaaaaggattgattctaatgaaga
aagcagacaagtaagcctcctaaattcactttagataaaaatttaggaggcatatcaaatgaactttaataaaattgatttag
acaattggaagagaaaagagatatttaatcattatttgaaccaacaaacgacttttagtataaccacagaaattgatattagt
gttttataccgaaacataaaacaagaaggatataaattttaccctgcatttattttcttagtgacaagggtgataaactcaaat
acagcttttagaactggttacaatagcgacggagagttaggttattgggataagttagagccactttatacaatttttgatggtg
tatctaaaacattctctggtatttggactcctgtaaagaatgacttcaaagagttttatgatttatacctttctgatgtagagaaat
ataatggttcggggaaattgtttcccaaaacacctatacctgaaaatgctttttctctttctattattccatggacttcatttactgg
gtttaacttaaatatcaataataatagtaattaccttctacccattattacagcaggaaaattcattaataaaggtaattcaatat
atttaccgctatctttacaggtacatcattctgtttgtgatggttatcatgcaggattgtttatgaactctattcaggaattgtcagat
aggcctaatgactggcttttataatatgctgcagaacggattgttgatgattacgaaaatattaagagcacagactattacac
agaaaatcaagaattaaaaaaacgtagagagagtttgaaagaagtagtgaatacatggaaagaggggtatcacgaaa
aaagtaaagaggttaataaattaaagcgagagaatgatagtttgaatgagcagttgaatgtatcagagaaatttcaagata
gtacagtgactttatatcgtgctgcgagggcgaatttccctgggtttgagaaagggtttaataggcttaaagagaaattcttta
atgattccaaattcgagcgtgtgggacagtttatggatgttgtacaggataatgtccagaaggtcgatagaaagcgtgaga
aacagcgtacagacgatttagagatgtagaggtacttttatgccgagaaaactttttgcgtgtgacagtccttaaaatatactt
agagcgtaagcgaaagtagtagcgacagctattaactttcggttgcaaagctctaggatttttaatggacgcagcgcatca
cacgcaaaaaggaaattggaataaatgcgaaatttgagatgttaattaaagacctttttgaggtctttttttcttagatttttggg
gttatttaggggagaaaacatagggggggtactacgacctccccccctaggtgtccattgtccattgtccaaacaaataaata
aatattgggtttttaatgttaaaaggttgtttttatgttaaagtgaaaaaaacagatgttgggaggtacagtgatggttgtagat
agaaaagaagagaaaaaagttgctgttactttaagacttacaacagaagaaaatgagatattaaatagaatcaaagaa
aaatataatattagcaaatcagatgcaaccggtattctaataaaaaaatatgcaaaggaggaatacggtgcattttaaaca

aaaaaagatagacagcactggcatgctgcctatctatgactaaattttgttaagtgtattagcaccgttattatatcatgagcg
aaaatgtaataaaagaaactgaaaacaagaaaaattcaagaggacgtaattggacatttgttttatatccagaatcagca
aaagccgagtggttagagtatttaaaagagttacacattcaatttgtagtgtctccattacatgatagggatactgatacaga
aggtaggatgaaaaaagagcattatcatattctagtgatgtatgagggtaataaatcttatgaacagataaaaataattaca
gaagaattgaatgcgactattccgcagattgcaggaagtgtgaaaggtcttgtgagatatatgcttcacatggacgatccta
ataaatttaaatatcaaaaagaagatatgatagtttatggcggtgtagatgttgatgaattattaaagaaaacaacaacaga
tagatataaattaattaaagaaatgattgagtttattgatgaacaaggaatcgtagaatttaagagtttaatggattatgcaat
gaagtttaaatttgatgattggttcccgcttttatgtgataactcggcgtatgttattcaagaatatataaaatcaaatcggtataa
atctgaccgatagattttgaatttaagagtgtcacaagacactctttttcgcaccagcgaaaactggtttaagccgactgcgc
aaaagacataatcgccattcgccattcaggctgcgcaactgttgggaagggcgatcggtgcgggcctcttcgctattacgc
cagctggcgaaagggggatgtgctgcaaggcgattaagttgggtaacgccagggttttcccagtcacgacgttgtaaaac
gacggccagtgccaagctttctatgaaaagttatattaaaaactattttggtttttacataaatatattccactatttgttttcgatta
gattgtttgcttcaatatcggatatgctaaaaatataattttctaattacacattgaactttgtatattacttgtttatcacaacaatta
ggattaatataaattttgtaaattgtatctaatatgaaattggggggttttaatggtaccttaggaggatgattatttatgagcaaag
gagaagaacttttcactggagttgtcccaattcttgttgaattagatggtgatgttaatgggcacaaattttctgtccgtggaga
gggtgaaggtgatgctacaaacggaaaactcacccttaaatttatttgcactactggaaaactacctgttccgtggccaaca
cttgtcactactctgacctatggtgttcaatgcttttcccgttatccggatcacatgaaacgtcatgactttttcaagagtgccatg
cctgaaggttatgtacaggaacgcactatatctttcaaagatgacgggacctacaagacgcgtgctgaagtcaagtttgaa
ggtgataccc ttgttaatcgtatcgagttaaagggtattgattttaaagaagatggaaacattcttggacacaaactcgagta
caactttaactcacacaatgtatacatcacggcagacaaacaaaagaatggaatcaaagctaacttcaaaattcgccac
aacgttgaagatggttccgttcaactagcagaccattatcaacaaaatactccaattggcgatggccctgtccttttaccaga
caaccattacctgtcgacacaatctgtcctttcgaaagatcctaacgaaaagcgtgaccacatggtccttcttgagtttgtaa
ctgctgctgggattacacatggcatggatgagctctacaaaggatcccaccaccaccaccaccactaagaattcgtaatc
atgtcatagctgtttcctgtgtgaaattgttatccgctcacaattccacacaacatacgagccggaagcataaagtgtaaagc
ctggggtgcctaatgagtgagctaactcacattaattgcgttgcgctcactgcccgctttccagtcgggaaacctgtcgtgcc
agctgcattaatgaatcggccaacgcgcggggagaggcggtttgcgtattgggcgctcttccgcttcctcgctcactgactc
gctgcgctcggtcgttcggctgcggcgagcggtatcagctcactcaaaggcggtaatacggttatccacagaatcagggg
ataacgcaggaaagaacatgtgagcaaaaggccagcaaaaggccaggaaccgtaaaaaggccgcgttgctggcgtt
tttccataggctccgcccccctgacgagcatcacaaaaatcgacgctcaagtcagaggtggcgaaacccgacaggact
ataaagataccaggcgtttccccctggaagctccctcgtgcgctctcctgttccgaccctgccgcttaccggatacctgtccg
cctttctcccttcgggaagcgtggcgctttctcatagctcacgctgtaggtatctcagttcggtgtaggtcgttcgctccaagctg
ggctgtgtgcacgaaccccccgttcagcccgaccgctgcgccttatccggtaactatcgtcttgagtccaacccggtaaga
cacgacttatcgccactggcagcagccactggtaacaggattagcagagcgaggtatgtaggcggtgctacagagttctt
gaagtggtggcctaactacggctacactagaagaacagtatttggtatctgcgctctgctgaagccagttaccttcggaaa
aagagttggtagctcttgatccggcaaacaaaccaccgctggtagcggtggtttttttgtttgcaagcagcagattacgcgc
agaaaaaaaggatctcaa

>pUre2ch

Upstream region

SNPs in upstream region compared to pUre1ch

gaagatcctttgatcttttctacggggtctgacgctcagtggaacgaaaactcacgttaagggattttggtcatgagattatca
aaaaggatcttcacctagatccttttaaattaaaaatgaagttttaaatcaatctaaagtatatatgagtaaacttggtctgaca
gttaccaatgcttaatcagtgaggcacctatctcagcgatctgtctatttcgttcatccatagttgcctgactccccgtcgtgtag
ataactacgatacgggagggcttaccatctggccccagtgctgcaatgataccgcgagacccacgctcaccggctccag
atttatcagcaataaaccagccagccggaagggccgagcgcagaagtggtcctgcaactttatccgcctccatccagtct
attaattgttgccgggaagctagagtaagtagttcgccagttaatagtttgcgcaacgttgttgccattgctacaggcatcgtg
gtgtcacgctcgtcgtttggtatggcttcattcagctccggttcccaacgatcaaggcgagttacatgatcccccatgttgtgca
aaaaagcggttagctccttcggtcctccgatcgttgtcagaagtaagttggccgcagtgttatcactcatggttatggcagca
ctgcataattctcttactgtcatgccatccgtaagatgcttttctgtgactggtgagtactcaaccaagtcattctgagaatagtgt
atgcggcgaccgagttgctcttgcccggcgtcaatacgggataataccgcgccacatagcagaactttaaaagtgctcat
cattggaaaacgttcttcggggcgaaaactctcaaggatcttaccgctgttgagatccagttcgatgtaacccactcgtgca
cccaactgatcttcagcatcttttactttcaccagcgtttctgggtgagcaaaaacaggaaggcaaaatgccgcaaaaaag
ggaataagggcgacacggaaatgttgaatactcatactcttcctttttcaatattattgaagcatttatcagggttattgtctcatg
agcggatacatatttgaatgtatttagaaaaataaacaaataggggttccgcgcacatttccccgaaaagtgccacctgac
gtctaagaaaccattattatcatgacattaacctataaaaataggcgtatcacgaggccctttcgtctcgcgcgtttcggtgat
gacggtgaaaacctctgacacatgcagctcccggagacggtcacagcttgtctgtaagcggatgccgggagcagacaa
gcccgtcagggcgcgtcagcgggtgttggcgggtgtcggggctggcttaactatgcggcatcagagcagattgtactgag
agtgcaccatatgcggtgtgaaataccgcacagatgcgtaaggagaaaataccgcatcaggcgattcacaaaaaatag
gcacacgaaaaacaagttaagggatgcagtttatgcatcccttaacttacttattaaataatttatagctattgaaaagagat
aagaattgttcaaagctaatattgtttaaatcgtcaattcctgcatgttttaaggaattgttaaattgatttttgtaaatattttcttgt
attctttgttaacccatttcataacgaaataattatacttttgtttatctttgtgtgatattcttgatttttttctacttaatctgataagtga
gctattcactttaggtttaggatgaaaatattctcttggaaccatacttaatatagaaatatcaacttctgccattaaaagtaatg
ccaatgagcgttttgtatttaataatcttttagcaaacccgtattccacgattaaataaatctcattagctatactatcaaaaaca
attttgcgtattatatccgtacttatgttataaggtatattaccatatattttataggattggtttttaggaaatttaaactgcaatatat
ccttgtttaaaacttggaaattatcgtgatcaacaagtttattttctgtagttttgcataatttatggtctatttcaatggcagttacga
aattacacctctttactaattcaagggtaaaatggccttttcctgagccgatttcaaagatattatcatgttcatttaatcttatattt
gtcattattttatctatattatgttttgaagtaataaagttttgactgtgttttatatttttctcgttcattataaccctctttaatttggttatat
gaattttgcttattaacgattcattataaccacttatttttttgtttggttgataatgaactgtgctgattacaaaaatactaaaaatgc
ccatatttttttcctccttataaaattagtataattatagcacgagctctgataaatatgaacatgatgagtgatcgttaaatttata
ctgcaatcggatgcgattattgaataaaagatatgagagatttatctaatttctttttcttgtaaaaaaagaaagttcttaaagg
ttttatagttttggtcgtagagcacacggtttaacgacttaattacgaagtaaataagtctagtgtgttagactttatgaaatctat
atacgtttatatatatttattatccggaggtgtagcatgtctcattcaattttgagggttgccagagttaaaggatcaagtaatac
aaacgggatacaaagacataatcaaagagagaataaaaactataataataaagacataaatcatgaggaaacatata
aaaattatgatttgattaacgcacaaaatataaagtataaagataaaattgatgaaacgattgatgagaattattcagggaa
acgtaaaattcggtcagatgcaattcgacatgtggacggactggttacaagtgataaagatttctttgatgatttaagcggag
aagaaatagaacgatttttaaagatagcttggagtttctagactttattcttcaactaaagcacccattagttcaacaaacga
aaattggataaagtgggatatttttaaaatatatatttatgttacagtaatattgacttttaaaaaaggattgattctaatgaaga
aagcagacaagtaagcctcctaaattcactttagataaaaatttaggaggcatatcaaatgaactttaataaaattgatttag
acaattggaagagaaaagagatatttaatcattatttgaaccaacaaacgacttttagtataaccacagaaattgatattagt

gttttataccgaaacataaaacaagaaggatataaattttaccctgcatttattttcttagtgacaagggtgataaactcaaat
acagcttttagaactggttacaatagcgacggagagttaggttattgggataagttagagccactttatacaatttttgatggtg
tatctaaaacattctctggtatttggactcctgtaaagaatgacttcaaagagttttatgatttatacctttctgatgtagagaaat
ataatggttcggggaaattgtttcccaaaacacctatacctgaaaatgctttttctctttctattattccatggacttcatttactgg
gtttaacttaaatatcaataataatagtaattaccttctacccattattacagcaggaaaattcattaataaaggtaattcaatat
atttaccgctatctttacaggtacatcattctgtttgtgatggttatcatgcaggattgtttatgaactctattcaggaattgtcagat
aggcctaatgactggcttttataatatgctgcagaacggattgttgatgattacgaaaatattaagagcacagactattacac
agaaaatcaagaattaaaaaaacgtagagagagtttgaaagaagtagtgaatacatggaaagaggggtatcacgaaa
aaagtaaagaggttaataaattaaagcgagagaatgatagtttgaatgagcagttgaatgtatcagagaaatttcaagata
gtacagtgactttatatcgtgctgcgagggcgaatttccctgggtttgagaaagggtttaataggcttaaagagaaattcttta
atgattccaaattcgagcgtgtgggacagtttatggatgttgtacaggataatgtccagaaggtcgatagaaagcgtgaga
aacagcgtacagacgatttagagatgtagaggtacttttatgccgagaaaactttttgcgtgtgacagtccttaaaatatactt
agagcgtaagcgaaagtagtagcgacagctattaactttcggttgcaaagctctaggatttttaatggacgcagcgcatca
cacgcaaaaaggaaattggaataaatgcgaaatttgagatgttaattaaagacctttttgaggtctttttttcttagatttttggg
gttatttaggggagaaaacataggggggtactacgacctccccccctaggtgtccattgtccattgtccaaacaaataaata
aatattgggtttttaatgttaaaaggttgtttttatgttaaagtgaaaaaaacagatgttgggaggtacagtgatggttgtagat
agaaaagaagagaaaaaagttgctgttactttaagacttacaacagaagaaaatgagatattaaatagaatcaaagaa
aaatataatattagcaaatcagatgcaaccggtattctaataaaaaaatatgcaaaggaggaatacggtgcattttaaaca
aaaaaagatagacagcactggcatgctgcctatctatgactaaattttgttaagtgtattagcaccgttattatatcatgagcg
aaaatgtaataaaagaaactgaaaacaagaaaaattcaagaggacgtaattggacatttgttttatatccagaatcagca
aaagccgagtggttagagtatttaaaagagttacacattcaatttgtagtgtctccattacatgatagggatactgatacaga
aggtaggatgaaaaaagagcattatcatattctagtgatgtatgagggtaataaatcttatgaacagataaaaataattaca
gaagaattgaatgcgactattccgcagattgcaggaagtgtgaaaggtcttgtgagatatatgcttcacatggacgatccta
ataaatttaaatatcaaaaagaagatatgatagtttatggcggtgtagatgttgatgaattattaaagaaaacaacaacaga
tagatataaattaattaaagaaatgattgagtttattgatgaacaaggaatcgtagaatttaagagtttaatggattatgcaat
gaagtttaaatttgatgattggttcccgcttttatgtgataactcggcgtatgttattcaagaatatataaaatcaaatcggtataa
atctgaccgatagattttgaatttaagagtgtcacaagacactctttttcgcaccagcgaaaactggtttaagccgactgcgc
aaaagacataatcgccattcgccattcaggctgcgcaactgttgggaagggcgatcggtgcgggcctcttcgctattacgc
cagctggcgaaagggggatgtgctgcaaggcgattaagttgggtaacgccagggttttcccagtcacgacgttgtaaaac
gacggccagtgccaagctttctatgaaaagttatattaaaaactattttggtttttacataaatatattcc**g**ctatttgttttcgatta
gattgtttgcttcaata**g**cggatatgctaaaaatataattttctaattacacattgaactttgtatattacttgtttatcacaacaatt
aggattaatataaattttgtaaattgtatctaatatgaaattggggggttttaatggtaccttaggaggatgattatttatgagcaaa
ggagaagaacttttcactggagttgtcccaattcttgttgaattagatggtgatgttaatgggcacaaattttctgtccgtggag
agggtgaaggtgatgctacaaacggaaaactcacccttaaatttatttgcactactggaaaactacctgttccgtggccaac
acttgtcactactctgacctatggtgttcaatgcttttcccgttatccggatcacatgaaacgtcatgactttttcaagagtgccat
gcctgaaggttatgtacaggaacgcactatatctttcaaagatgacgggacctacaagacgcgtgctgaagtcaagtttga
aggtgatacccttgttaatcgtatcgagttaaagggtattgattttaaagaagatggaaacattcttggacacaaactcgagt
acaactttaactcacacaatgtatacatcacggcagacaaacaaaagaatggaatcaaagctaacttcaaaattcgcca
caacgttgaagatggttccgttcaactagcagaccattatcaacaaaatactccaattggcgatggccctgtccttttaccag
acaaccattacctgtcgacacaatctgtcctttcgaaagatcctaacgaaaagcgtgaccacatggtccttcttgagtttgta

actgctgctgggattacacatggcatggatgagctctacaaaggatcccaccaccaccaccaccactaagaattcgtaat
catgtcatagctgtttcctgtgtgaaattgttatccgctcacaattccacacaacatacgagccggaagcataaagtgtaaag
cctggggtgcctaatgagtgagctaactcacattaattgcgttgcgctcactgcccgctttccagtcgggaaacctgtcgtgc
cagctgcattaatgaatcggccaacgcgcggggagaggcggtttgcgtattgggcgctcttccgcttcctcgctcactgact
cgctgcgctcggtcgttcggctgcggcgagcggtatcagctcactcaaaggcggtaatacggttatccacagaatcaggg
gataacgcaggaaagaacatgtgagcaaaaggccagcaaaaggccaggaaccgtaaaaaggccgcgttgctggcg
tttttccataggctccgcccccctgacgagcatcacaaaaatcgacgctcaagtcagaggtggcgaaacccgacaggac
tataaagataccaggcgtttccccctggaagctccctcgtgcgctctcctgttccgaccctgccgcttaccggatacctgtcc
gcctttctcccttcgggaagcgtggcgctttctcatagctcacgctgtaggtatctcagttcggtgtaggtcgttcgctccaagct
gggctgtgtgcacgaaccccccgttcagcccgaccgctgcgccttatccggtaactatcgtcttgagtccaacccggtaag
acacgacttatcgccactggcagcagccactggtaacaggattagcagagcgaggtatgtaggcggtgctacagagttct
tgaagtggtggcctaactacggctacactagaagaacagtatttggtatctgcgctctgctgaagccagttaccttcggaaa
aagagttggtagctcttgatccggcaaacaaaccaccgctggtagcggtggtttttttgtttgcaagcagcagattacgcgc
agaaaaaaaggatctcaa
